# The Metabolite Profiling of *Aspergillus fumigatus* KMM4631 and Its Co-Cultures with Other Marine Fungi

**DOI:** 10.3390/metabo13111138

**Published:** 2023-11-08

**Authors:** Anton N. Yurchenko, Liliana E. Nesterenko, Roman S. Popov, Natalya N. Kirichuk, Viktoria E. Chausova, Ekaterina A. Chingizova, Marina P. Isaeva, Ekaterina A. Yurchenko

**Affiliations:** G.B. Elyakov Pacific Institute of Bioorganic Chemistry, Far Eastern Branch of the Russian Academy of Sciences, Prospect 100-Letiya Vladivostoka, 159, Vladivostok 690022, Russia; nesterenko_le@piboc.dvo.ru (L.E.N.); popov_rs@piboc.dvo.ru (R.S.P.); sheflera@bk.ru (N.N.K.); v.chausova@gmail.com (V.E.C.); martyyas@mail.ru (E.A.C.); issaeva@piboc.dvo.ru (M.P.I.)

**Keywords:** *Aspergillus fumigatus*, phylogeny, identification, secondary metabolites, co-cultivation, UPLC MS qTOF

## Abstract

An *Aspergillus fumigatus* KMM 4631 strain was previously isolated from a Pacific soft coral *Sinularia* sp. sample and was found to be a source of a number of bioactive secondary metabolites. The aims of this work are the confirmation of this strain’ identification based on ITS, *BenA*, *CaM*, and *RPB2* regions/gene sequences and the investigation of secondary metabolite profiles of *Aspergillus fumigatus* KMM 4631 culture and its co-cultures with *Penicillium hispanicum* KMM 4689, *Amphichorda* sp. KMM 4639, *Penicillium* sp. KMM 4672, and *Asteromyces cruciatus* KMM 4696 from the Collection of Marine Microorganisms (PIBOC FEB RAS, Vladivostok, Russia). Moreover, the DPPH-radical scavenging activity, urease inhibition, and cytotoxicity of joint fungal cultures’ extracts on HepG2 cells were tested. The detailed UPLC MS qTOF investigation resulted in the identification and annotation of indolediketopiperazine, quinazoline, and tryptoquivaline-related alkaloids as well as a number of polyketides (totally 20 compounds) in the extract of *Aspergillus fumigatus* KMM 4631. The metabolite profiles of the co-cultures of *A. fumigatus* with *Penicillium hispanicum*, *Penicillium* sp., and *Amphichorda* sp. were similar to those of *Penicillium hispanicum*, *Penicillium* sp., and *Amphichorda* sp. monocultures. The metabolite profile of the co-culture of *A. fumigatus* with *Asteromyces cruciatus* differed from that of each monoculture and may be more promising for the isolation of new compounds.

## 1. Introduction

Marine microbial ecosystems are characterized by high competition and an uneven ratio between prokaryotes and eukaryotes. Studies of the microbiome of marine communities are often limited to prokaryotes, and only a few relevant papers include studies of both prokaryotes and eukaryotes. Their results show that against the background of a large diversity of species of bacteria and archaea, fungi are represented by only a few phyla [1,2]. This interspecific competition for resources stimulates the production of various secondary metabolites by all members of the community. Moreover, a marine bacteria–fungus symbiosis was discovered, in which the bacterium can stimulate the fungus to produce an antibiotic, spiromarmycin, to protect against other bacteria [3].

Understanding the interactions of microbial communities led to the development of chemical ecology-related cultivation-based strategies, now called the “one strain many compounds” (OSMAC) approach, in which the microbial co-culture is used to stimulate the production of new metabolites [4]. One of the first reports was about the isolation of new diketopiperazines glionitrins A and B from a laboratory co-culture of *Aspergillus fumigatus* fungal strain KMC-901 and *Sphingomonas* bacterial strain KMK-001 [5,6]. Glionitrin A had high antibacterial and cytotoxic activity [5], while glionitrin B inhibited the invasion of DU145 cancer cells [6]. This example confirms the leading role of microbial exolites in regulating relationships in microbial communities.

For about two decades, this approach has been successfully used to obtain a variety of low-molecular weight compounds from the co-culture of terrestrial and marine microorganisms. Caudal et al., from 2010 to 2020, reviewed three papers on marine fungi–bacteria co-cultivation, 11 papers on marine bacteria–bacteria co-cultivation, and 14 papers on two marine fungal strain co-cultures [7]. Co-culture has the potential to induce the production of novel metabolites, increase the yield of specific target metabolites with pharmacological potential, or inhibit the production of metabolites found in axenic culture.

Since the diversity of secondary metabolites allows fungi to adapt more or less successfully to various environmental conditions, in some particularly successful cases fungi can become pathogenic to humans. This happens with the widespread *Aspergillus fumigatus*. *A. fumigatus* is a saprotrophic fungus that is pathogenic in highly immunocompromised patients because it is found in high concentrations in the atmosphere; it grows faster than any other airborne fungi at 40 °C [8]. The *A. fumigatus* genome sequence and a metabolomics analysis revealed the potential for synthesizing more than 200 compounds and the presence of over 30 gene clusters associated with secondary metabolites. The non-ribosomal peptide gliotoxin, anthraquinone-derived trypacidin, meroterpenoids fumagillin and pyripyropene A, heteropolymer dihydroxynaphthalene melanin, tryptophan-derived indole alkaloids fumigaclavines, diketopiperazine alkaloids fumitremorgins and verruculogen, and fusidane-type triterpenoid helvolic acid are the primary described secondary metabolites which provide high environmental stability and defensive function against (microbial) predators [9]. However, the comparative investigation of the secondary metabolite profile of several *A. fumigatus* strains isolated from human as well as soil samples showed that soil-derived *A. fumigatus* conidia and culture media did not produce toxic compounds as toxic as fumitremorgins A–C, pyripyropenes E and O, fumigaclavines A–C, helvolic acid, and pseurotins A, B, D [10].

*A. fumigatus* fungi are also common in a variety of marine ecosystems. The marine fungus *Aspergillus fumigatus* KMM4631 associated with the soft coral *Sinularia* sp. was reported as a producer of a number of diketopiperazine and quinazoline alkaloids as well as triterpenoids, and some of them show promising bioactivity [11,12,13]. 

Moreover, promising marine fungal strains such as *Penicillium hispanicum* KMM 4689, *Amphichorda* sp. KMM 4639, *Penicillium* sp. KMM 4672, and *Asteromyces cruciatus* KMM 4696 were found and stored in the Collection of Marine Microorganisms (KMM, Vladivostok, Russia). Recently, some new secondary metabolites were isolated from the co-culture of *Amphichorda* sp. KMM 4639 with *Aspergillus carneus* KMM 4638 [14] and continuing this investigation is promising.

There are several methods of metabolomic analysis, including NMR, GC-MS, LC-MS, and FTIR (Fourier transform infrared spectroscopy), each of which has its own limitations and capabilities. At the same time, the LC-MS method has the best combination of simplicity, speed, selectivity, and repeatability, and therefore is most widely used for the analysis of metabolomes [15].

The aim of the present work is the investigation of the secondary metabolite profiles of *Aspergillus fumigatus* KMM 4631 culture and its co-cultures with *Penicillium hispanicum* KMM 4689, *Amphichorda* sp. KMM 4639, *Penicillium* sp. KMM 4672, and *Asteromyces cruciatus* KMM 4696, using the UPLC-MS-qTOF technique. Moreover, the DPPH-radical scavenging activity, urease inhibition, and cytotoxicity of joint fungal cultures’ extracts on HepG2 cells were tested.

## 2. Materials and Methods

### 2.1. General

An Olympus CX41 microscope (Olympus Corporation, Tokyo, Japan) equipped with an Olympus SC30 camera (Olympus Corporation, Tokyo, Japan) was used for the examination of fungal cultures and the preparation of photography.

### 2.2. Fungal Strains

The fungal strain KMM 4631 was isolated from soft coral *Sinularia* sp. collected near Kunashir Island (Kuril islands, north west Pacific Ocean) and identified based on morphological features as *Aspergillus fumigatus* [11]. The fungal strain KMM 4639 was isolated from a sediment sample collected in Van Phong Bay (the South China Sea, Vietnam) and identified based on molecular features as *Amphichorda* sp. [14]. The fungal strain KMM 4672 was isolated from brown algae *Padina* sp. collected in Van Phong Bay (the South China Sea, Vietnam) and identified based on molecular features as *Penicillium* sp. [16]. The fungal strain KMM 4689 was isolated from identified soft coral collected near Con Co Island (the South China Sea, Vietnam) and identified based on molecular features as *Penicillium hispanicum* [17]. The fungal strain KMM 4696 was isolated from brown algae *Sargassum pallidum* (Vostok Bay, the Sea of Japan) and identified based on molecular features as *Asteromyces cruciatus* [18]. 

All used fungal strains are stored in the Collection of Marine Microorganisms (PIBOC FEB RAS, Vladivostok, Russia). The strain *Penicillium hispanicum* KMM 4689 also stored in the collection of the Nhatrang Institute of Technology Research and Applications under the code VO49-30.5. 

### 2.3. DNA Extraction and Amplification

The fungal mycelia (mycelium) grew on MEA (malt extract agar) at 25 °C for 7 days, and then genomic DNA was isolated using the MagJET Plant Genomic DNA Kit (Thermo Fisher Scientific, Waltham, MA, USA), according to the manufacturer’s protocol. PCR was conducted using GoTaq Flexi DNA Polymerase (Promega, Madison, WI, USA). The ITS region and partial *BenA* gene were amplified and sequenced as described in [14]. For amplification of the partial *CaM* gene, the standard primer pair cmd5 and cmd6 was used [19]. The reaction profile was 95 °C for 300 s, 35 cycles of 95 °C for 30 s, 55 °C for 45 s, and 72 °C for 90 s, and finally 72 °C for 300 s. For amplification of the partial *RPB2* gene, the standard primer pair *RPB2*-5F and *RPB2*-7CR was used [20]. The reaction profile was an initial denaturation at 95 °C for 300 s, followed by 5 cycles at 95 °C for 30 s, 60 °C for 45 s, 72 °C for 120 s, then 5 cycles at 95 °C for 30 s, 58 °C for 45 s, 72 °C for 120 s, and finally 30 cycles of 95 °C for 30 s, 54 °C for 45 s, 72 °C for 120 s, and a final elongation at 72 °C for 420 s. The amplified partial *CaM* and *RPB2* genes were purified and sequenced as described in [14]. Gene sequences were deposited in GenBank under accession numbers OR578448 for ITS, OQ466614 for the partial *BenA*, OR600992 for the partial *CaM*, and OR600993 for the partial *RPB2* (Table 1).

### 2.4. Phylogenetic Analysis

The ITS region, the partial *BenA*, *CaM*, and *RPB2* gene sequences of the fungal strain KMM 4631 and members of genus *Aspergillus* section *Fumigati* series *Fumigati* were aligned via MEGA X software version 11.0.9 [21] using the Clustal W algorithm. The ex-type homologs were searched in the GenBank database (http://ncbi.nlm.nih.gov, accessed on 7 September 2023) using the BLASTN algorithm (http://www.ncbi.nlm.nih.gov/BLAST, accessed on 14 September 2023). The phylogenetic analysis was carried out using MEGA X software [21]. The ITS region and partial *BenA*, *CaM*, and *RPB2* gene sequences were concatenated into one alignment. A phylogenetic tree was constructed according to the Maximum Likelihood (ML) algorithm based on the Kimura 2-parameter model [22]. The tree topology was evaluated via 1000 bootstrap replicates. The *Talaromyces marneffei* CBS 388.87^T^ was used as outgroup (Table 1).

### 2.5. Cultivation of Fungi

Before co-cultivation, fungal strains were grown in test tubes on slanted wort agar in sea water for 7 days at 22 °C. Pairs of fungal strains were inoculated onto rice medium simultaneously by transferring a small block of agar medium containing mycelium and conidia. Inoculation of each strain in a pair was carried out at three points and at some distance from each other. Co-cultivation was carried out for 21 days at 22 °C in 500 mL Erlenmeyer flasks, each containing natural sea water (Vodolaznaya bay, Troitsa bay, the Sea of Japan), rice (20.0 g), yeast extract (20.0 mg), and KH_2_PO_4_ (10 mg). Fungal monocultures were obtained in a similar manner.

### 2.6. Extraction and HPLC MS Analysis

#### 2.6.1. Extraction of Fungal Cultures

Each fungal culture with medium was extracted using EtOAc (100 mL) and then evaporated in vacuo to prepare a crude extract (Table 2). Then, each extract was dissolved in methanol and passed through column with C_18_-SiO_2_ (YMC Gel ODS-A, 12 nm, S—75 µm, YMC Co., Ishikawa, Japan). 

The masses of the purified extracts are presented in Table 2.

#### 2.6.2. HPLC MS Analysis of Fungal Extracts

HPLC MS analysis was performed using a Bruker Elute UHPLC chromatograph (Bruker Daltonics, Bremen, Germany) connected to a Bruker Impact II Q-TOF mass spectrometer (Bruker Daltonics, Bremen, Germany). An InfinityLab Poroshell 120 SB-C18 column (2.1 × 150 mm, 2.7 μm, Agilent Technologies, Santa Clara, CA, USA) was used for chromatographic separation. Chromatographic separation and mass spectrometric detection were performed as previously described [17].

#### 2.6.3. UHPLC-Q-TOF Data Analysis

UHPLC-Q-TOF data were converted from Bruker “.d” formatting to “.mzXML” using MSConvert 3.0 (part of ProteoWizard 3.0 package, Palo Alto, CA, USA) [23], and further processing was performed using MZMine (version 2.53) [24] as described previously [17]. 

Metabolite dereplication was also carried out with an in-house MS/MS spectral library, comparing experimental spectra and retention times (RTs) with the spectra and RTs obtained for reference compounds. A set of compounds previously isolated from *Aspergillus fumigatus* KMM 4631, *Penicillium hispanicum* KMM 4689, *Amphichorda* sp. KMM 4639, *Penicillium* sp. KMM 4672, and *Asteromyces cruciatus* KMM 4696 was used as standards for the microbial secondary metabolites. Structures of these compounds were established using various methods including 1D and 2D NMR spectra. Compounds were dissolved in 75% MeCN (0.1 μg/mL) and 3 μL of solution was subjected to UHPLC-Q-TOF MS analysis in the same condition as the studied samples. The full list of the standards for the microbial secondary metabolites is given in the Supplementary Materials.

In addition, the annotation of some metabolites was performed by comparing the experimental MS/MS spectra with compounds from the PubChem database using in-silica fragmentation via the MetFrag service [25]. 

### 2.7. Principal Component Analysis (PCA)

PCA analysis, a hierarchical dendrogram, and visualization of the resulting graphs were performed using the “google colab” web resource based on Python 3.8 using Pandas, Seaborn, and Matplotlib libraries. Below is a link to the notepad with the code used in the analysis: https://drive.google.com/drive/folders/1qov-yZHRKp-L3Qq69iiLTakfzxGOpBYI?usp=drive_link (accessed on 7 September 2023).

### 2.8. Bioassays

#### 2.8.1. Urease Inhibition Assay

The inhibitory activity of the extracts on urease (from *Canavalia ensiformis*, 1U final concentration) was estimated by determining ammonia production using the indophenol method. A reaction mixture consisting of 25 µL enzyme solution and 5 µL of extracts (100.0 µg/mL final concentration) was preincubated at 37 °C for 60 min in 96-well plates. Next, 55 µL of phosphate-buffered solution with 100 µM urea was added to each well and incubated at 37 °C for 10 min. Next, 45 µL of phenol reagent (1% *w*/*v* phenol and 0.005% *w*/*v* sodium nitroprusside) and 70 µL of alkali reagent (0.5% *w*/*v* NaOH and 0.1% active chloride NaClO) were added to each well for 50 min. The pH was maintained at 7.3–7.5 in all assays. DMSO (5%) was used as a positive control. Optical density at 630 nm was measured after 50 min at 630 nm using a MultiskanFS microplate reader (Thermo Scientific Inc., Beverly, MA, USA). 

#### 2.8.2. DPPH Radical Scavenger Assay

DPPH (Sigma-Aldrich, Steinheim, Germany) solution at a concentration of 7.5 × 10^−3^ M was used for this assay. The concentrations of the test extracts in the mixtures were 100 µg/mL. The mixtures were shaken and left to stand for 30 min, and the absorbance of the resulting solutions was measured at 520 nm using a microplate reader MultiscanFC (Thermo Scientific, USA). The radical scavenging activity of the extracts was presented as % to the control (MeOH).

#### 2.8.3. Cell Culture

The human hepatocarcinoma HepG2 cells were purchased from ATCC (Manassas, VA, USA). The cells were cultured in DMEM with 10% of fetal bovine serum and 1% of penicillin/streptomycin (BioloT, St. Petersburg, Russia). For experiments, HepG2 cells were seeded at concentrations of 5 × 10^3^ cell/well and the experiments were started after 24 h.

#### 2.8.4. Cell Viability Assay

The cells were treated with the extracts at a concentration of 10 µg/mL for 24 h, and cell viability was measured using an MTT (3-(4,5-dimethylthiazol-2-yl)-2,5-diphenyltetrazolium bromide) assay, which was performed according to the manufacturer’s instructions (Sigma-Aldrich, St.-Louis, MO, USA). Optical density at 570 nm was detected using a MultiskanFS microplate reader (Thermo Scientific Inc., Beverly, MA, USA). The results were presented as percentages of the control (vehicle) data.

#### 2.8.5. Statistical Data Evaluation

All bioassay data were obtained in three independent replicates, and the calculated values are expressed as a mean ± standard error mean (SEM). Student’s *t*-test was performed using SigmaPlot 14.0 (Systat Software Inc., San Jose, CA, USA) to determine statistical significance. Differences were considered statistically significant at *p* < 0.05.

## 3. Results

### 3.1. Molecular Identification of the Fungal Strain

**Figure 1:**
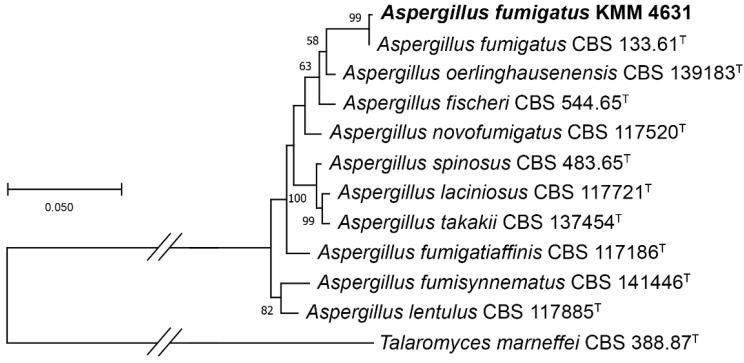
ML tree based on concatenated ITS-*BenA*-*CaM-RPB2* gene sequences showing the phylogenetic position of the strain KMM 4631 among members of genus *Aspergillus* section *Fumigati* series *Fumigati*. Bootstrap values (%) of 1000 replications. Nodes with confidence values greater than 50% are indicated. The scale bars represent 0.05 substitutions per site. ^T^—ex-type strain.

To clarify the taxonomic position of the strain KMM 4631 we sequenced the molecular markers, such as ITS, the partial *BenA*, *CaM*, and *RPB2* regions. Approximately 1300 bp fragments of the ITS region, about 650 bp fragments of the partial *BenA*, and about 650 bp and 1200 bp fragments of the *CaM* and *RPB2* genes, respectively, were successfully amplified. A BLAST search showed that the ITS region, the partial *CaM*, and *RPB2* gene sequences were 100% identical with the sequences of the ex-type strain *Aspergillus fumigatus* CBS 133.61^T^, while the partial *BenA* gene sequence was more than 99% identical. Phylogenetic ML tree of the concatenated ITS-*BenA*-*CaM-RPB2* gene sequences clearly showed that the strain KMM 4631 clusters with the ex-type strain *Aspergillus fumigatus* CBS 133.61^T^ (Figure 1).

### 3.2. Aspergillus fumigatus KMM 4631 Monoculture Metabolites

**Figure 2:**
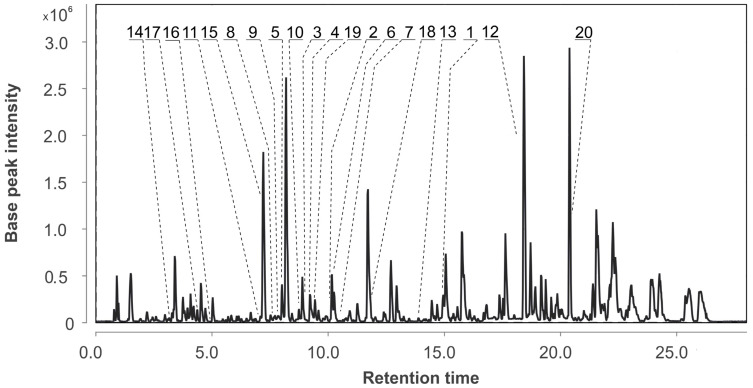
UHPLC MS chromatogram of *Aspergillus fumigatus* KMM 4631 monoculture extract.

The UHPLC MS chromatogram of the extract of *Aspergillus fumigatus* KMM 4631 monoculture (Af) is presented in Figure 2.

**Figure 3:**
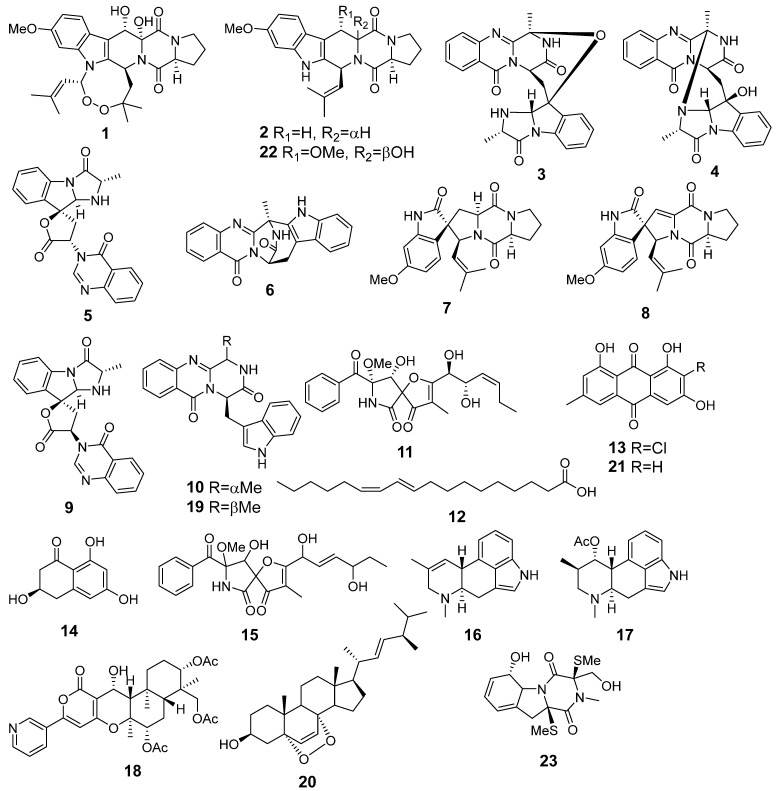
The secondary metabolites detected in *Aspergillus fumigatus* KMM 4631 monoculture.

In total, 20 compounds were identified in the extract of the monoculture of *A. fumigatus* using an in-house database or proposed based on MetFrag and the GNPS database (Figure 3, Table A1). The detailed characteristics of the annotated compounds are presented in Appendix A (Table A1).

The peak detected at 3.2 min (*m*/*z* 195.0649) corresponded to the molecular formula C_10_H_10_O_4_, the same as scytalone (**14**), which was suggested based on MS/MS comparison using MetFrag [25]. The peaks detected at 4.2 (*m*/*z* 299.1759) and 4.8 min (*m*/*z* 239.1519) corresponded to the molecular formulas C_18_H_22_N_2_O_2_ and C_16_H_18_N_2_, the same as fumigaclavine A (**17**) and agroclavine (**16**), respectively; those were suggested based on MS/MS comparison using MetFrag [25].

The peaks detected at 7.1 and 7.2 min (*m*/*z* 432.1664) corresponded to the molecular formula C_22_H_25_NO_8_, the same as isomeric pseurotin A (**11**) and D (**15**), and were suggested based on MS/MS comparison using GNPS database and MetFrag, respectively. The peaks detected at 7.6 and 10.4 with *m*/*z* 394.1754 and *m*/*z* 396.1920 were identified as 6-methoxyspirotriprostatin B (**8**) and spirotriprostatin A (**7**), respectively, based on an exact mass value and RT comparison with an in-house database.

The peaks detected at 7.8 and 8.0 min with *m*/*z* 403.1396 corresponded to the molecular formula C_22_H_18_N_4_O_4_, which can be associated with the stereoisomeric compounds tryptoquivaline F (**9**) and tryptoquivaline J (**5**). The compounds were identified based on an exact mass value and MS/MS with an in-house database and MetFrag [25].

The peak detected at 8.7 min (*m*/*z* 359.1494) corresponded to the molecular formula C_21_H_18_N_4_O_2_ and was suggested to be fumiquinazoline F (**10**) via the GNPS database. The peaks detected at 9.0 and 9.2 min with *m*/*z* 444.1671 corresponded to the molecular formula C_24_H_21_N_5_O_4_, which can be associated with the isomeric quinazoline-containing indole alkaloids fumiquinazoline C (**3**) and fumiquinazoline D (**4**). The compounds were identified based on an exact mass value and MS/MS with an in-house database and GNPS (MQScore 0.95).

The peak detected at 9.4 min (*m*/*z* 359.1480) corresponded to the molecular formula C_21_H_18_N_4_O_2_, the same as fumiquinazoline G (**19**), and it was annotated based on MS/MS comparison using MetFrag. The peaks detected at 10.1 min with (*m*/*z* 380.1971) and at 15.0 (*m*/*z* 512.2402) corresponded to the molecular formulas C_22_H_16_N_4_O_2_, the same as fumitremorgin C (**2**), and C_27_H_33_N_3_O_7_, the same as verruculogen (**1**), respectively. Both compounds were identified based on an exact mass value and RT comparison with an in-house database. 

The peak detected at 10.2 min (*m*/*z* 357.1338) corresponded to the molecular formula C_21_H_25_N_3_O_3_, which can be associated with the fumiquinazoline K (**6**). The compound was identified based on an exact mass value and MS/MS with an in-house database and MetFrag.

The peaks detected at 11.9 min (*m*/*z* 584.2507) and 13.8 min (*m*/*z* 305.0200) corresponded to the molecular formulas C_31_H_37_NO_10,_ the same as pyripyropene A (**18**), and C_15_H_9_ClO_5_, the same as 2-chloroemodin (**13**), respectively, which were proposed based on MS/MS comparison using MetFrag. 

The peak detected at 18.4 min (*m*/*z* 281.2487) corresponded to the molecular formula C_18_H_32_O_2_ and was suggested to be conjugated linoleic acid (10E, 12Z) (**12**) using the GNPS database. The peak detected at 20.4 (*m*/*z* 429.3348) corresponded to the molecular formula C_28_H_44_O_3_, the same as ergosterol peroxide (**20**). The compound was identified based on MS/MS and RT comparison with an in-house database as well as a GNPS database.

All identified and annotated compounds were reported for various strains of *A. fumigatus* [26]. Verruculogen (**1**), [11] fumitremorgin C (**2**) [11], fumiquinazolines C (**3**), D (**4**) [12], and K (**6**) [13], tryptoquivalines J (**5**) [12] and F (**9**) [13], 6-methoxyspirotriprostatin B (**8**) [13], and spirotriprostatin A (**7**) [13] were previously isolated from this strain. Compounds **10**–**20** had not previously been isolated from this strain.

The most intensive peaks detected at 3.4 (*m*/*z* 227.0908), 8.2 (*m*/*z* 195.1003), 11.7 (*m*/*z* 380.1139), 12.7 (*m*/*z* 389.1924), 15.1 (*m*/*z* 256.2989), 15.8 (*m*/*z* 301.1407), 17.6 (*m*/*z* 377.2653), 18.4 (*m*/*z* 353.2654), and 20.4 (*m*/*z* 413.2657) min were not annotated using any available database and were not associated with common *A. fumigatus* metabolites.

### 3.3. Aspergillus fumigatus KMM 4631 and Penicilliun hispanicum KMM 4689 Co-Culture Metabolites

**Figure 4:**
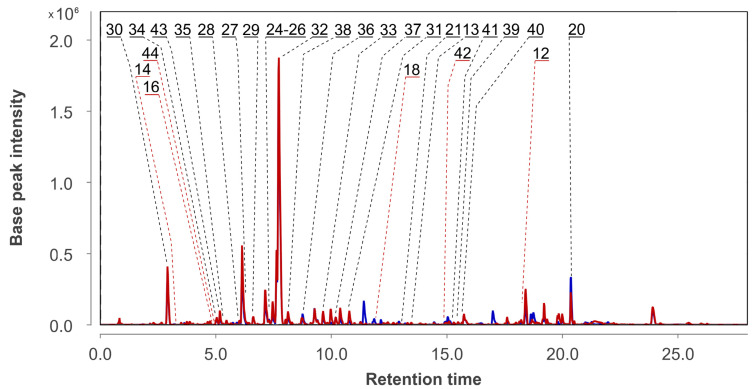
UHPLC MS chromatograms of Ph (blue) and AfPh (red) extracts.

The UHPLC MS chromatograms of extracts of the *Penicillium hispanicum* KMM 4689 monoculture (Ph) and the co-culture of *Aspergillus fumigatus* KMM 4631 and *Penicillium hispanicum* KMM 4689 (AfPh) are presented in Figure 4. 

**Figure 5:**
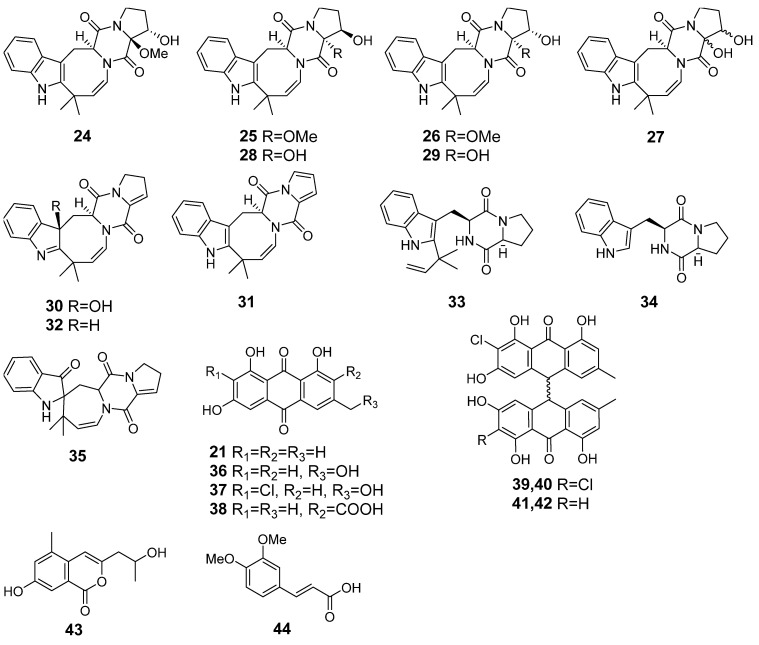
The compounds identified in Ph and AfPh extracts.

Emodin (**21**), desoxyisoaustamide alkaloids **24**–**32**, desoxybrevianamide E (**33**), brevianamide F (**34**), austamide (**35**), citreorosein (**36**), 2-chlorocitreorosein (**37**), endocrocin (**38**), nephrolaevigatins A–C (**39**–**41**), isochromene derivative **43**, and ergosterol peroxide (**20**), which were earlier identified in this fungal strain using the HPLC MS technique [17] were detected in Ph extract using an in-house database and GNPS (Figure 5, Table A1).

In AfPh, a total of 28 compounds were identified. They were compounds **12**–**16**, **18**, **20**, **21**, **24**–**41**, and **43**. Nephrolaevigatin D (**42**) and 3,4-dimethoxycinnamic acid (**44**) were found in the peaks at 15.0 and 4.9 min, respectively. These compounds were previously reported as metabolites of *Penicillium hispanicum* KMM 4689 [17]. Moreover, the peaks at 10.0 (*m*/*z* 329.1005) and 19.8 (*m*/*z* 443.3138) were detected only in AfPh and were not associated with any of the compounds in the used databases.

**Figure 6:**
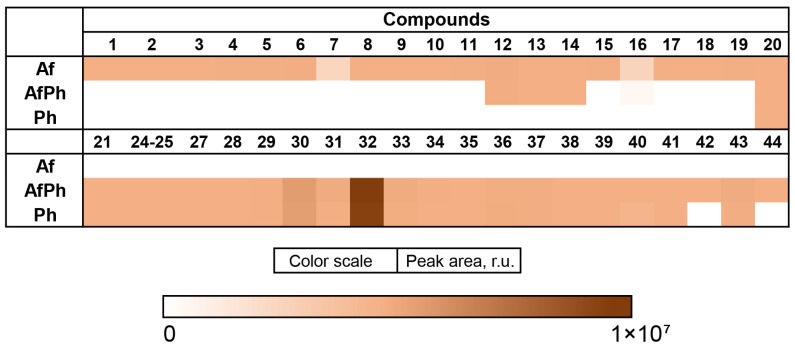
The heatmap of the content of compounds identified and annotated in the fungal extracts Af, Ph, and AfPh. Each cell presents a peak area in the HPLC MS chromatogram.

The content of the compounds identified as the peak areas in in the Af, Ph, and AfPh extracts, detected in the HPLC MS chromatogram, was visualized in a heatmap (Figure 6).

The content of 3β-hydroxydeoxyisoaustamide (**30**), (+)-deoxyisoaustamide (**32**), nephrolaevigatin B (**40**), and 7-hydroxy-3-(2-hydroxypropyl)-5-methylisochromen-1-one (**43**) was higher in the AfPh extract in comparison with the Af. Moreover, nephrolaevigatin D (**42**) as well as 3,4-dimethoxycinnamic acid (**44**) were detected in the AfPh extract only. Only the conjugated linoleic acid (10E, 12Z) (**12**), 2-chloroemodin (**13**), scytalone (**14**), and agroclavine (**16**) observed in the Af extract were also detected in the AfPh. Ergosterol peroxide (**20**) was identified in all extracts in equal amounts. 

### 3.4. Aspergillus fumigatus KMM 4631 and Amphichorda sp. KMM 4639 Co-Culture Metabolites

**Figure 7:**
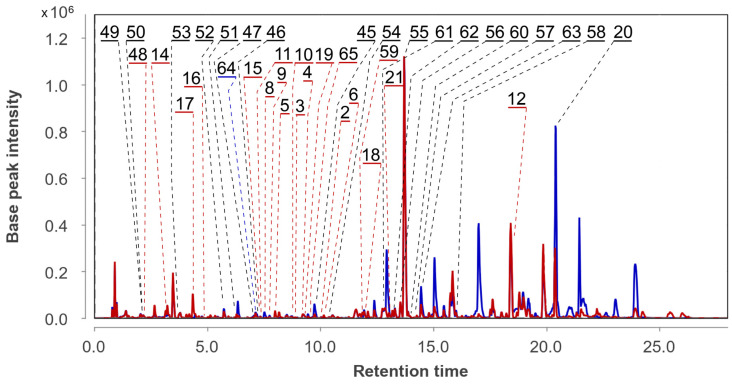
UHPLC MS chromatogram of As (blue) and AfAs (red) extracts.

The UHPLC MS chromatograms of extracts of the *Amphichorda* sp. KMM 4639 monoculture (As) and the co-culture of *Aspergillus fumigatus* KMM 4631 and *Amphichorda* sp. KMM 4639 (AfAs) are presented in Figure 7.

**Figure 8:**
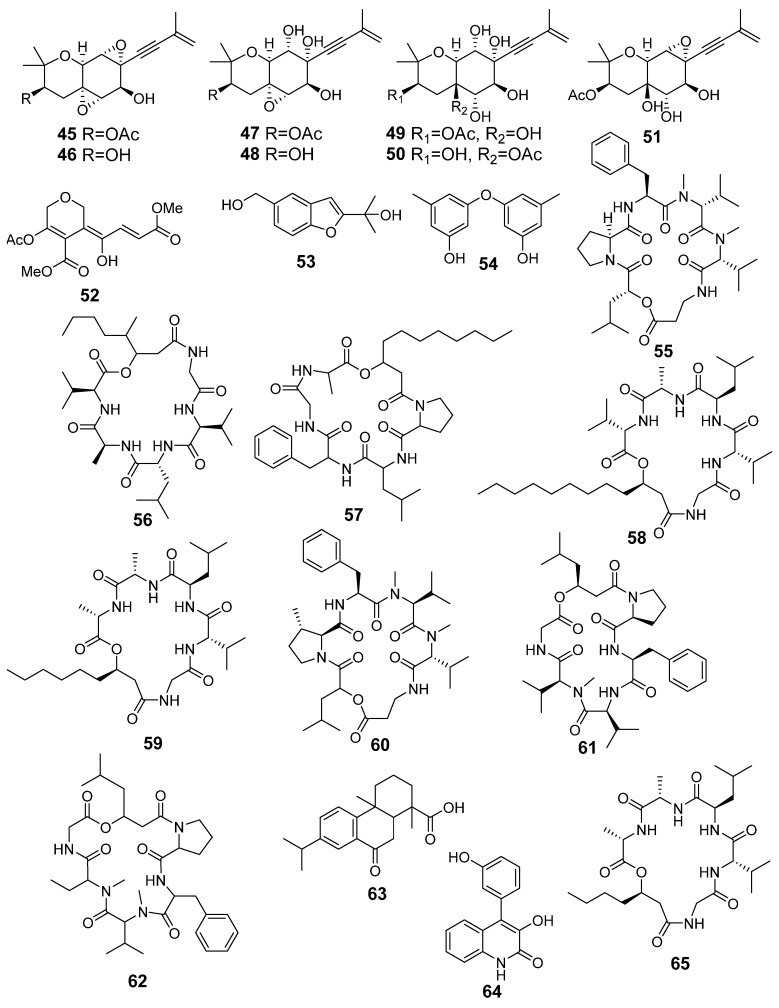
The compounds identified in As and AfAs extracts.

A total of 19 compounds (**20**, **45**–**47**, **49**–**58**, **60**–**64**) were identified and annotated in the As extract (Figure 8).

The peaks at 7.1 (*m*/*z* 353.1587), 7.2 (*m*/*z* 275.1279 [M−H_2_O+H]^+^) and 9.7 (*m*/*z* 335.1487) min corresponded to the molecular formulas C_18_H_24_O_7_, C_16_H_20_O_5_, and C_18_H_22_O_6_, which can be associated with the chromene derivatives oxirapentyns F (**47**), E (**46**), and B (**45**), respectively. This was additionally proven through a comparison of exact mass values and RT comparison with an in-house database.

The peaks detected at 2.9 min (*m*/*z* 371.1711) corresponded to the molecular formula C_18_H_26_O_8_, which may be associated with isomeric oxirapentyns H (**49**) and I (**50**). The RTs of the reference compounds were very close, and both of the compounds may be contained in this peak. The peaks at 5.7 (*m*/*z* 313.0910), 3.7 (*m*/*z* 207.1014), and 10.4 (*m*/*z* 231.1009) min corresponded to the molecular formulas C_14_H_16_O_8_, C_12_H_14_O_3_, and C_14_H_14_O_3_, the same as isariketide A (**52**), acremine S (**53**), and diorcin (**54**). The compounds were identified based on an exact mass value and RT comparison with an in-house database.

The peak at 6.2 (*m*/*z* 353.1587) min corresponded to the molecular formula C_18_H_24_O_7_, the same as oxirapentyn J (**51**). The compound was identified based on an exact mass value and RT comparison with an in-house database. The main peak was detected at 13.7 (*m*/*z* 656.4010) min in the HPLC MS chromatogram. It corresponds to the molecular formula C_35_H_53_N_5_O_7_, the same as isaridin E (**55**). This was suggested through the comparison of experimental MS/MS spectra with the GNPS database (MQScore 0.81). It should be noted that the earlier compounds **45**–**55** were reported as metabolites of the *Amphichorda* sp. KMM 4639 strain [27].

Many *Cordycipitaceae* fungi are known to produce various cyclodepsipeptides. The strain KMM 4639 is no exception. In addition to the main peptide isaridin E (**55**), we were able to detect other related metabolites. Unfortunately, their concentration in the extracts was low and we were unable to obtain MS/MS data to identify them more accurately, so the presence of these peptides was assumed based on the exact mass values.

Thus, the peaks detected at 12.8 (*m*/*z* 642.3870), 13.2 (*m*/*z* 642.3870), 13.5 (*m*/*z* 596.3997), 14.0 (*m*/*z* 670.4187), 14.1 (*m*/*z* 670.4187), and 16.0 (*m*/*z* 638.4495) min corresponded to the molecular formula C_34_H_51_N_5_O_7_, C_34_H_51_N_5_O_7_, C_30_H_53_N_5_O_7_, C_36_H_55_N_5_O_7_, C_36_H_55_N_5_O_7_, and C_33_H_59_N_5_O_7_, and were associated with desmethylisaridin E (**61**), isaridin F (**62**), isaridin B (**56**), psuedodestruxin C (**60**), isarfelin A (**57**), and isariin (**58**).

None of the peptides **56**–**62** have previously been isolated from the strain KMM 4639.

The peaks detected at 7.5 (*m*/*z* 254.0795) and 14.2 (*m*/*z* 315.1947) min corresponded to the molecular formulas C_15_H_11_NO_3_ and C_20_H_26_O_3_, and were associated with viridicatol (**64**) and 1,4a-dimethyl-9-oxo-7-propan-2-yl-3,4,10,10a-tetrahydro-2H-phenanthrene-1-carboxylic acid (**63**), based on MS/MS comparison with the GNPS database.

In the AfAs extract a total of 37 compounds were identified. They were compounds **2**–**6** and **8**–**20,** which were identified in the Af extract, and compounds **45**–**58** and **60**–**63**, identified in the As extract. Moreover, the peaks detected in the AfAs extract at 2.2 (*m*/*z* 311.1504), 11.8 (*m*/*z* 568.3692), and 9.7 (*m*/*z* 540.3395) min corresponded to the molecular formulas C_16_H_22_O_6_, C_28_H_49_N_5_O_7_, and C_26_H_45_N_5_O_7_, which can be associated with oxirapentyn G (**48**), isariin C (**59**), and D (**65**). These compounds were suggested based on exact mass values. No new unidentified intensive peaks were observed in the AfAs chromatogram.

**Figure 9:**
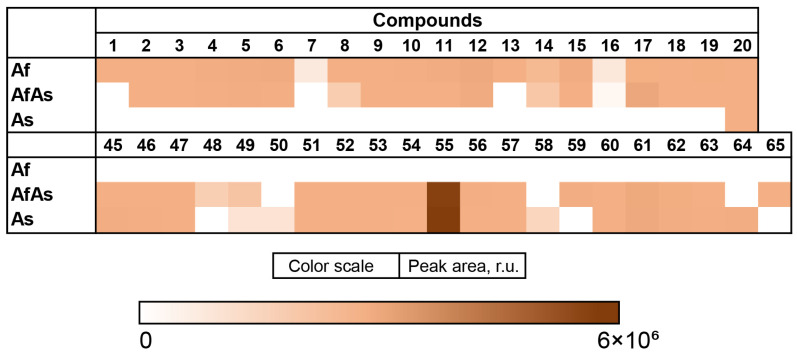
The heatmap of the content of compounds identified in the fungal extracts Af, As, and AfAs. Each cell presents a peak area in the HPLC MS chromatogram.

The content of the compounds identified as the peak areas in the Af, As, and AfAs extracts, detected in the HPLC MS chromatogram, was visualized in a heatmap (Figure 9).

The Af metabolites **2**–**6**, **9**–**12**, and **18** were observed in the AfAs extract in equal amounts compared with the Af, while compounds **1**, **7**, **13**, and **16** were not detected in the AfAs, and **8**, **14** were detected in a smaller amount. Only fumigaclavine A (**17**) was produced in the AfAs extract in higher amount than in the Af.

The As metabolites **45**–**47**, **51**–**57**, and **60**–**63** were observed in the AfAs extract in equal amounts in comparison with the As, while **50**, **58**, and **64** were not detected. The amount of oxirapentyn H (**49**) was higher in the AfAs than in the As extract, and oxirapentyn G (**48**), isariin C (**59**) and isariin D (**65**) were detected only in the AfAs extract.

### 3.5. Aspergillus fumigatus KMM 4631 and Penicillium sp. KMM 4672 Co-Culture Metabolites

**Figure 10:**
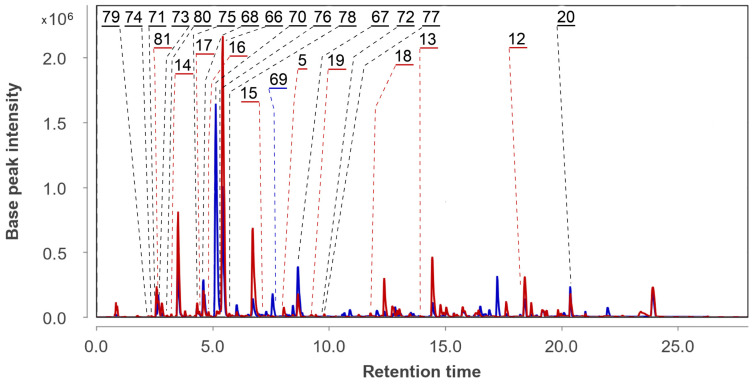
UHPLC MS chromatograms of Ps (blue) and AfPs (red) extracts.

The combined UHPLC MS chromatograms of the extracts of *Penicillium* sp. KMM 4672 monoculture (Ps) and the co-culture of *Aspergillus fumigatus* KMM 4631 and *Penicillium* sp. KMM 4672 (AfPs) are presented in Figure 10.

**Figure 11:**
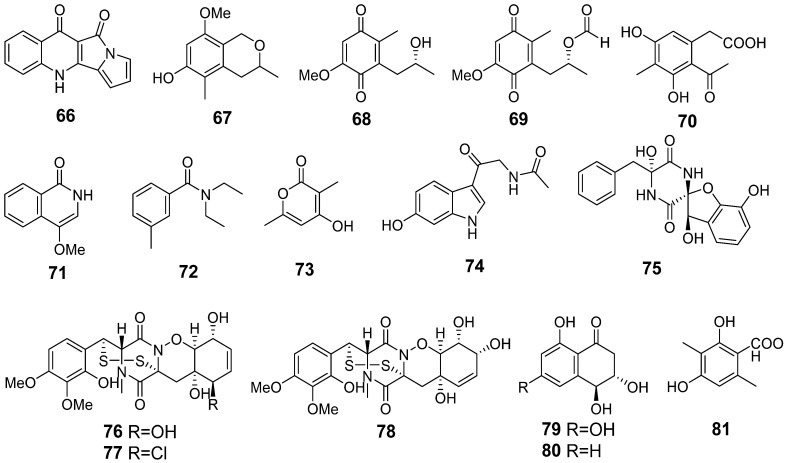
The compounds identified in Ps and AfPs extracts.

In total, 16 compounds were identified and annotated in the Ps extract (Figure 11, Table A1).

The main peak in the HPLC MS chromatograms was detected at 5.4 min. The base peak with *m*/*z* 237.0670 corresponded to the molecular formula C_14_H_8_N_2_O_2_, the same as quinolactacide (**66**) [28]. The compound was identified based on an exact mass value and RT comparison with an in-house database.

The peaks at 2.2 (*m*/*z* 211.0603) and 3.0 (*m*/*z* 195.0654) min corresponded to the molecular formulas C_10_H_10_O_5_ and C_10_H_10_O_4_, which can be associated with 4-hydroxyscytalone (**79**) and 4-hydroxy-6-dehydroxyscytalone (**80**) [29]. The compounds were identified based on exact mass values and RT comparisons with an in-house database.

The peaks at 2.4 (*m*/*z* 233.0925), 2.6 (*m*/*z* 176.0717), and 2.7 (*m*/*z* 141.0548) min corresponded to the molecular formulas C_12_H_12_N_2_O_3_, C_10_H_9_NO_2_, and C_7_H_8_O_3_, which can be associated with the melatonin derivative hydroxy-N-acetyl-β-oxotriptamine (**74**) [30], 4-methoxyisoquinolin-1(2H)-one (**71**), and 4-hydroxy-3,6-dimethyl-2-pyrone (**73**) [31]. The compounds were identified based on exact mass values and RT comparisons with an in-house database.

The peak at 4.4 min with *m*/*z* 339.0979 corresponded to the molecular formula C_18_H_16_N_2_O_6_ ([M−H_2_O+H]^+^ ion), the same as citriperazine D (**75**) [32]. This was additionally proven through a comparison of experimental MS/MS spectra and RT comparison with an in-house database.

The peaks detected at 4.6 (*m*/*z* 193.0856) and 7.5 (*m*/*z* 239.0913) min corresponded to the molecular formulas C_11_H_14_O_4_ ([M−H_2_O+H]^+^) and C_12_H_14_O_5_, the same as anserinone B (**68**) and formylanserinone B (**69**) [33], respectively. The compounds were identified based on exact mass values and RT comparisons with an in-house database.

The peaks at 5.3 (*m*/*z* 207.0651, [M−H_2_O+H]^+^), 8.7 (*m*/*z* 209.1175), and 9.7 (*m*/*z* 192.1390) min corresponded to the molecular formulas C_11_H_12_O_5_, C_12_H_16_O_3_, and C_12_H_17_NO, the same as 6-methylcurvulinic acid (**70**) [34], 3,5-dimethyl-8-methoxy-3,4-dihydro-1H-isochromene-6-ol (**67**) [35], and N,N-diethyl-3-methylbenzamide (**72**) [36]. The compounds were identified based on exact mass values and RT comparisons with an in-house database.

The peaks at 5.4 (*m*/*z* 513.0988) and 8.1 (*m*/*z* 531.0662) min corresponded to the molecular formulas C_21_H_24_N_2_O_9_S_2_ and C_21_H_23_ClN_2_O_8_S_2_, the same as pretrichodermamide C (**76**) and N-methylpretrichodermamide B (**77**) [16,37], respectively. The compounds were identified based on exact mass values and RT comparisons with an in-house database. The peak at 5.9 min (*m*/*z* 513.0988) corresponded to the molecular formula C_21_H_24_N_2_O_9_S_2_, the same as pretrichodermamide D (**78**). This was additionally proven through a comparison of experimental MS/MS spectra and RT comparison with an in-house database. Moreover, ergosterol peroxide (**20**) was detected in the Ps extract. 

The earlier compounds **66**–**82** were reported as metabolites of *Penicillium* sp. KMM 4672 [16,29,30,32,36].

In the AfPs extract the compounds **5**, **12**–**20**, **66**–**68**, and **70**–**80** were identified. Moreover, the peak at 2.6 min with *m*/*z* 183.0659 in the HPLC MS chromatogram of the AfPs extract corresponded to the molecular formula C_9_H_10_O_4_, which can be associated with 3-methylorsellinic acid (**81**). The compound was suggested based on an exact mass value. Compound **81** was also previously isolated from this fungal strain [30].

In addition, the intensive peaks at 12.7 (*m*/*z* 214.2518), 16.3 (*m*/*z* 498.3788), and 23.4 (*m*/*z* 791.5300) were detected both in AfPs and Ps extracts, but their intensities in the AfPs extract were more than 20 times higher than in the Ps. 

**Figure 12:**
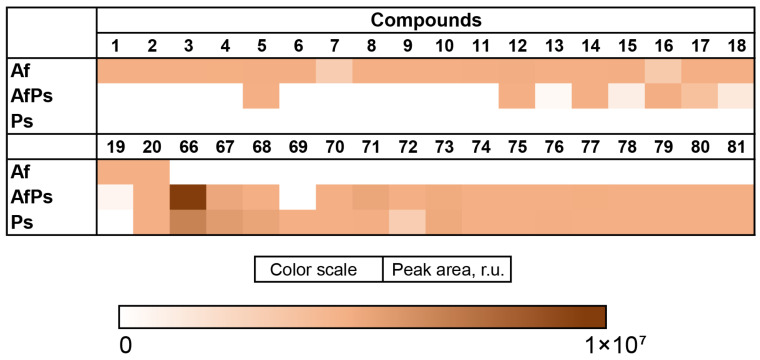
The heatmap of the content of compounds identified in the fungal extracts Af, Ps, and AfPs. Each cell presents a peak area in the HPLC MS chromatogram.

The content of the compounds identified as the peak areas in the Af, Ps, and AfPs extracts, detected in the HPLC MS chromatogram, is visualized in a heatmap (Figure 12).

The Af metabolites **5**, **12**, and **14** were detected in the AfPs extract in equal amounts compared with the monoculture. The content of **15** and **17**–**19** was less than in the Af extract and **1**–**4**, **6**–**11**, and **13** were not detected in the AfPs. Only the amount of agroclavine (**16**) was higher in the AfPs extract than in the Af.

The Ps metabolites **70** and **73**–**81** were detected in the AfPs extract in equal amounts, and only formylanserinone B (**69**) was not detected in the AfPs extract. The amounts of **67** and **68** were less in the AfPs extract than in the Ps, while the content of quinolactacide (**66**), 4-methoxyisoquinolin-1(2H)-one (**71**), and N,N-diethyl-3-methylbenzamide (**72**) was higher in the AfPs extract than in the Ps. 

### 3.6. Aspergillus fumigatus KMM 4631 and Asteromyces cruciatus KMM 4696 Co-Culture Metabolites

**Figure 13:**
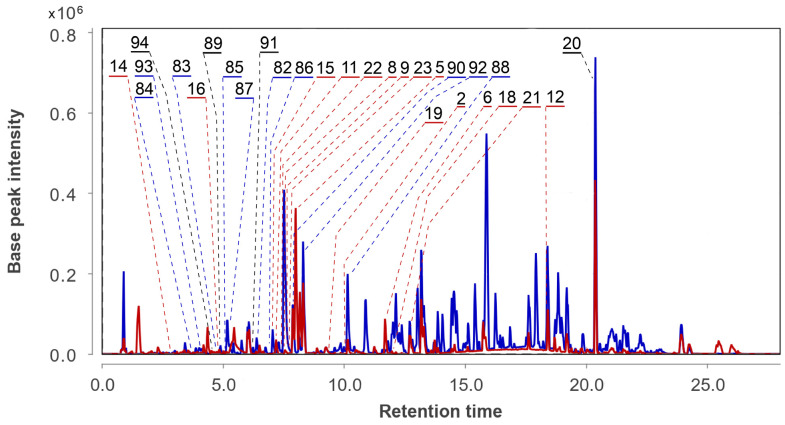
UHPLC MS chromatogram of Ac (blue) and AfAc (red) extracts.

The UHPLC MS chromatograms of the *Asteromyces cruciatus* KMM 4696 monoculture (Ac) and the co-culture of *Aspergillus fumigatus* KMM 4631 and *Asteromyces cruciatus* KMM 4696 (AfAc) extracts are presented in Figure 13.

**Figure 14:**
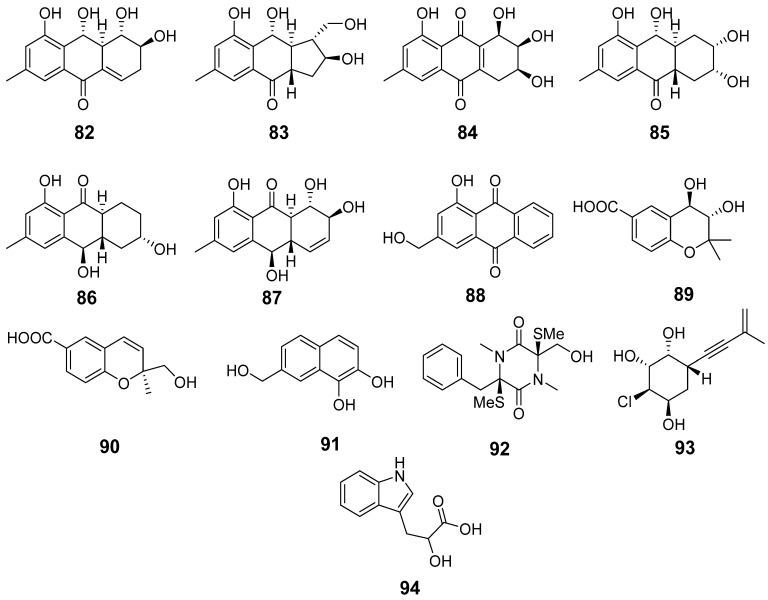
The compounds identified in Ac and AfAc extracts.

In total, 13 compounds were identified and annotated in the Ac extract (Figure 14, Table A1).

The peak at 4.6 min with *m*/*z* 206.0810 corresponded to the molecular formula C_11_H_11_NO_3_. It was suggested to be indolelactic acid (**94**) [38] based on MS/MS comparison using the GNPS database.

The peak at 4.9 min with *m*/*z* 239.0895 corresponded to the molecular formula C_12_H_14_O_5_, the same as trans-3,4-dihydroxy-3,4-dihydroanofinic acid (**89**) [39]. This was proven through a comparison of experimental MS/MS spectra and RT comparison with an in-house database.

The peak at 6.2 min with *m*/*z* 191.0707 corresponded to the molecular formula C_11_H_10_O_3_, the same as 7-hydroxymethyl-1,2-naphthalenediol (**91**) [40]. The compound was identified based on an exact mass value and RT comparison with an in-house database. 

The peaks detected at 6.4 (*m*/*z* 277.1063) and 5.2 (*m*/*z* 277.1062) min corresponded to the molecular formula C_15_H_16_O_5_, which can be associated with the isomeric anthraquinone derivatives acruciquinone A (**82**) [41] and rubrumol (**87**) [42]. The comparison of experimental MS/MS spectra and an RT comparison with the values obtained for reference compounds showed that the RTs of 6.4 and 5.2 min correspond to acruciquinone A (**82**) and rubrumol (**87**), respectively.

The peaks detected at 4.6 (*m*/*z* 279.1226) and 4.9 (*m*/*z* 279.1226) min corresponded to the molecular formula C_15_H_18_O_5_, which can be associated with the isomeric anthraquinone derivatives acruciquinone C (**83**) and coniothyrinone D (**85**). The comparison of experimental MS/MS spectra and an RT comparison with the value obtained for the reference compound showed that a RT of 4.9 min corresponds to coniothyrinone D (**85**) [43]. The peak detected at 4.6 min with the same *m*/*z* very likely corresponds to acruciquinone C (**83**) [41], but this peak was detected in extracts only in trace quantities, so we have no MS/MS for exact identification.

The peak detected at 3.8 min with *m*/*z* 291.0853 corresponded to the molecular formula C_15_H_14_O_6_, the same as pleosporon (**84**) [44]. The compound was identified based on an exact mass value and RT comparison with an in-house database.

The peaks detected at 7.0 (*m*/*z* 263.1272) and 10.1 (*m*/*z* 255.0652) corresponded to the molecular formula C_15_H_18_O_4_ and C_15_H_10_O_4_, which can be associated with coniothyrinone B (**86**) [43] and 9,10-anthracenedione (**88**). This was additionally proven through a comparison of experimental MS/MS spectra and RT comparison with an in-house database.

The peak detected at 7.8 min with *m*/*z* 221.0819 corresponded to the molecular formula C_12_H_12_O_4_, the same as quadricinctapyran A (**90**) [45]. The compound was identified based on an exact mass value and RT comparison with an in-house database.

The peaks detected at 8.3 (*m*/*z* 355.1160) and 4.1 (*m*/*z* 231.0777) corresponded to the molecular formula C_16_H_22_N_2_O_3_S_2_ and C_11_H_15_ClO_3_, which can be associated with gliovictin (**92**) [46] and acrucipentyn A (**93**) [18]. This was additionally proven through a comparison of experimental MS/MS spectra and an RT comparison with an in-house database.

In the AfAc extract the compounds **2**, **5**, **6**, **8**, **9**, **11**–**16**, **18**–**21**, **89**, **91**, and **94** were identified. Moreover, the peaks at 7.3 (*m*/*z* 426.2035, C_23_H_27_N_3_O_5_) and 7.4 (*m*/*z* 357.0936, C_15_H_20_N_2_O_4_S) min were associated with cyclotryprostatin B (**22**) and bisdethiobic(methylthio)gliotoxin (**23**). They were previously isolated from *Aspergillus fumigatus* KMM 4631 [11,12], but in the present study both these compounds were identified in the AfAc extract only.

In addition, the intensive peaks at 5.4 (*m*/*z* 470.1496) and 5.5 (*m*/*z* 877.3782) min were detected only in the AfAc chromatogram, while the intensive peaks found at 2.3 (*m*/*z* 113.0598), 4.8 (*m*/*z* 183.0649), and 13.7 (*m*/*z* 233.1154) min were 10–20 times more intensive in the AfAc chromatogram than in the Ac. 

**Figure 15:**
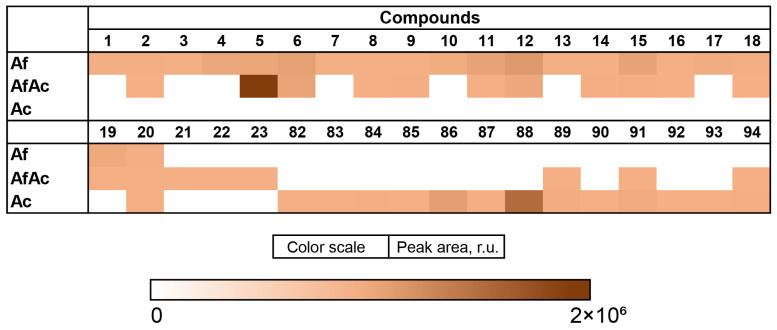
The heatmap of the content of compounds identified in the fungal extracts Af, Ac, and AfAc. Each cell presents a peak area in the HPLC MS chromatogram.

The content of the compounds identified as the peak areas in the Af, Pc, and AfAc extracts, detected in the HPLC MS chromatogram, is visualized in a heatmap (Figure 15).

The Af metabolites **2**, **8**, **9**, **14**, **16**, and **18** were detected in the AfAc extract in equal amounts compared with the Af. The amount of **6**, **11**, **12**, **15**, and **19** was less in the AfAc extract than in the Af, while **1**, **3**, **4**, **7**, **10**, **13**, and **17** were not detected in the AfAc extract. It was surprising that the content of tryptoquivaline J (**5**) was significantly higher in the AfAc extract than in the Af.

The Ac metabolites **82**–**88**, **90**, **92**, and **93** were not detected in the AfAc extract and only **89**, **91,** and **94** were observed in the AfAc extract in equal amounts in comparison with the monoculture. Emodin (**21**), cyclotryprostatin B (**22**), and bisdethiobis(methylthio)gliotoxin (**23**) were detected only in the AfAc extract.

### 3.7. PCA Analysis of the HPLC MS Chromatograms of Fungal Extracts

**Figure 16:**
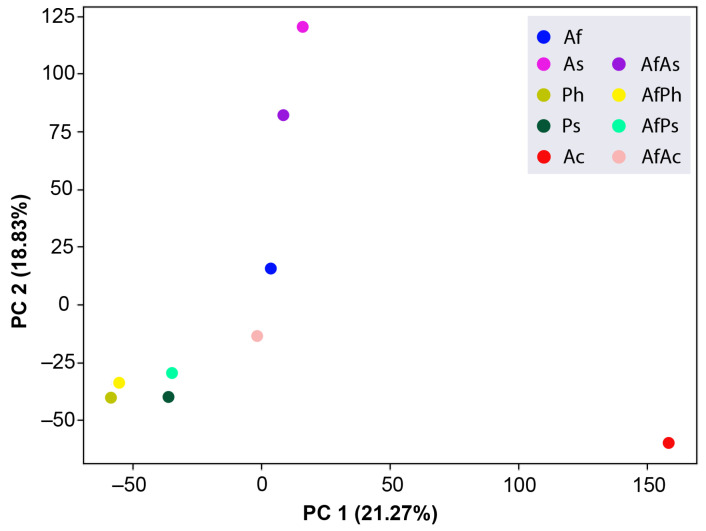
The principal component analysis (PCA) of all studied extracts.

Principal component analysis (PCA) was utilized to differentiate between the extracts analyzed via UHPLC MS. The PCA model revealed an optimal number of principal components (PCs) equal to two, so two PCs (PC1 and PC2) were chosen to describe ≈40% of the variation in the samples. The first principal component describes approximately 21% of the variance, and ≈19% of the variation is described by the second principal component (Figure 16).

The results of the PCA analysis showed that the extracts of both *Penicillium* strains Ph and Ps are very close to their co-cultures with *A. fumigatus,* AfPh and AfPs, in terms of the two principal components. The AfAc extract is the closest to the Af in PC1, and the Ac extracts is the most different from other extracts in PC1. The AfAs extract is a bit closer to the As than to the Af extract in PC2.

**Figure 17:**
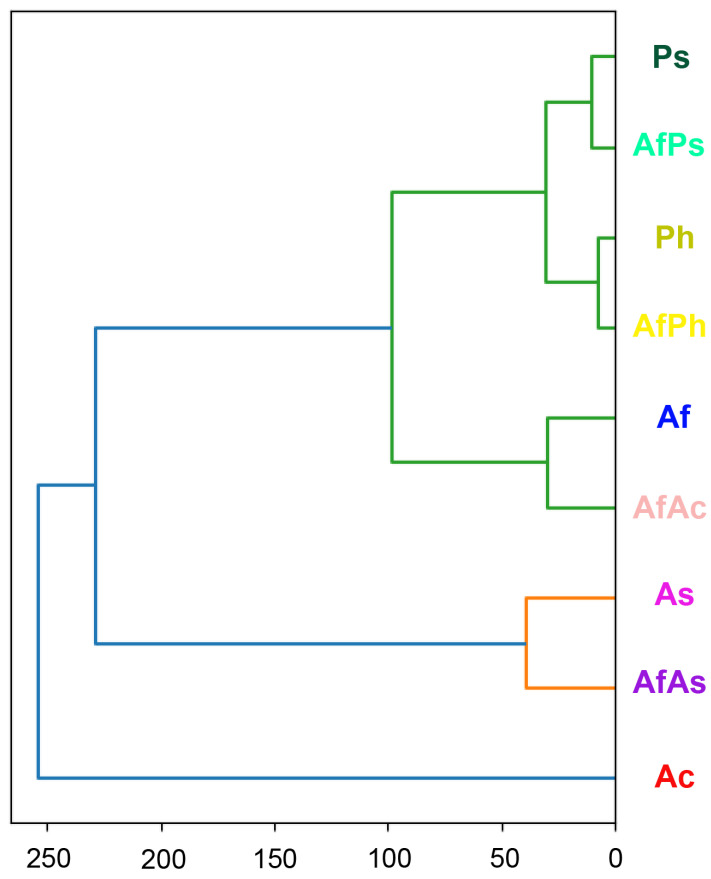
Dendrogram of the clustering of all investigated extracts. Different colors indicate different clusters.

The studied extracts were divided into three clusters based on the PCA of the UHPLC MS data, which was visualized in a dendrogram (Figure 17). 

The dendrogram confirms the relationships between the extracts visualized on the PCA plot.

### 3.8. Influence of Co-Cultivation on the Biological Activity of Fungal Extracts

The joint cultivation of different fungal strains induces cell wall integrity (CWI) stress, the limitation of nutrition and O_2_, and oxidative stress, which results in the activation of various adaptive strategies including the production of direct or indirect antioxidants or secondary metabolites targeting the suppression of a competitor’s viability. Oxidative stress induces the production of reactive oxygen and nitrogen species which can be scavenged by direct antioxidants. Urease (urea amino hydrolase), which hydrolyzes urea to ammonia and carbamate, is an important virulent factor for some fungi like *Helicobacter pylori* and other bacteria [47]. The inhibition of urease activity can result in a decrease of urease-producing strains’ virulence, so, it may be one of the defense strategies in joint fungus–fungus cultivation [48]. Also, secondary metabolites with cytotoxic properties are produced during fungal co-cultivation [49,50].

**Figure 18:**
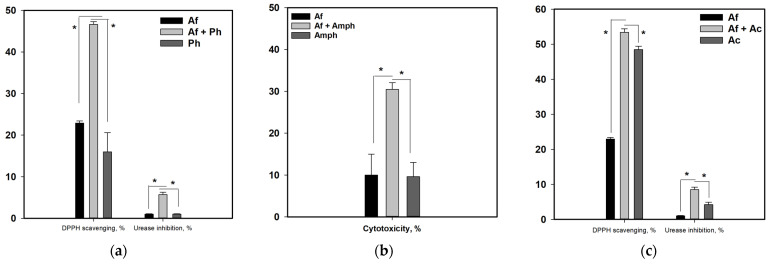
Biological activity of fungal mono- and co-culture extracts. (**a**) DPPH radical scavenging and urease inhibition activity of Af, AfPh, and Ph extracts; (**b**) cytotoxic activity toward human hepatocarcinoma HepG2 cells of Af, AfAs, and As extracts; (**c**) DPPH radical scavenging and urease inhibition activity of Af, AfAc, and Ac extracts. All experiments were carried out in triplicate. * indicates the significant differences with *p* ≤ 0.05.

Thus, the effect of fungal mono- and co-culture extracts on DPPH radical scavenging, urease activity, and human hepatocarcinoma HepG2 cell viability was investigated, and some data are presented in Figure 18. 

The DPPH radical scavenging activity of the Af extract was 22.9%. The AfPh extract scavenged 46.6% of DPPH radicals, while the Ph extract scavenged only 16.0% of DPPH radicals (Figure 18a).

The extract of AfAc scavenged 53.4% of DPPH radicals, while the Ac extract decreased the quantity of DPPH radicals by only 48.5% (Figure 18c). 

The extract of Af inhibited the activity of urease only by almost 1% while the AfPh and AfAc extracts inhibited urease activity by 5.7% and 8.5%, respectively.

The extract of Af decreased HepG2 cell viability by 10.0% while the AfAs extract was more toxic and decreased HepG2 cell viability by 30.5% (Figure 18b).

The cytotoxicity, as well as the DPPH radical scavenging and urease inhibition activities, of the AfPs extract did not differ from those of the Af extract.

## 4. Discussion

In the present work, the metabolite profiles of marine *A. fumigatus* strain KMM 4631 and other marine fungi *Amphichorda* sp. KMM 4639, *Penicillium* sp. KMM 4672, and *Asteromyces cruciatus* KMM 4696 were investigated for the first time. This helps us to study the metabolite profiles of co-cultures of *A. fumigatus* with *Amphichorda* sp., *Penicillium* sp., *Asteromyces cruciatus*, and *Penicillium hispanicum* KMM 4689.

In total, 20 known compounds were analyzed in this marine *A. fumigatus* strain and some of them had not yet been isolated from this fungus. A number of secondary metabolites such as gliotoxin, fumagillin, fumigaclavines, fumitremorgins, and fumiquinazolines have previously been described as the main virulence factors of *A. fumigatus* [51]. The strains isolated from environmental sources may display a decreased production of these metabolites [10], which can result in the higher stress sensitivity of non-pathogenic strains. It has been reported that non-pathogenic *A. fumigatus* Af293, isolated from decaying organic and plant matter, exhibits moderate tolerance to the cell wall stress caused by Congo Red and Calcofluor White, and moderate sensitivity to the oxidative stress induced by menadione and H_2_O_2_ [52]. Fusidane-type triterpenoid helvolic acid [53], which aids in the colonization of mammalian cells by *A. fumigatus*, decreasing the beat frequency of the ciliated respiratory epithelium, as does as fumagillin [51], was not identified in the marine isolate of *A. fumigatus* studied in the present investigation. On the other hand, an unusual nordammarane triterpenoid was isolated from this marine *A. fumigatus* strain [13], and its biological role may be related to adaptation to marine environments like the anti-inflammatory nordammarane triterpenoid decurrencyclic A [54]. Moreover, nine intensive peaks in the LC-MS chromatogram of the *A. fumigatus* extract were not annotated with any common metabolites of *A. fumigatus,* and this 15-year-ago-investigated strain may have become “second born”, via the use of actual techniques for the isolation and identification of minor natural compounds.

The joint cultivation of fungi mimics natural communities and can have significant effects on the biosynthesis of secondary metabolites. In addition to resource limitations, physical contact during co-culture induces cell wall integrity (CWI) stress [55], both of which cause changes in the production of compounds that act as second messengers or directly (e.g., direct antioxidants). 

The metabolite profiles of co-cultures of *A. fumigatus* with two *Penicillium* strains, KMM 4672 and KMM 4689, were very close to the profiles of the monocultures of those *Penicillium* fungi. Nevertheless, their co-cultivation with *P. hispanicum* KMM 4689 resulted in the detection of two new unidentified compounds. Furthermore, three previously unidentified compounds were detected in significantly higher amounts in the co-culture of *A. fumigatus* and *Penicillium* sp. KMM 4672. The mixed cultivation of *A. fumigatus* and *P. hispanicum* resulted in the increased DPPH radical scavenging and urease inhibition activities of the co-culture extract. However, these activities have not been previously reported for the known metabolites identified in the *A. fumigatus* and *Penicillium hispanicum* co-culture.

The metabolite profile of the co-culture of *A. fumigatus* with *Amphichorda* sp. was like that of the *Amphichorda* sp. monoculture. Three metabolites that were previously reported for *Amphichorda* sp. were detected only in the co-culture extract, but no new unidentified compounds were observed when *A. fumigatus* was cultivated with *Amphichorda* sp. A significant increase in cytotoxic activity was observed for an extract of this co-culture and it was accompanied by the detection of oxirapentyn G, isariin C, and isariin D in this extract. Isariin C has been reported as insecticidal against *Galleria mellonella* [56], as has isariin D [57], but cytotoxic activity has not yet been published for these depsipeptides. The cytotoxic activity of oxirapentyn G has also not been reported. Thus, the extract of the *A. fumigatus* and *Amphichorda* sp. joint culture may contain unidentified cytotoxic compounds.

The metabolite profile of the co-culture of *A. fumigatus* with *Asteromyces cruciatus* is more distinct from those of each monoculture. Three known fungal metabolites were detected only in this co-culture and three unidentified compounds were observed in significantly higher amounts compared with the monocultures. In addition, the co-cultivation of *A. fumigatus* with *Asteromyces cruciatus* resulted in the production of two new unidentified compounds in significant amounts.

As noted above, gliotoxin was not identified in the extract of *A. fumigatus* KMM 4631, and its derivative bisdethiobis(methylthio)gliotoxin was observed only in the co-culture of *A. fumigatus* with *Asteromyces cruciatus*. Gliotoxin via the action of gliotoxin bis-thiomethyltransferase (GtmA) can transform into *bis*dethio*bis*(methylthio)gliotoxin [58]. This mechanism of self-protection against toxicity induced by cycling between the oxidized and reduced dithiol form and the generation of reactive oxygen species was reported for *A. fumigatus* [59], but could also be true for other fungi. Thus, gliotoxin, dehydroxy*bis*dethio*bis*(methylthio)gliotoxin, and *bis*dethio*bis*(methylthio)gliotoxin were isolated also from a marine *Pseudallescheria* fungus [60]. It was previously reported that the production of gliotoxin was changed in response to contact with other microorganisms during co-cultivation [61]. So, gliotoxin plays a double role, i.e., it can both regulate the growth of a competitor fungus [62] and protect the producing fungus, as an antioxidant [63] during CWI stress. In the present work, the derivative of gliotoxin was detected only in the co-culture of *A. fumigatus* with *Asteromyces cruciatus.* At this stage of our investigation it is difficult to say which of them is responsible for the production of this gliotoxin derivative—it could be either the result of *A. fumigatus’* self-protection or the result of a defense strategy of *Asteromyces cruciatus*.

The co-culture extract exhibited a significant increase in both DPPH radical scavenging and urease inhibition activities. Previously, urease inhibition activity has been reported for trans-3,4-dihydroxy-3,4-dihydroanofinic acid and 7-hydroxymethyl-1,2-naphthalenediol from *Asteromyces cruciatus* [41], but the detection of only these two metabolites in the co-culture of *A. fumigatus* and *Asteromyces cruciatus* cannot explain the increase in the urease inhibition activity of the joint culture extract. Furthermore, the observed increase in DPPH radical scavenging activity of the co-culture extract raises further questions.

These results confirm that competition between fungi in the communities is carried out, among other things, through chemical communication. Exolites that are capable of scavenging active radicals play a protective role in the interactions between community members. An increase in the radical scavenging activity of extracts directly indicates the activation of the defense system of one or both fungi in their joint culture [64]. The urease enzyme is important in modifying microenvironmental conditions and is utilized by a wide range of prokaryotes and microeukaryotes, including fungi [65]. The inhibition of urease activity can reduce the virulence and the viability of its producers. Therefore, the effectiveness of a well-known drug targeted at *Helicobacter pylori* is based on the suppression of urease activity [66]. The enhanced ability to inhibit urease activity found in extracts of *A. fumigatus* and *Asteromyces cruciatus,* as well as the *A. fumigatus* and *P. hispanicum* co-cultures, indicates that fungi use a tactic involving affecting another member of the community. An increase in the cytotoxic activity of the extract of another joint culture also confirms the use of an aggressive strategy by fungi in the community [67]. 

## 5. Conclusions

Thus, the joint cultivation of the marine non-pathogenic strain *Aspergillus fumigatus* KMM 4631 with two marine *Penicillium* fungi may result in the production of some novel compounds. The co-cultivation of *Aspergillus fumigatus* with *Asteromyces cruciatus* KMM 4696 resulted in more pronounced changes in secondary metabolite biosynthesis and may be the most promising for the isolation of unknown compounds or for enhancing the production of known bioactive metabolites. 

## Figures and Tables

**Table 1:** The strains of the species used in multi-locus phylogenetic analysis and their GenBank accession numbers.

Species	Strain Number	GenBank Accession Number			
		ITS	*BenA*	*CaM*	*RPB2*
*Aspergillus takakii*	CBS 137454^T^	MN431378	AB787221	AB787566	MN969097
*Aspergillus laciniosus*	CBS 117721^T^	AB299413	AY870756	AY870716	MN969080
*Aspergillus spinosus*	CBS 483.65^T^	EF669988	EF669844	EF669914	EF669775
*Aspergillus fumisynnematus*	CBS 141446^T^	AB250779	AB248076	AB259968	MN969073
*Aspergillus lentulus*	CBS 117885^T^	EF669969	EF669825	EF669895	EF669756
*Aspergillus fumigatiaffinis*	CBS 117186^T^	MN431367	DQ094885	DQ094891	MN969072
*Aspergillus oerlinghausenensis*	CBS 139183^T^	KT359601	KT359603	KT359605	MN969162
*Aspergillus fumigatus*	CBS 133.61^T^	EF669931	EF669791	EF669860	EF669719
***Aspergillus fumigatus***	**KMM 4631**	**OR578448**	**OQ466614**	**OR600992**	**OR600993**
*Aspergillus fischeri*	CBS 544.65^T^	EF669936	EF669796	EF669865	EF669724
*Aspergillus novofumigatus*	CBS 117520^T^	MN431372	DQ094886	DQ094893	MN969083
*Talaromyces marneffei*	CBS 388.87^T^	JN899344	JX091389	KF741958	KM023283

^T^—ex-type strain

**Table 2:** Amounts of the extracts of the fungal cultures.

Fungal Culture	Sample Code	Mass of Crude Extract, mg
*Aspergillus fumigatus* KMM 4631	Af	21.1
*Aspergillus fumigatus* KMM 4631+ *Amphichorda* sp. KMM 4639	AfAs	14.5
*Amphichorda* sp. KMM 4639	As	12.7
*Aspergillus fumigatus* KMM 4631+ *Penicillium* sp. KMM 4672	AfPs	87.0
*Penicillium* sp. KMM 4672	Ps	102.1
*Aspergillus fumigatus* KMM 4631+ *Penicillium hispanicum* KMM 4689	AfPh	365.0
*Penicillium hispanicum* KMM 4689	Ph	233.1
*Aspergillus fumigatus* KMM 4631+ *Asteromyces cruciatus* KMM 4696	AfAc	39.5
*Asteromyces cruciatus* KMM 4696	Ac	56.3

## Data Availability

The original data presented in the study are included in the article/Supplementary Materials; further inquiries can be directed to the corresponding author.

## Supplementary Materials

The following supporting information can be downloaded at: https://www.mdpi.com/article/10.3390/metabo13111138/s1, Figures S1–S9: UPLC MS chromatograms of the investigated extracts, Figures S10-S40: MS/MS fragmentation of compounds **3**–**6**, **9**–**21**, **54**–**56**, **58**, **60**–**64**, **75**–**78**, **86**, **88**, **89**, **94**, Table S1: Experimental UPLC MS data for reference compounds.

## Appendix A

**Table A1:** The secondary metabolites identified in *Aspergillus fumigatus* KMM 4631 (Af), *Penicillium hispanicum* KMM 4689 (Ph), *Amphichorda* sp. KMM 4639 (As), *Penicillium* sp. KMM 4672 (Ps), and *Asteromyces cruciatus* KMM 4696 (Ac) monoculture extracts and the *Aspergillus fumigatus* co-cultures with other investigated fungi.

N		Name	Structure	RT	Exact Mass (Measured)	Exact Mass (Calcd)	Δ, ppm	Score
1	Af	verruculogen		15.0	512.2414 [M+H]^+^	512.2391	4.5	
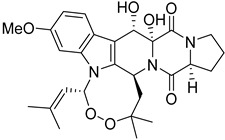
2	Af AfAs AfAc	fumitremorgin C		10.1	380.1971 [M+H]^+^	380.1969	0.5	
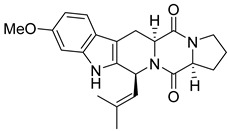
3	Af AfAs	fumiquinazoline C		9.0	444.1671 [M+H]^+^	444.1666	1.1	
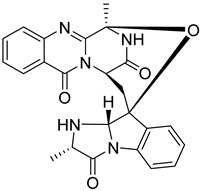
4	Af AfAs	fumiquinazoline D		9.2	444.1671 [M+H]^+^	444.1666	1.1	0.95 ^a^
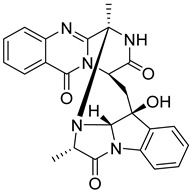
5	Af AfAs AfPs AfAc	tryptoquivaline J		8.0	403.1396 [M+H]^+^	403.1401	−1.2	
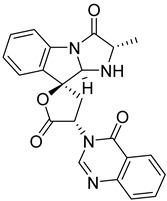
6	Af AfAs AfAc	fumiquinazoline K		10.2	357.1338 [M+H]^+^	357.1346	−2.2	
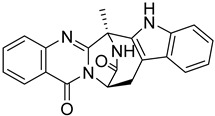
7	Af	spirotriprostatin A		10.4	396.1920 [M+H]^+^	396.1918	0.5	
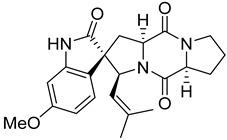
8	Af AfAs AfAc	6- methoxyspirotriprostatin B		7.6	394.1754 [M+H]^+^	394.1761	−1.8	
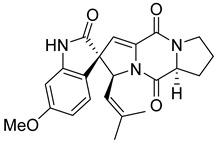
9	Af AfAs AfAc	tryptoquivaline F		7.8	403.1396 [M+H]^+^	403.1401	−1.2	
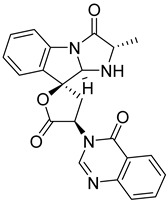
10	Af AfAs	fumiquinazoline F		8.7	359.1494 [M+H]^+^	359.1503	−2.5	0.83 ^a^
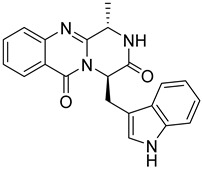
11	Af AfAs AfAc	pseurotin A		7.1	432.1652 [M+H]^+^	432.1653	−0.2	0.76 ^a^, 0.95 ^b^
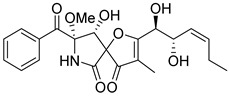
12	Af AfPh AfAs AfPs AfAc	conjugated linoleic acid (10E, 12Z)		18.4	281.2487	281.2475	4.3	0.74 ^a^
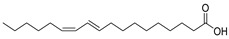
13	Af Ph AfPh AfPs	2-chloroemodin		13.8	305.0200	305.0211	−3.6	1.00 ^b^
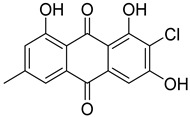
14	Af AfPh AfAs AfPs AfAc	scytalone		3.2	195.0649	195.0652	−1.5	0.84 ^b^
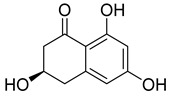
15	Af AfAs AfPs AfAc	pseurotin D		7.2	432.1650	432.1653	−0.7	1.00 ^b^
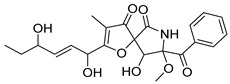
16	Af AfPh AfAs AfPs AfAc	agroclavine		4.8	239.1519	239.1543	−10.4	1.00 ^b^
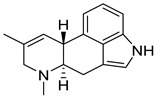
17	Af AfAs AfPs	fumigaclavine A		4.4	299.1749	299.1754	−1.7	1.00 ^b^
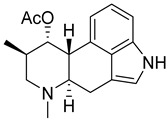
18	Af AfAs AfPs AfAc	pyripyropene A		11.9	584.2507	584.2490	2.9	1.00 ^b^
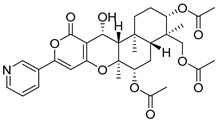
19	Af AfAs AfPs AfAc	fumiquinazoline G		9.4	359.1480	359.1503	−6.4	0.80 ^b^
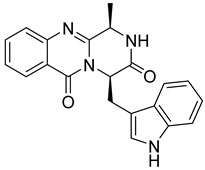
20	Af, Ph As, Ps Ac AfPh AfAs AfPs AfAc	ergosterol peroxide		20.4	429.3348	429.3363	−3.5	0.85 ^a^
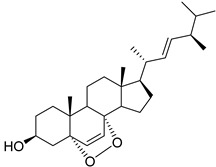
24	Ph AfPh	16α-hydroxy-17β-methoxy-deoxydihydroisoaustamide		7.3	396.1921 [M+H]^+^	396.1918	0.8	
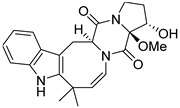
25	Ph AfPh	16β-hydroxy-17α-methoxy-deoxydihydroisoaustamide		7.3	396.1921 [M+H]^+^	396.1918	0.8	
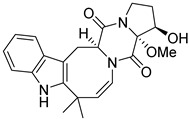
26	Ph AfPh	16α-hydroxy-17α-methoxy-deoxydihydroisoaustamide		7.3	396.1921 [M+H]^+^	396.1918	0.8	
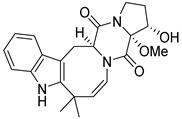
27	Ph AfPh	16,17-dihydroxy-deoxydihydroisoaustamide		6.2	382.1748 [M+H]^+^	382.1761	−3.4	
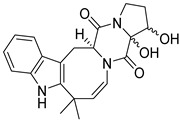
28	Ph AfPh	16β,17α-dihydroxy-deoxydihydroisoaustamide		6.0	382.1748 [M+H]^+^	382.1761	−3.4	
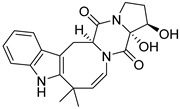
29	Ph AfPh	16α,17α-dihydroxy-deoxydihydroisoaustamide		6.6	382.1748 [M+H]^+^	382.1761	−3.4	
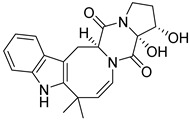
30	Ph AfPh	3β-hydroxydeoxyisoaustamide		2.9	364.1655 [M+H]^+^	364.1656	−0.3	
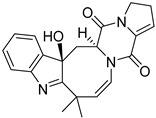
31	Ph AfPh	deoxy-14,15-dehydroisoaustamide		10.8	346.1544 [M+H]^+^	346.1550	−1.7	
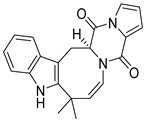
32	Ph AfPh	(+)-deoxyisoaustamide		7.7	348.1713 [M+H]^+^	348.1707	1.7	
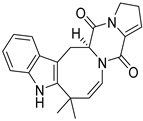
33	Ph AfPh	desoxybrevianamide E		9.6	352.2019 [M+H]^+^	352.2020	−0.3	
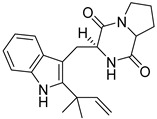
34	Ph AfPh	brevianamide F		5.0	284.1388 [M+H]^+^	284.1394	−2.1	
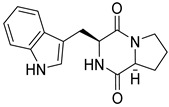
35	Ph AfPh	austamide		5.3	364.1635 [M+H]^+^	364.1656	−5.8	0.97 ^a^
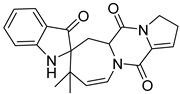
36	Ph AfPh	citreorosein		8.8	287.0539 [M+H]^+^	287.0550	−3.8	
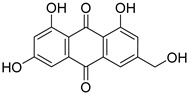
37	Ph AfPh	2-chlorocitreorosein		10.2	321.015 [M+H]^+^	321.0160	−3.1	
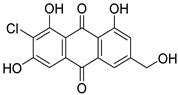
38	Ph AfPh	endocrocin		8.1	315.0488 [M+H]^+^	315.0499	−3.5	0.86 ^a^
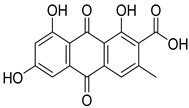
21	Ph AfPh AfAc	emodin		13.0	271.0605	271.0601	1.5	0.95 ^b^
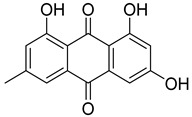
39	Ph AfPh	nephrolaevigatin A		15.5	579.0665 [M+H]^+^	579.0608	9.8	
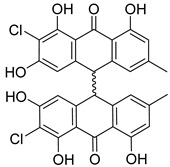
40	Ph AfPh	nephrolaevigatin B		15.7	579.0626 [M+H]^+^	579.0608	3.1	
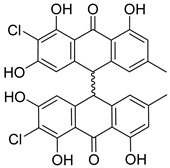
41	Ph AfPh	nephrolaevigatin C		15.3	545.1005 [M+H]^+^	545.0998	−1.3	
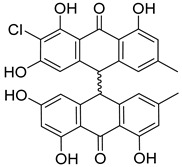
42	AfPh	nephrolaevigatin D		15.0	545.0992 [M+H]^+^	545.0998	−1.1	
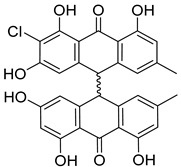
43	Ph AfPh	7-hydroxy-3-(2-hydroxypropyl)-5-methylisochromen-1-one		5.2	235.0949 [M+H]^+^	235.0965	−6.8	
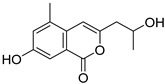
44	AfPh	3,4-dimethoxycinnamic acid		4.9	209.0805 [M+H]^+^	209.0804	0.5	
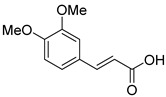
45	As AfAs	oxirapentyn B		9.7	335.1487 [M+H]^+^	335.1489	−0.6	
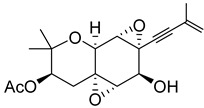
46	As AfAs	oxirapentyn E		7.2	275.1279 [M-H_2_O+H]^+^	275.1278	0.4	
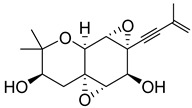
47	As AfAs	oxirapentyn F		7.1	353.1587 [M+H]^+^	353.1595	−2.3	
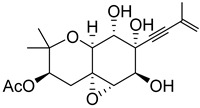
48	AfAs	oxirapentyn G		2.2	311.1504 [M+H]^+^	311.1489	4.8	
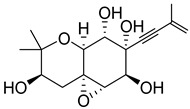
49	As AfAs	oxirapentyn H		2.9	371.1704 [M+H]^+^	371.1700	1.1	
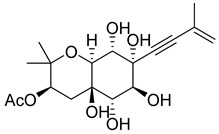
50	As AfAs	oxirapentyn I		2.9	371.1704 [M+H]^+^	371.1700	1.1	
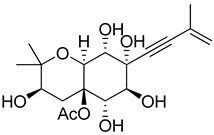
51	As AfAs	oxirapentyn J		6.2	353.1587 [M+H]^+^	353.1595	−2.3	
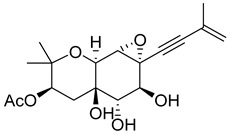
52	As AfAs	isariketide A		5.7	313.0910 [M+H]^+^	313.0918	−2.6	
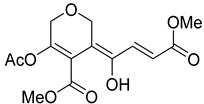
53	As AfAs	acremine S		3.7	207.1014 [M+H]^+^	207.1016	−1.0	
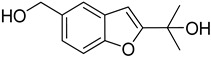
54	As AfAs	diorcin		10.4	231.1009 [M+H]^+^	231.1016	−3.0	
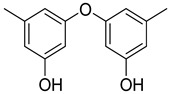
55	As AfAs	isaridin E		13.7	656.4010 [M+H]^+^	656.4018	−1.2	0.81 ^a^
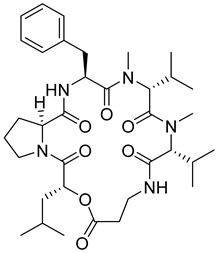
56	As AfAs	isaridin B		13.5	596.3997 [M+H]^+^	596.4018	−3.5	
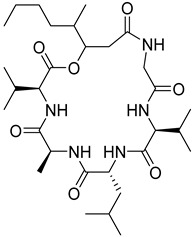
57	As AfAs	isarfelin A		14.0/14.1	670.4187 [M+H]^+^	670.4174	1.9	
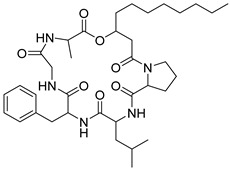
58	As AfAs	isariin		16.0	638.4495 [M+H]^+^	638.4487	1.3	
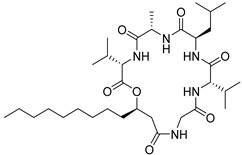
59	AfAs	isariin C		11.8	568.3692 [M+H]^+^	568.3705	−2.3	
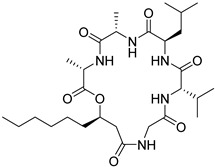
60	As AfAs	psuedodestruxin C		14.0/14.1	670.4187 [M+H]^+^	670.4174	1.9	
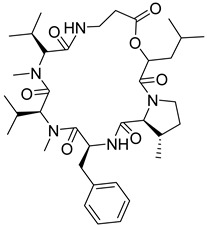
61	As AfAs	desmethylisaridin E		12.8/13.2	642.3870 [M+H]^+^	642.3861	1.4	
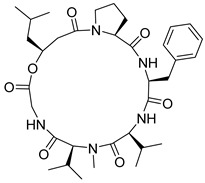
62	As AfAs	isaridin F		12.8/13.2	642.3870 [M+H]^+^	642.3861	1.4	
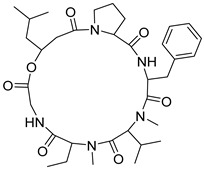
63	As AfAs	1,4a-dimethyl-9-oxo-7-propan-2-yl-3,4,10,10a-tetrahydro-2H-phenanthrene-1-carboxylic acid		14.4	315.1947 [M+H]^+^	315.1955	−2.5	0.96 ^a^
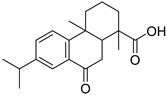
64	As	viridicatol		7.5	254.0795 [M+H]^+^	254.0812	−6.7	0.77 ^a^
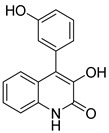
65	AfAs	isariin D		9.7	540.3395 [M+H]^+^	540.3392	−0.6	
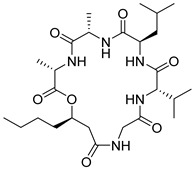
66	Ps AfPs	quinolactacide		5.4	237.0670 [M+H]^+^	237.0659	4.6	
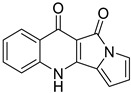
67	Ps AfPs	3,5-dimethyl-8-methoxy-3,4-dihydro-1H-isochromene-6-ol		8.7	209.1175 [M+H]^+^	209.1172	1.4	
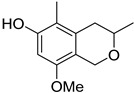
68	Ps AfPs	anserinone B		4.6	193.0856 [M−H_2_O+H]^+^	193.0859	−1.6	
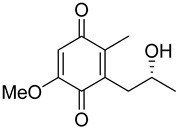
69	Ps	formylanserinone B		7.5	239.0913 [M+H]^+^	239.0914	−0.4	
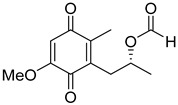
70	Ps AfPs	(6-methyl curvulinic acid		5.3	207.0651 [M−H_2_O+H]^+^	207.0652	−0.5	
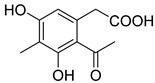
71	Ps AfPs	4-methoxyisoquinolin-1(2H)-one		2.6	176.0702 [M+H]^+^	176.0706	−2.3	
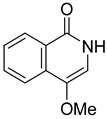
72	Ps AfPs	N,N-diethyl-3-methylbenzamide		9.7	192.1390 [M+H]^+^	192.1383	3.6	
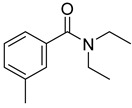
73	Ps AfPs	4-hydroxy-3,6-dimethyl-2-pyrone		2.7	141.0548 [M+H]^+^	141.0546	1.4	
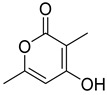
74	Ps AfPs	hydroxy-N-acetyl-β-oxotriptamine		2.4	233.0925 [M+H]^+^	233.0921	1.7	
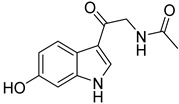
75	Ps AfPs	citriperazine D		4.4	339.0979 [M−H_2_O+H]^+^	339.0976	0.9	
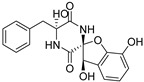
76	Ps AfPs	pretrichodermamide C		5.4	513.0988 [M+H]^+^	513.1996	−1.6	
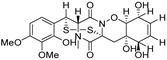
77	Ps AfPs	N-methylpretrichodermamide B		8.1	531.0662 [M+H]^+^	531.0657	0.9	
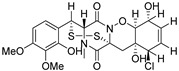
78	Ps AfPs	pretrichodermamide D		5.9	513.0988 [M+H]^+^	513.0996	−1.6	
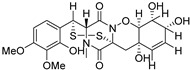
79	Ps AfPs	4-hydroxyscytalone		2.2	211.0603 [M+H]^+^	211.0601	1.0	
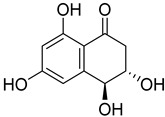
80	Ps AfPs	4-hydroxy-6-dehydroxyscytalone		3.0	195.0654 [M+H]^+^	195.0652	1.0	
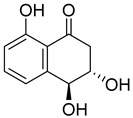
81	AfPs	3-methylorsellinic acid		2.6	183.0659	183.0652	3.8	
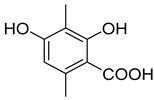
82	Ac	acruciquinone A		6.4	277.1063 [M+H]^+^	277.1071	−2.9	
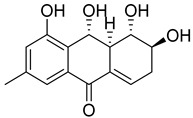
83	Ac	acruciquinone C		4.6	279.1226 [M+H]^+^	279.1227	−0.4	
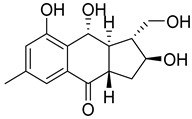
84	Ac	pleosporon		3.8	291.0853 [M+H]^+^	291.0863	−3.4	
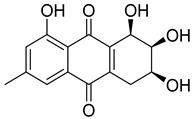
85	Ac	coniothyrinone D		4.9	279.1226 [M+H]^+^	279.1227	−0.4	
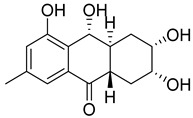
86	Ac	coniothyrinone B		7.0	263.1272 [M+H]^+^	263.1278	−2.3	
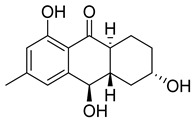
87	Ac	rubrumol		5.2	277.1062 [M+H]^+^	277.1071	−3.2	
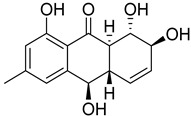
88	Ac	9,10-anthracenedione		10.1	255.0652 [M+H]^+^	255.0652	0	
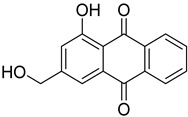
89	Ac AfAc	trans-3,4-dihydroxy-3,4-dihydroanofinic acid		4.9	239.0895 [M+H]^+^	239.0914	−8.0	
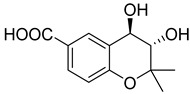
90	Ac	quadricinctapyran A		7.8	221.0819 [M+H]^+^	221.0808	5.0	
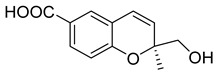
91	Ac AfAc	7-hydroxymethyl-1,2-naphthalenediol		6.2	191.0707 [M+H]^+^	191.0703	2.1	
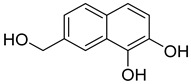
92	Ac	gliovictin		8.3	355.1160 [M+H]^+^	355.1145	4.2	
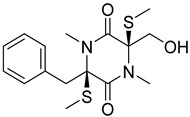
93	Ac	acrucipentyn A		4.1	231.0777 [M+H]^+^	231.0782	−2.2	
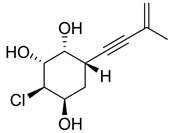
94	Ac AfAc	indolelactic acid		4.6	206.0810 [M+H]^+^	206.0812	−1.0	0.98 ^a^
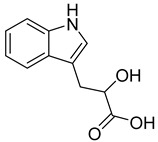
22	AfAc	cyclotryprostatin B		7.3	426.2035 [M+H]^+^	426.2024	2.6	
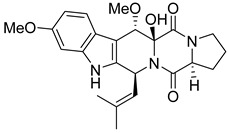
23	AfAc	bisdethiobis(methylthio)gliotoxin		7.4	357.0936 [M+H]^+^	357.0937	−0.3	
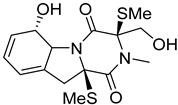

^a^ GNPS MQScore, ^b^ MetFrag Score.
