# Supplementary material for: The Metabolite Profiling of Aspergillus fumigatus KMM4631 and Its Co-Cultures with Other Marine Fungi
Source: Metabolites. 2023 Nov 8;13(11):1138. doi: 10.3390/metabo13111138 (PMC10673247; doi:10.3390/metabo13111138)
Supplement: Supplementary file 1 [file metabolites-13-01138-s001.zip › metabolites-2667434-supplementary.pdf]

SUPPLEMENTARY MATERIALS

Metabolite Profiling of *Aspergillus fumigatus* KMM4631 and its Co-Cultures with Other Marine Fungi

Contents

Figure S1. UHPLC MS chromatogram of extract of *Aspergillus fumigatus* KMM 4631 monoculture. 4

Figure S2. UHPLC MS chromatogram of extract of *Penicillium hispanicum* KMM 4689 monoculture. 4

Figure S3. UHPLC MS chromatogram of extract of *Aspergillus fumigatus* KMM 4631+ *Penicillium hispanicum* KMM 4689 co-culture. 5

Figure S4. UHPLC MS chromatogram of extract of *Amphichorda* sp. KMM 4639 monoculture. 5

Figure S5. UHPLC MS chromatogram of extract of *Aspergillus fumigatus* KMM 4631+*Amphichorda* sp. KMM 4639 co-culture. 6

Figure S6. UHPLC MS chromatogram of extract of *Penicillium sp.* KMM 4672 monoculture. 6

Figure S7. UHPLC MS chromatogram of extract of *Aspergillus fumigatus* KMM 4631+*Penicillium sp.* KMM 4672 co-culture. 7

Figure S8. UHPLC MS chromatogram of extract of *Asteromyces cruciatus* KMM 4696 monoculture. 7

Figure S9. UHPLC MS chromatogram of extract of *Aspergillus fumigatus* KMM 4631+ *Asteromyces cruciatus* KMM 4696 co-culture. 8

Figure S10. MS/MS fragmentation of fumiquinazoline C (**3**) and D (**4**). 8

Figure S11. MS/MS fragmentation of fumiquinazoline K (**6**). 9

Figure S12. MS/MS of tryptoquivaline J (**9**) and F (**5**). 9

Figure S13. MS/MS fragmentation of fumiquinazoline F (**10**). 10

Figure S14. MS/MS fragmentation of pseurotin A (**11**) and D (**15**). 10

Figure S15. MS/MS fragmentation of conjugated linoleic acid (10E,12Z) (**12**). 11

Figure S16. MS/MS fragmentation of 2-chloroemodin (**13**). 11

Figure S17. MS/MS fragmentation of scytalone (**14**). 12

Figure S18. MS/MS fragmentation of agroclavine (**16**). 12

Figure S19. MS/MS fragmentation of fumigaclavine A (**17**). 13

Figure S20. MS/MS fragmentation of pyripyropene A (**18**). 13

Figure S21. MS/MS fragmentation of fumiquinazoline G (**19**). 14

Figure S22. MS/MS fragmentation of ergosterol peroxide (**20**). 14

Figure S23. MS/MS fragmentation of emodin (**21**). 15

Figure S24. MS/MS fragmentation of diorcin (**54**). 15

Figure S25. MS/MS fragmentation of isaridin E (**55**). 16

Figure S26. MS/MS fragmentation of isaridin B (**56**). 16

Figure S27. MS/MS fragmentation of isariin (**58**). 17

Figure S28. MS/MS fragmentation of psuedodestruxin C (**60**). 17

Figure S29. MS/MS fragmentation of desmethylisaridin E (**61**). 18

Figure S30. MS/MS fragmentation of isaridin F (**62**) 18

Figure S31. MS/MS fragmentation of 1,4a-dimethyl-9-oxo-7-propan-2-yl-3,4,10,10a-tetrahydro-2H-phenanthrene-1-carboxylic acid (**63**). 19

Figure S32. MS/MS fragmentation of viridicatol (**64**). 19

Figure S33. MS/MS fragmentation of citriperazine D (**75**). 20

Figure S34. MS/MS fragmentation of pretrichodermamide C (**76**). 20

Figure S35. MS/MS fragmentation of N-methylpretrichodermamide B (**77**). 21

Figure S36. MS/MS fragmentation of pretrichodermamide D (**78**) 21

Figure S37. MS/MS fragmentation of coniothyrinone B (**86**). 22

Figure S38. MS/MS fragmentation of 9,10-anthracenedione (**88**) 22

Figure S39. MS/MS fragmentation of trans-3,4-dihydroxy-3,4-dihydroanofinic acid (**89**). 23

Figure S40. MS/MS fragmentation of indolelactic acid (**94**) 23

Table S1. Experimental UPLC MS data for reference compounds 24

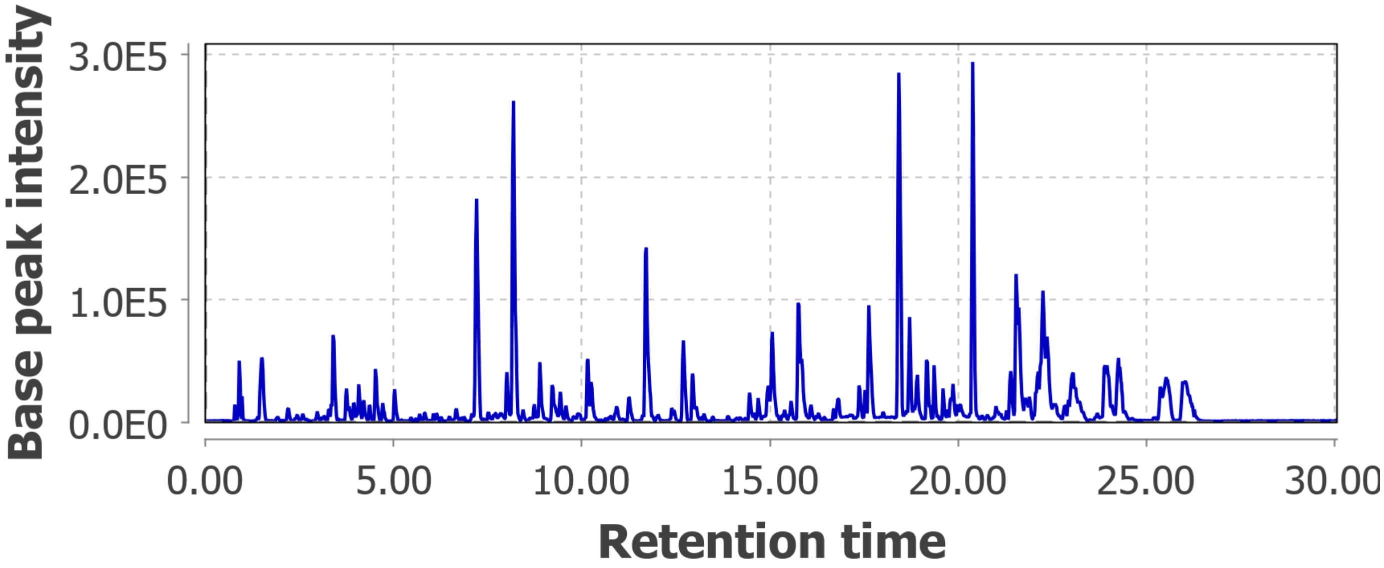

Figure S1. UHPLC MS chromatogram of extract of *Aspergillus fumigatus* KMM 4631 monoculture.

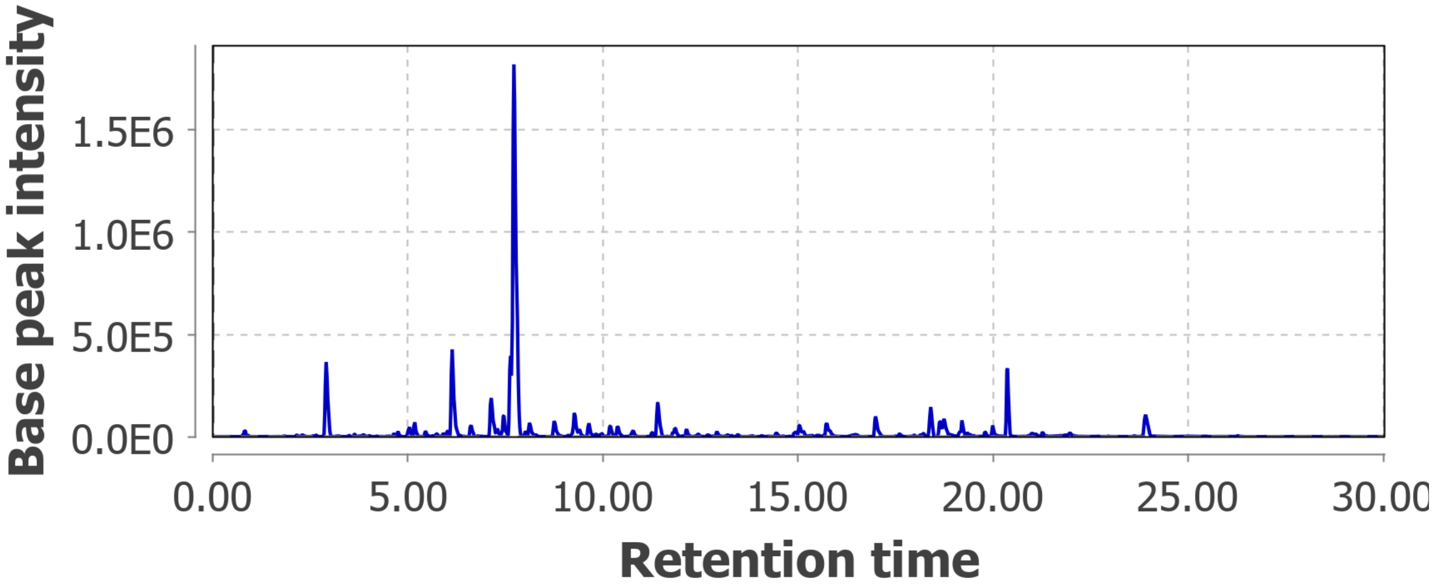

Figure S2. UHPLC MS chromatogram of extract of *Penicillium hispanicum* KMM 4689 monoculture.

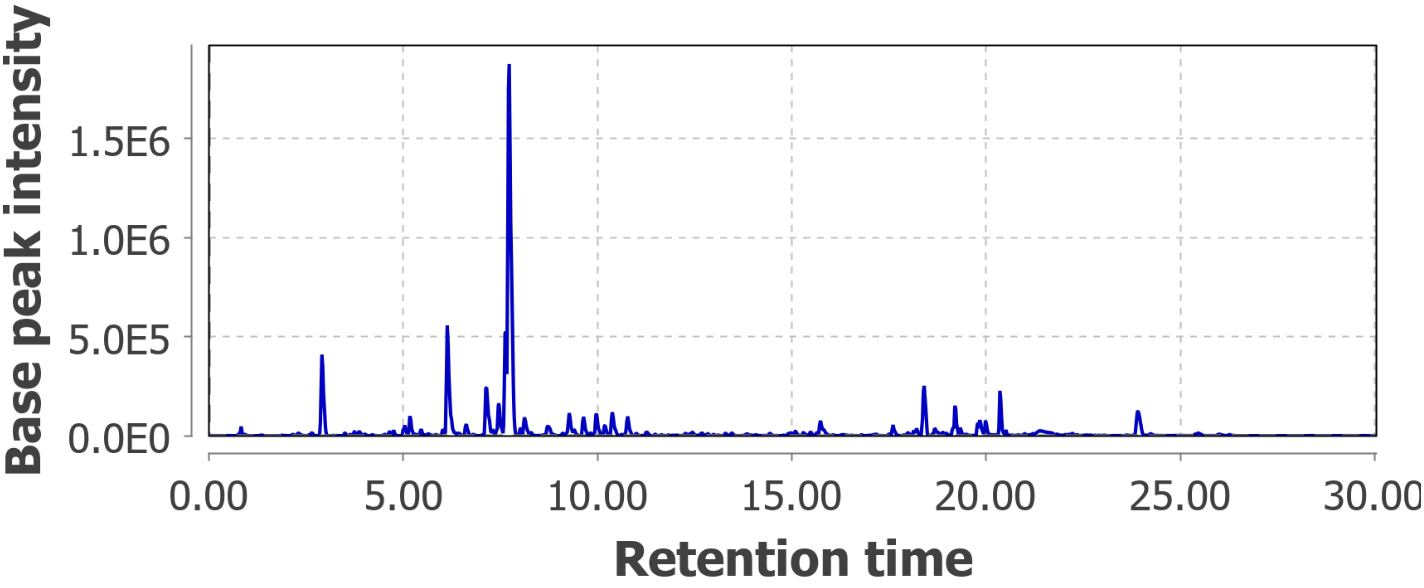

Figure S3. UHPLC MS chromatogram of extract of *Aspergillus fumigatus* KMM 4631+ *Penicillium hispanicum* KMM 4689 co-culture.

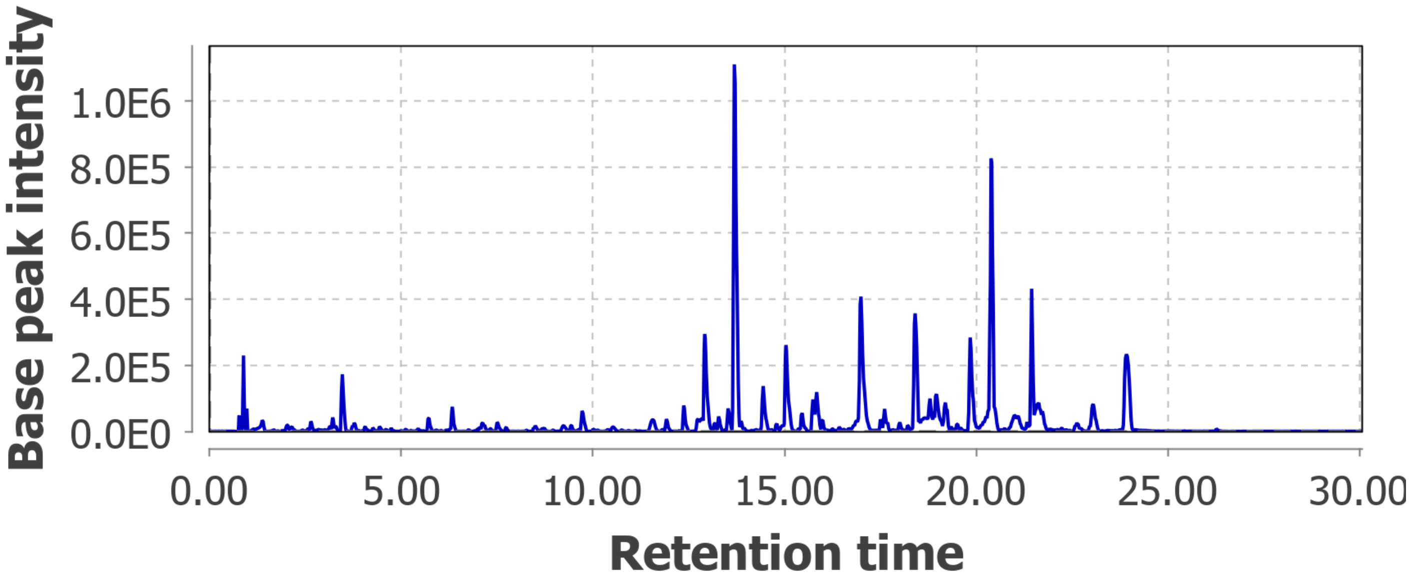

Figure S4. UHPLC MS chromatogram of extract of *Amphichorda* sp. KMM 4639 monoculture.

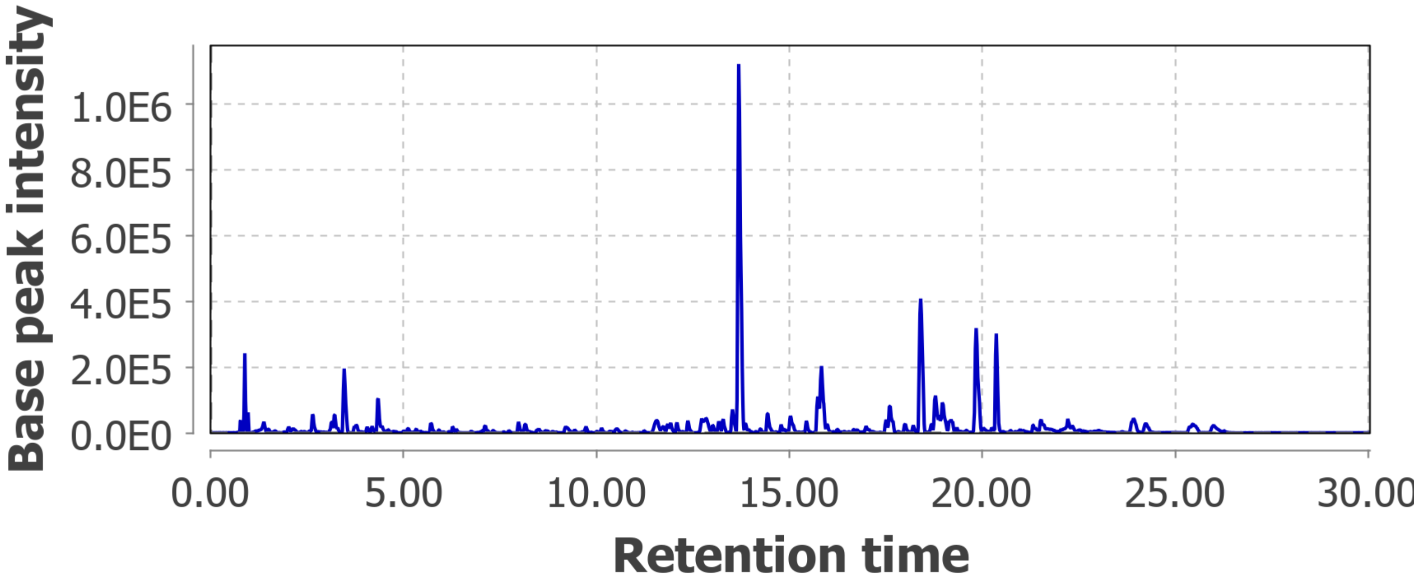

Figure S5. UHPLC MS chromatogram of extract of *Aspergillus fumigatus* KMM 4631+*Amphichorda* sp. KMM 4639 co-culture.

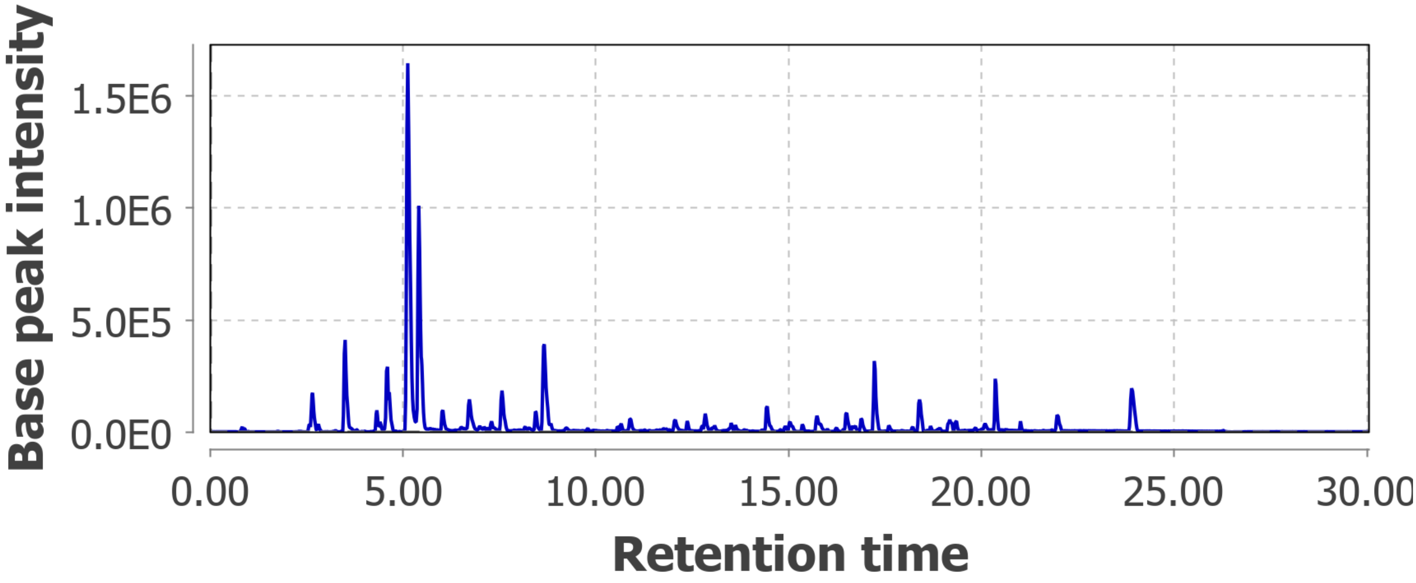

Figure S6. UHPLC MS chromatogram of extract of *Penicillium sp.* KMM 4672 monoculture.

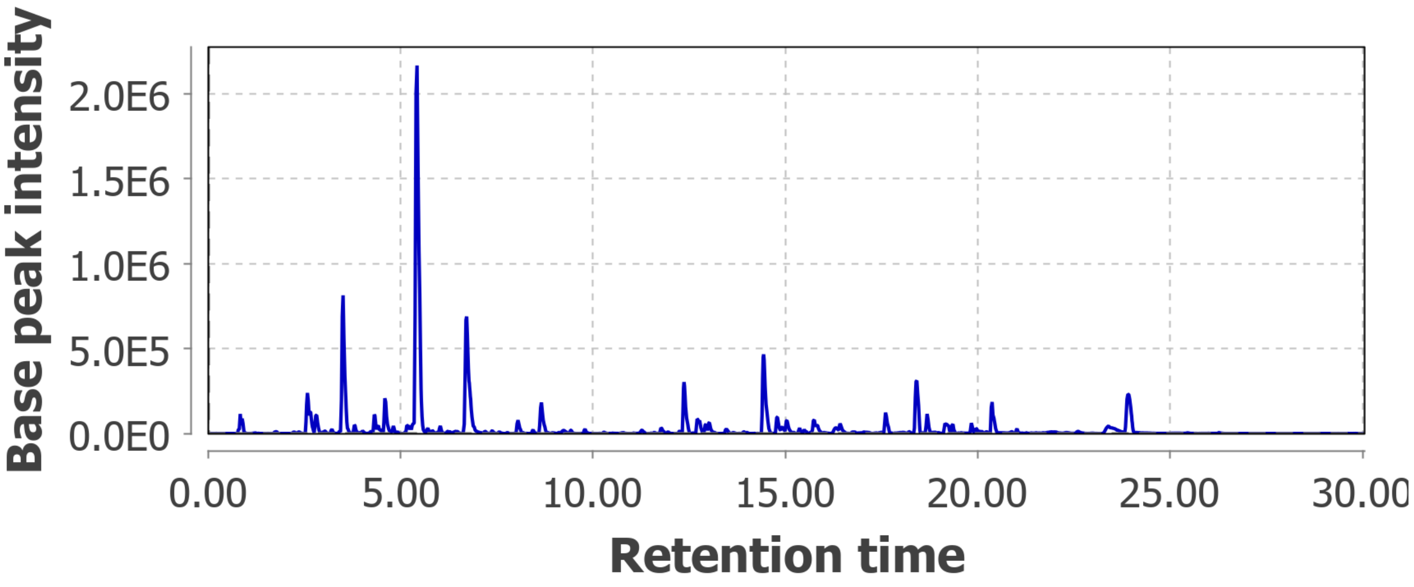

Figure S7. UHPLC MS chromatogram of extract of *Aspergillus fumigatus* KMM 4631+*Penicillium sp.* KMM 4672 co-culture.

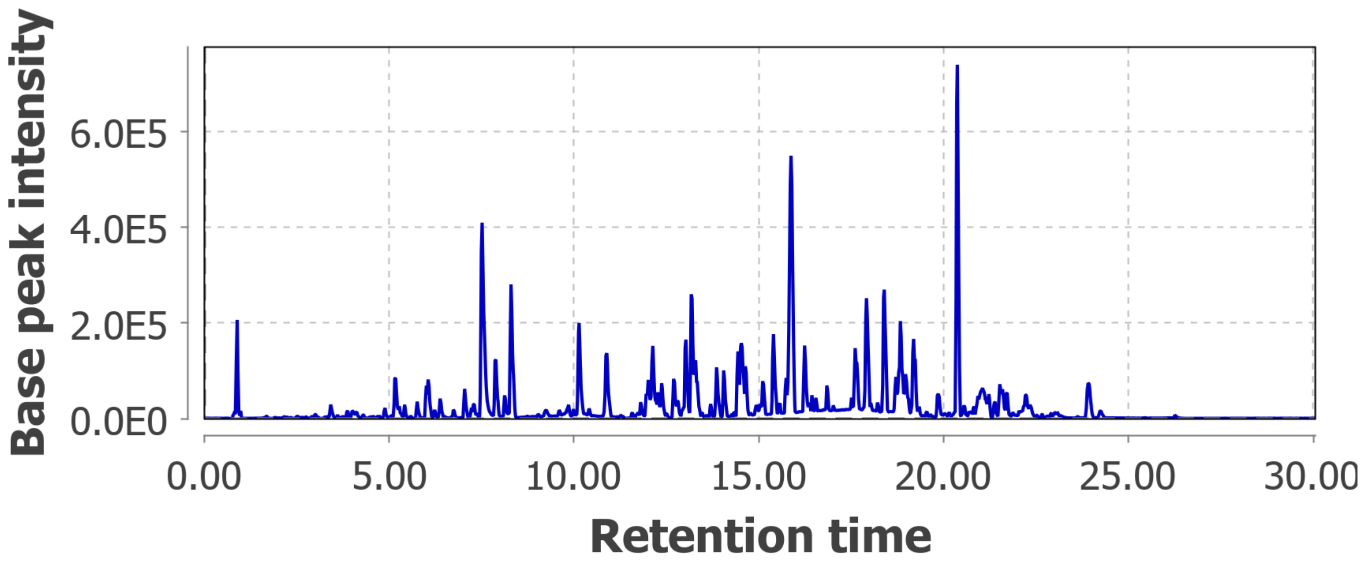

Figure S8. UHPLC MS chromatogram of extract of *Asteromyces cruciatus* KMM 4696 monoculture.

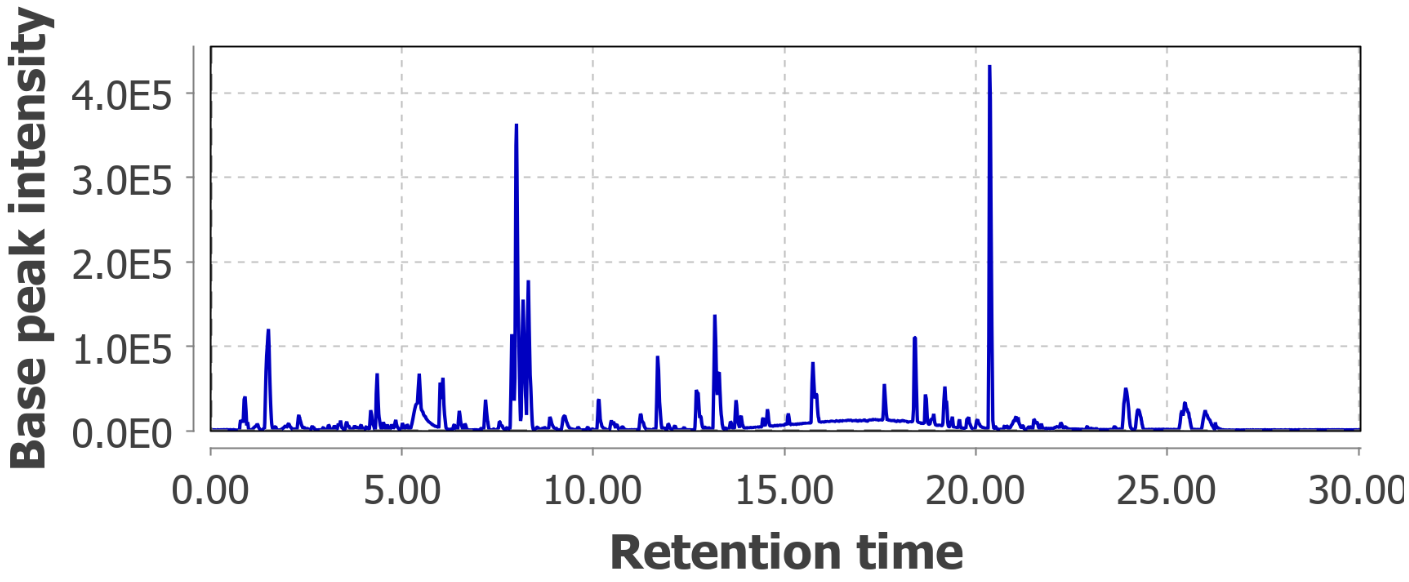

Figure S9. UHPLC MS chromatogram of extract of *Aspergillus fumigatus* KMM 4631+ *Asteromyces cruciatus* KMM 4696 co-culture.

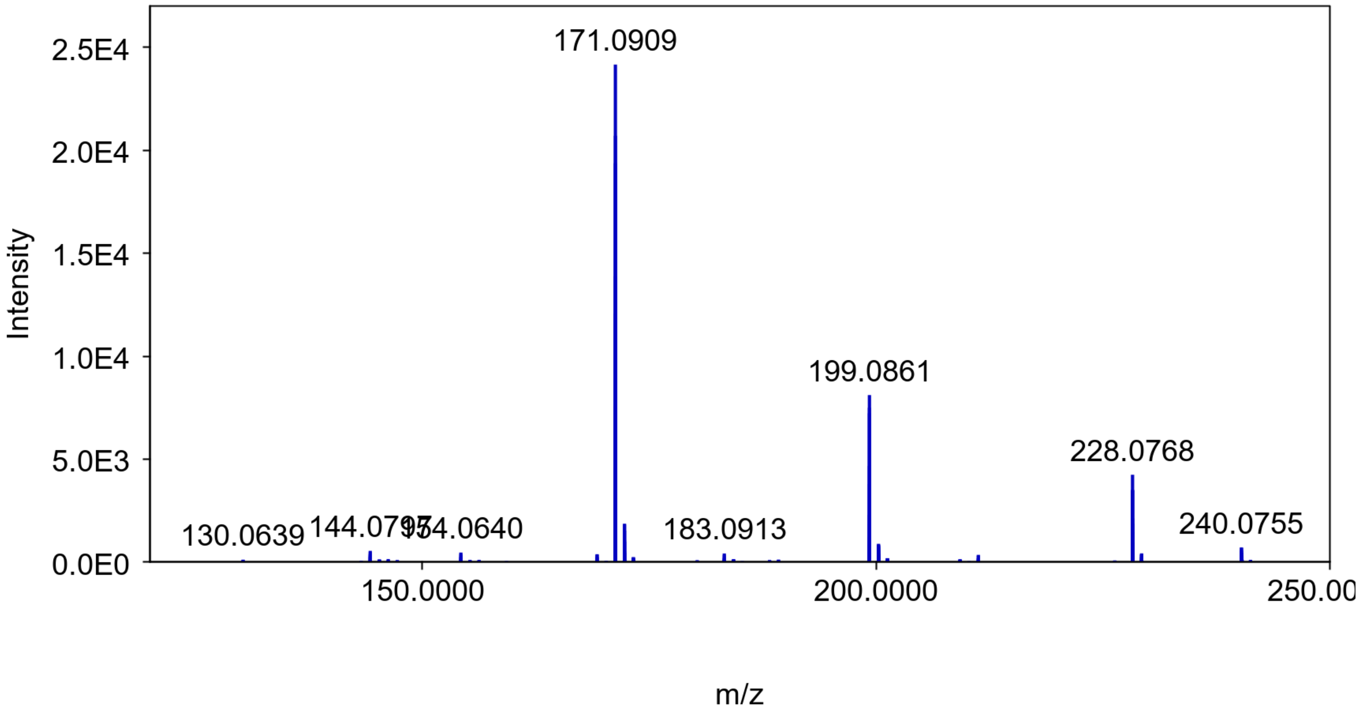

Figure S10. MS/MS fragmentation of fumiquinazoline C (**3**) and D (**4**).

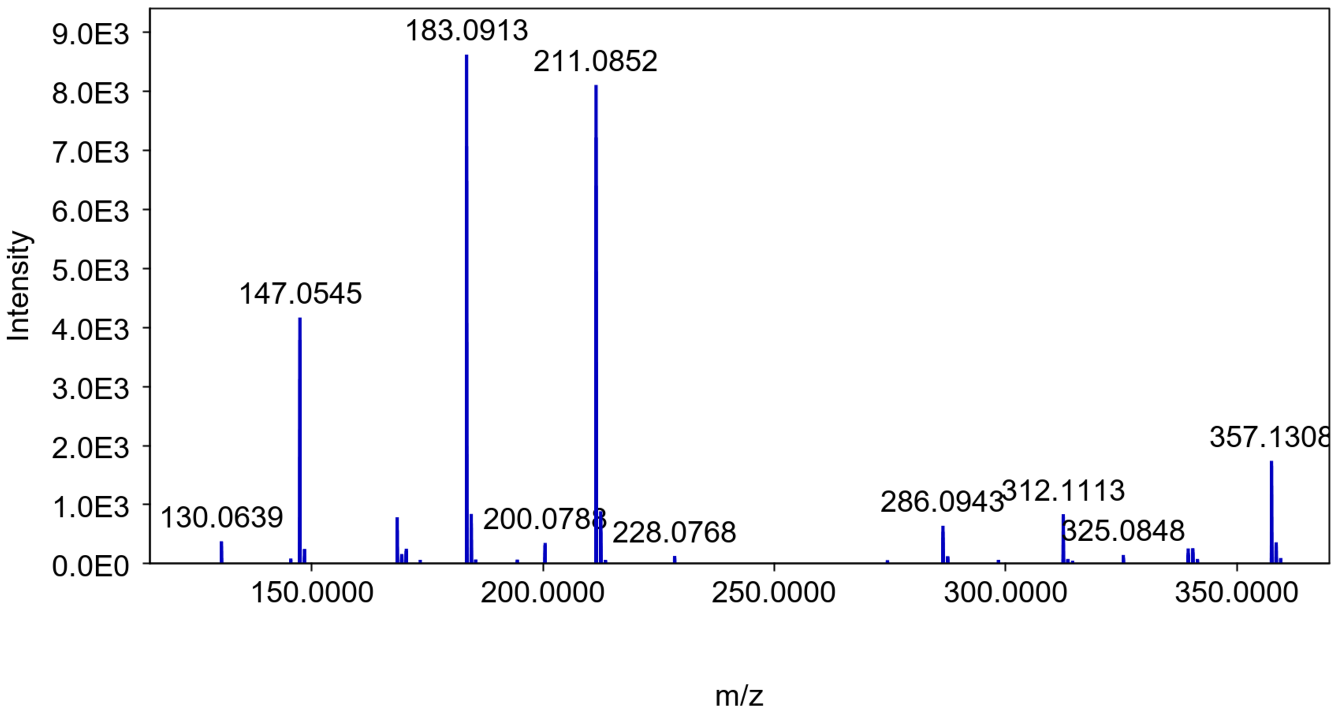

Figure S11. MS/MS fragmentation of fumiquinazoline K (**6**).

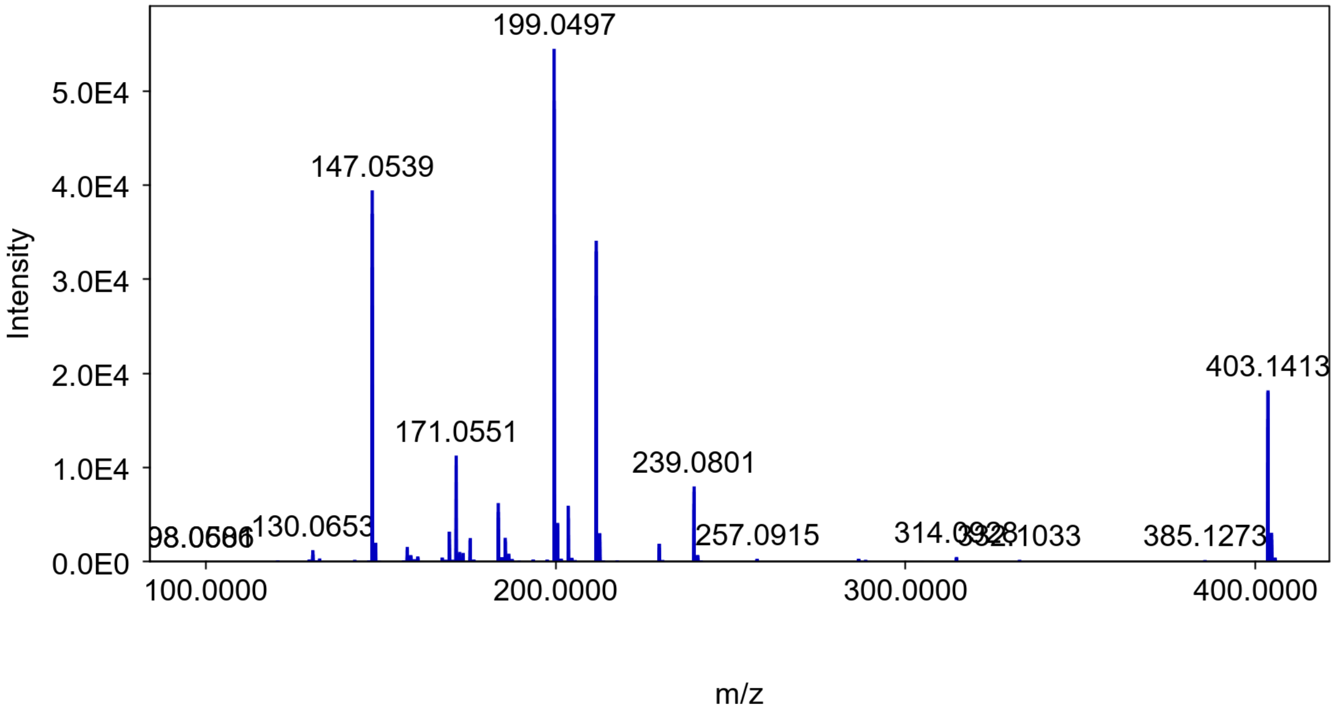

Figure S12. MS/MS of tryptoquivaline J (**9**) and F (**5**).

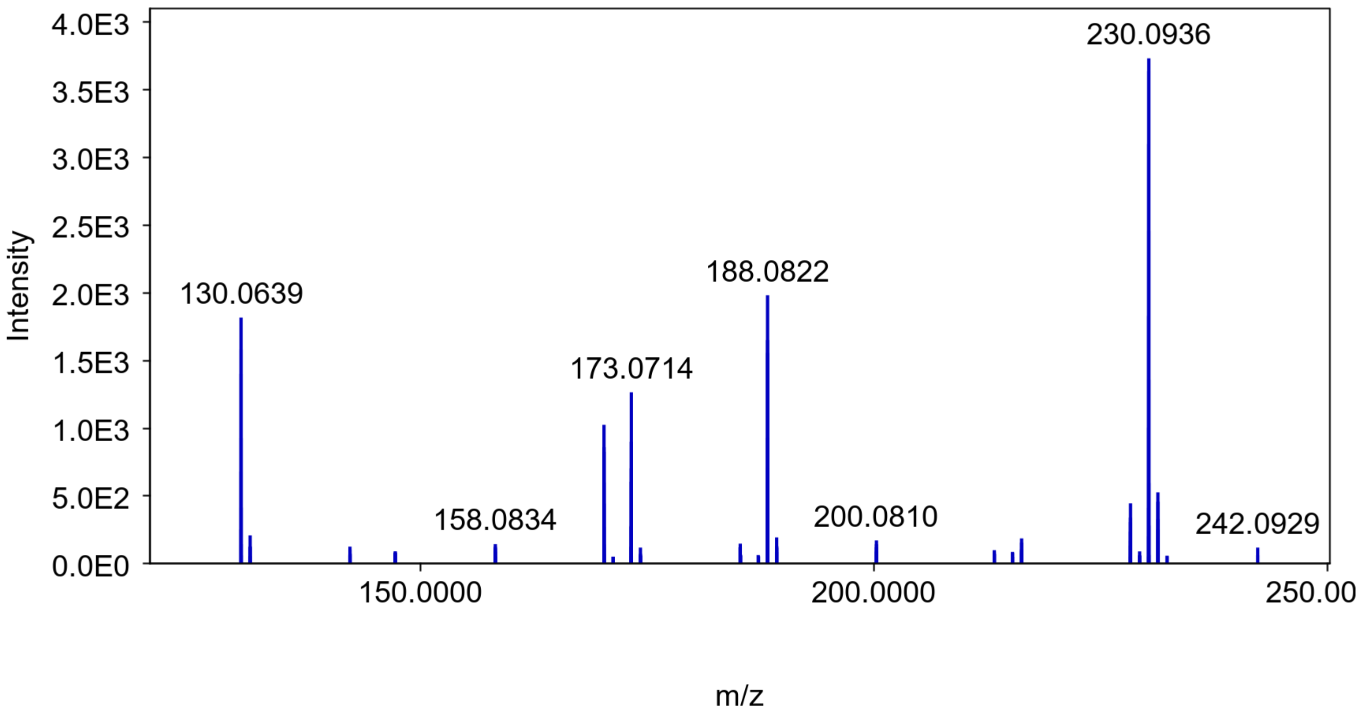

Figure S13. MS/MS fragmentation of fumiquinazoline F (**10**).

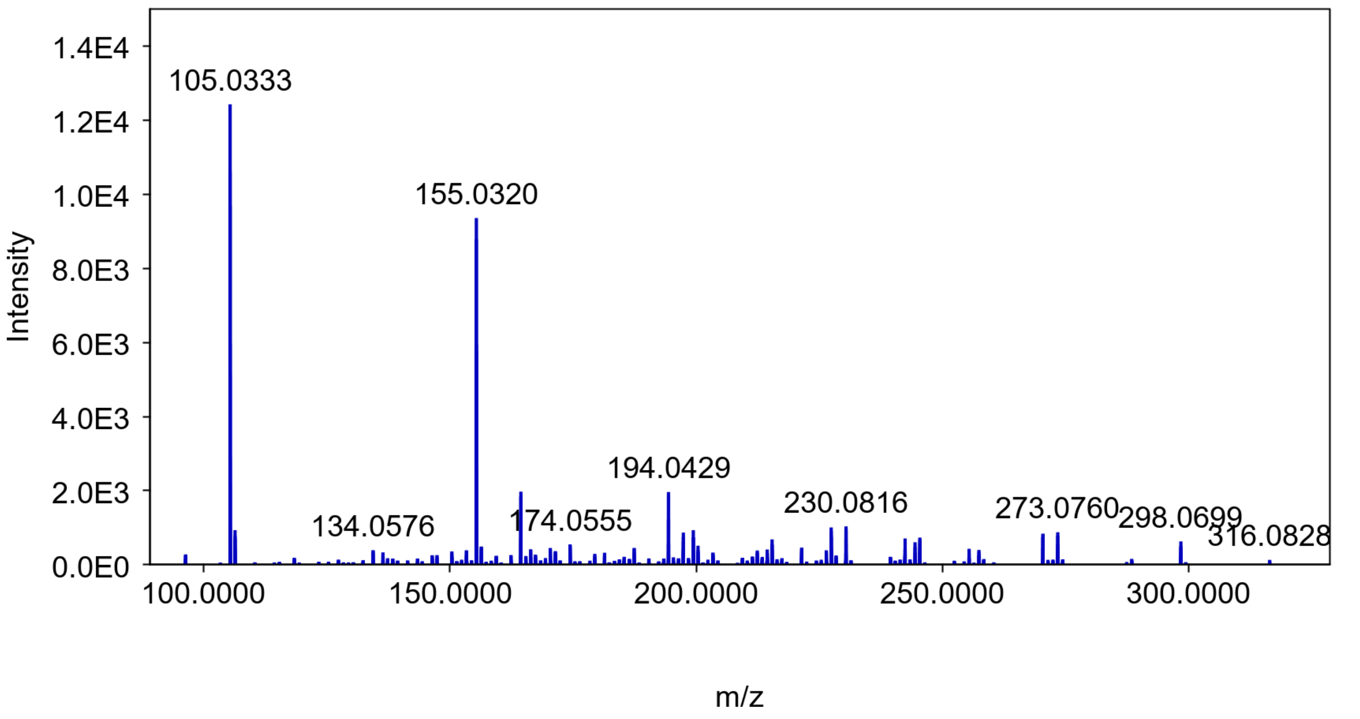

Figure S14. MS/MS fragmentation of pseurotin A (**11**) and D (**15**).

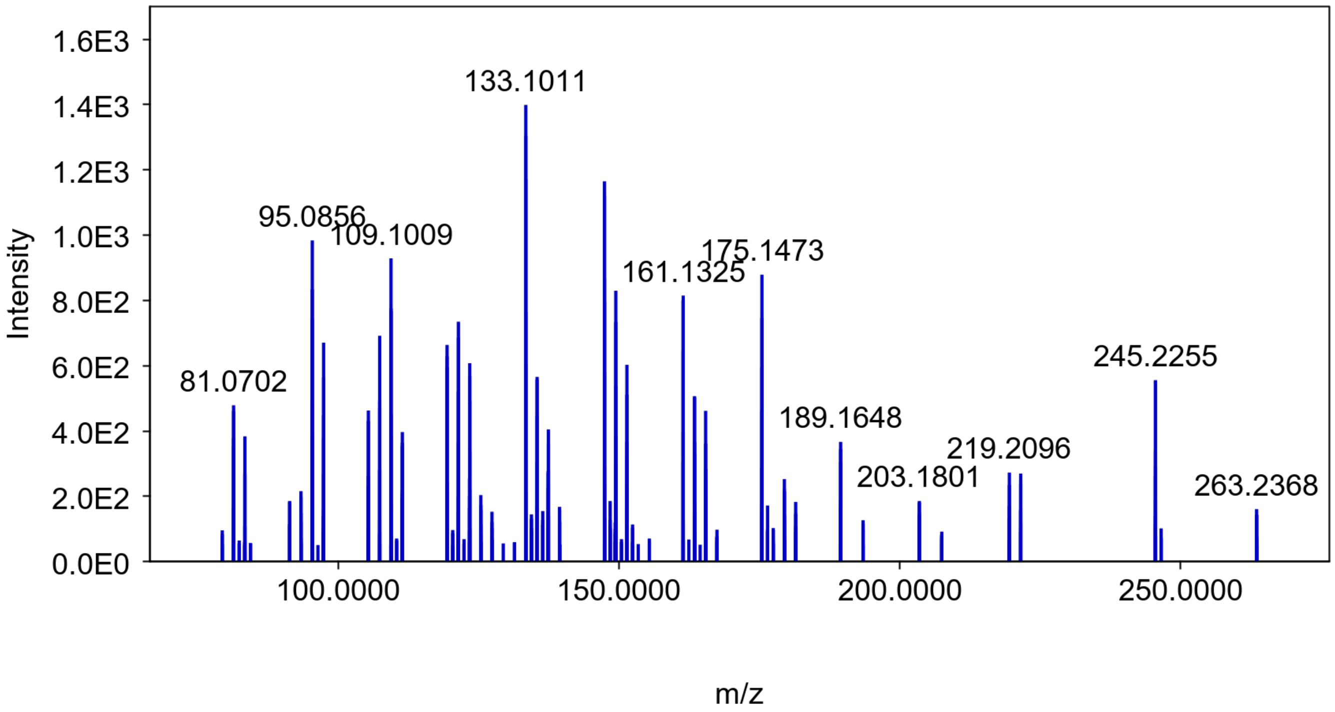

Figure S15. MS/MS fragmentation of conjugated linoleic acid (10E,12Z) (1**2**).

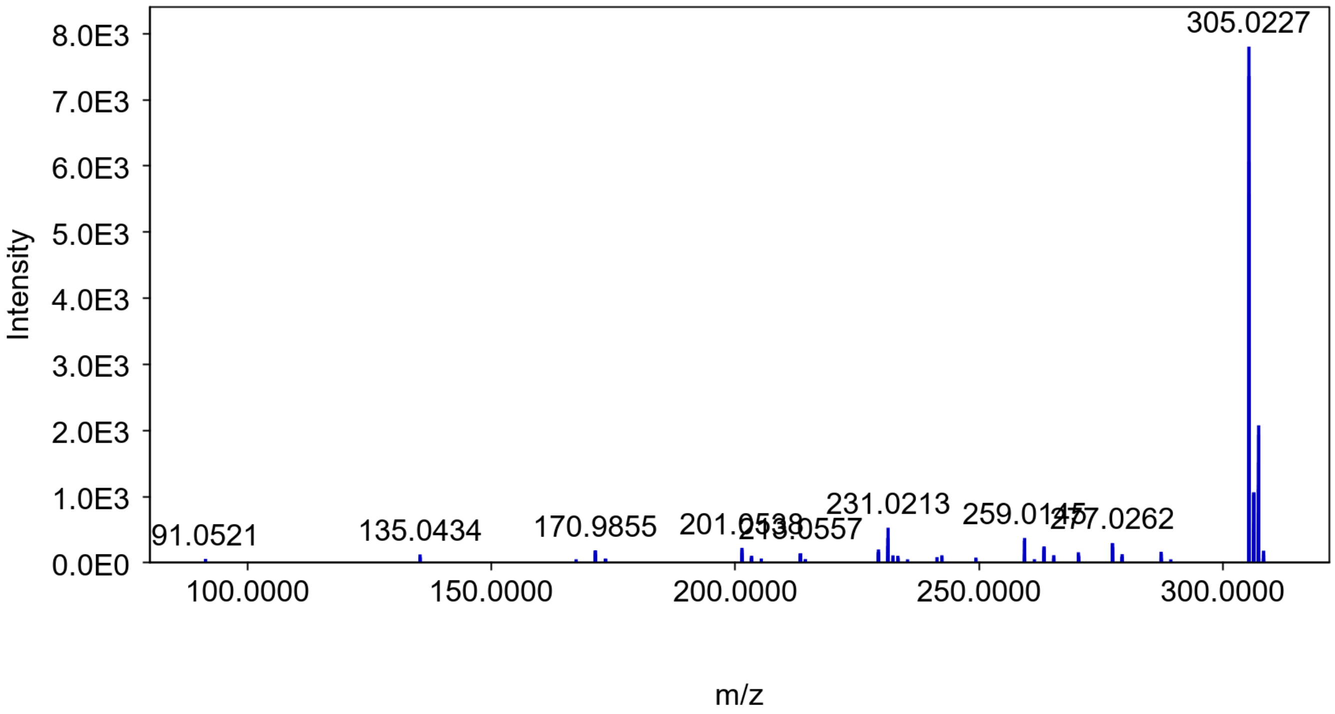

Figure S16. MS/MS fragmentation of 2-chloroemodin (1**3**).

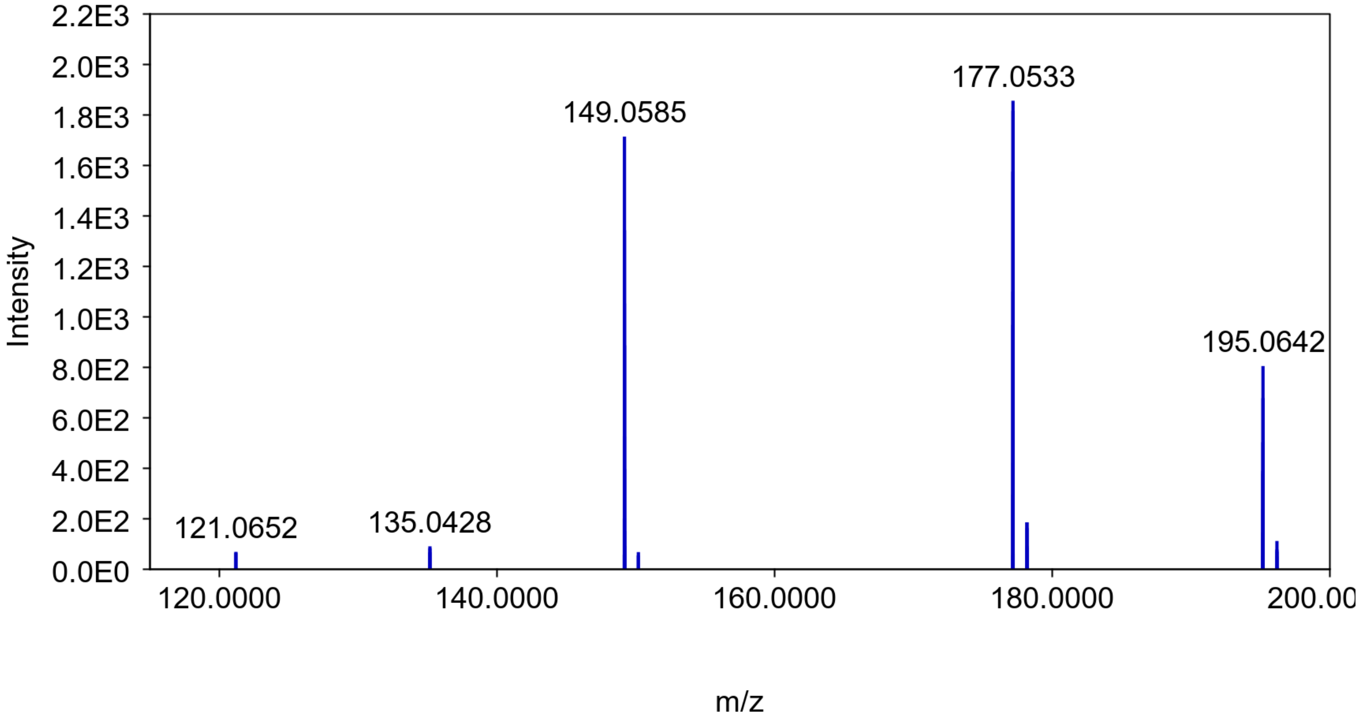

Figure S17. MS/MS fragmentation of scytalone (**14**).

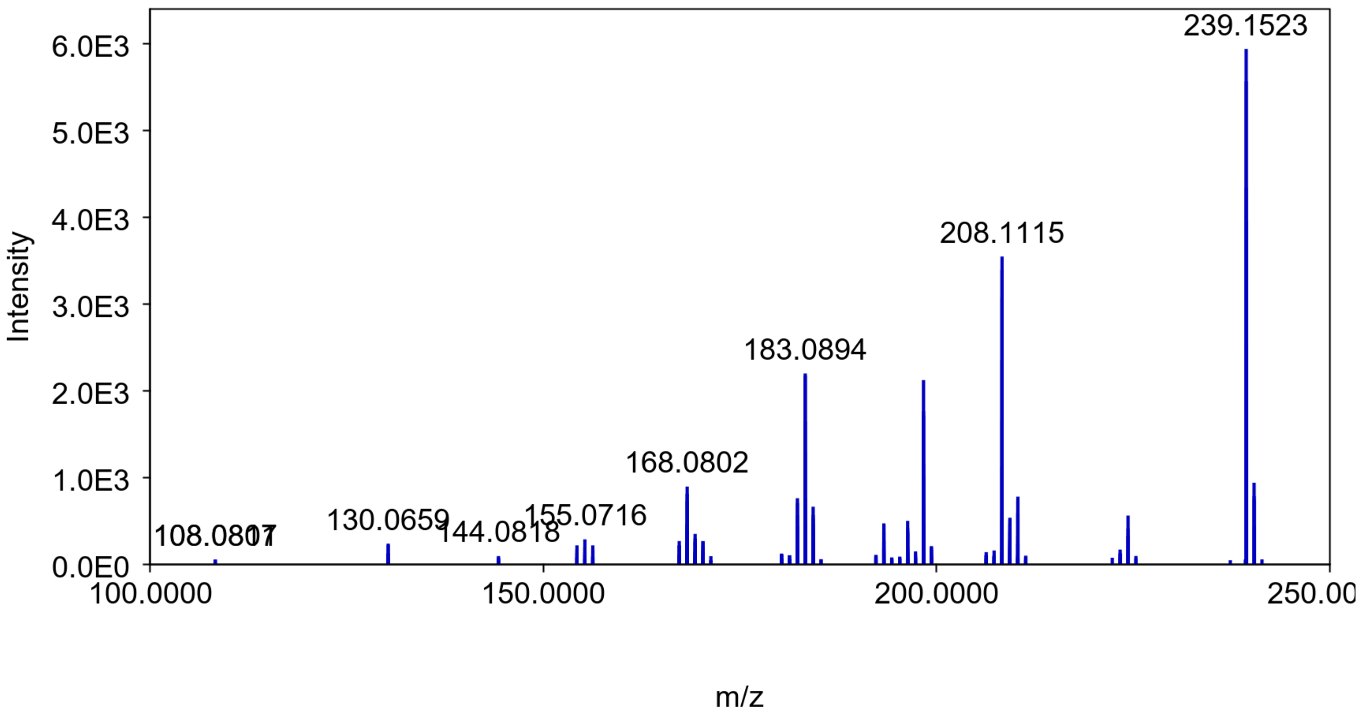

Figure S18. MS/MS fragmentation of agroclavine (**16**).

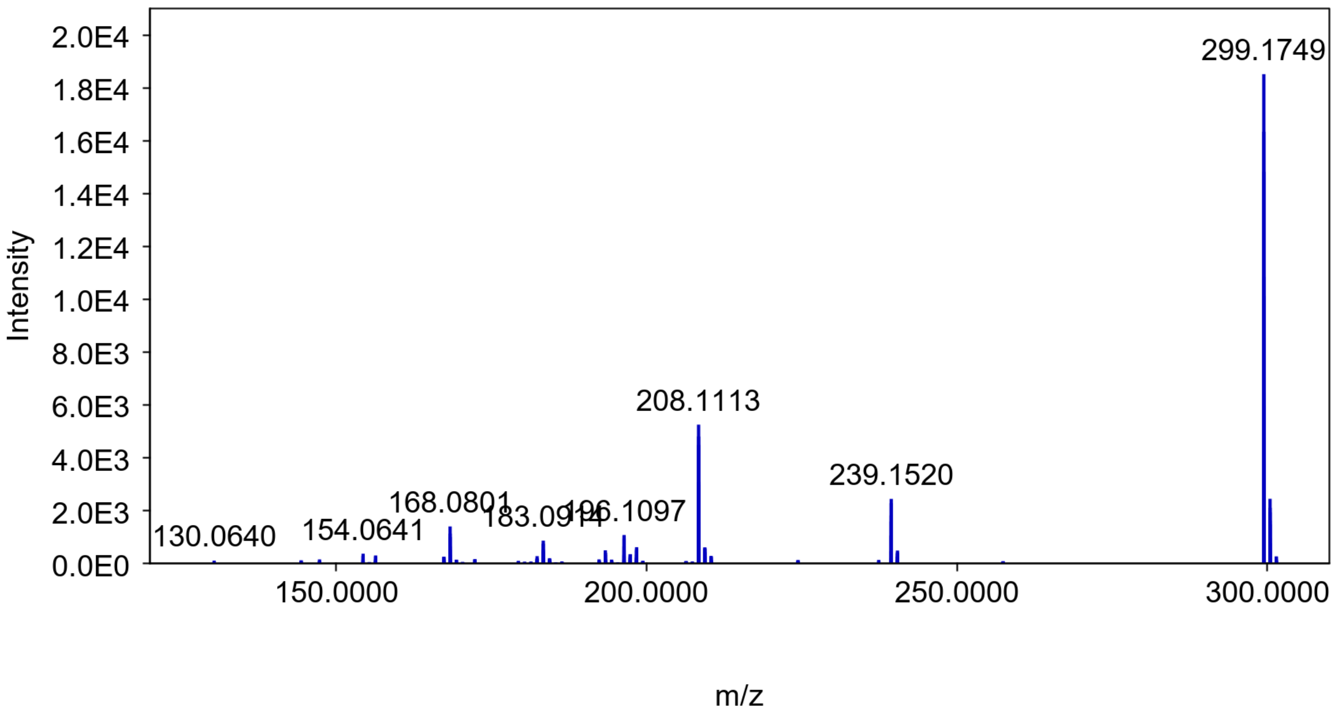

Figure S19. MS/MS fragmentation of fumigaclavine A (**17**).

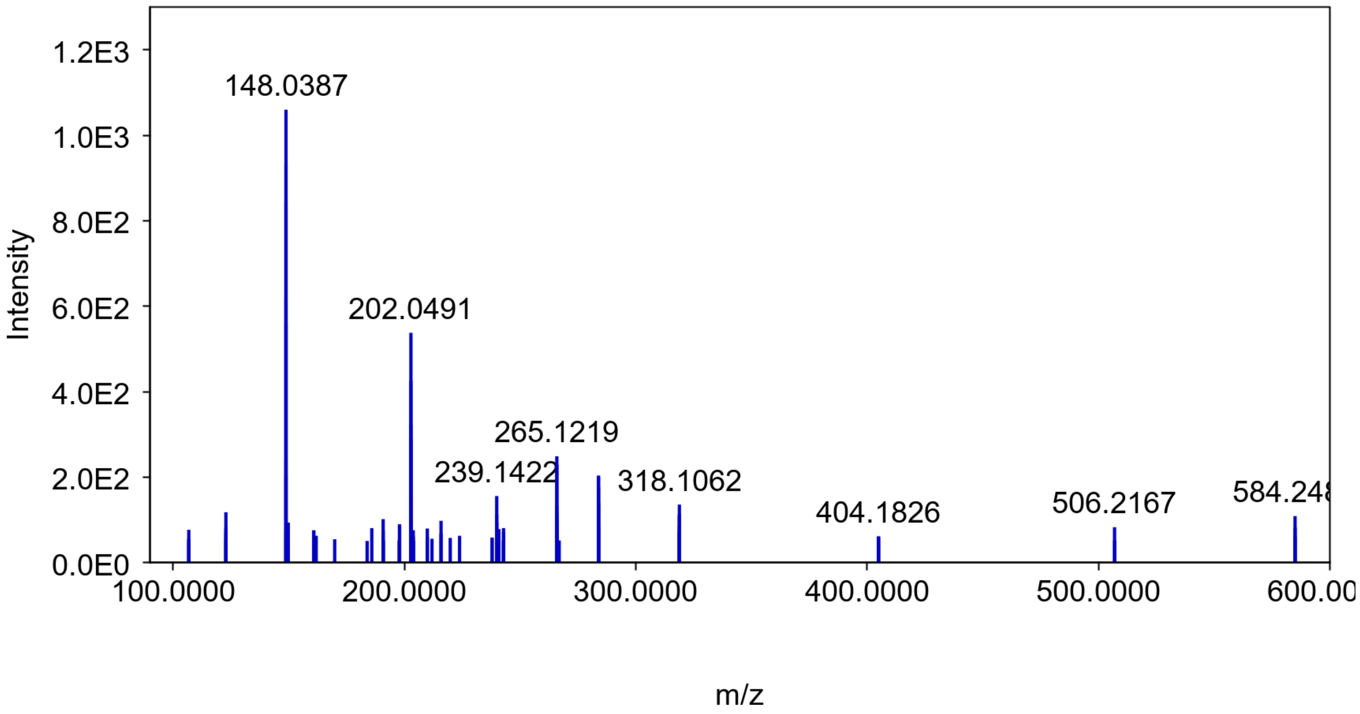

Figure S20. MS/MS fragmentation of pyripyropene A (**18**).

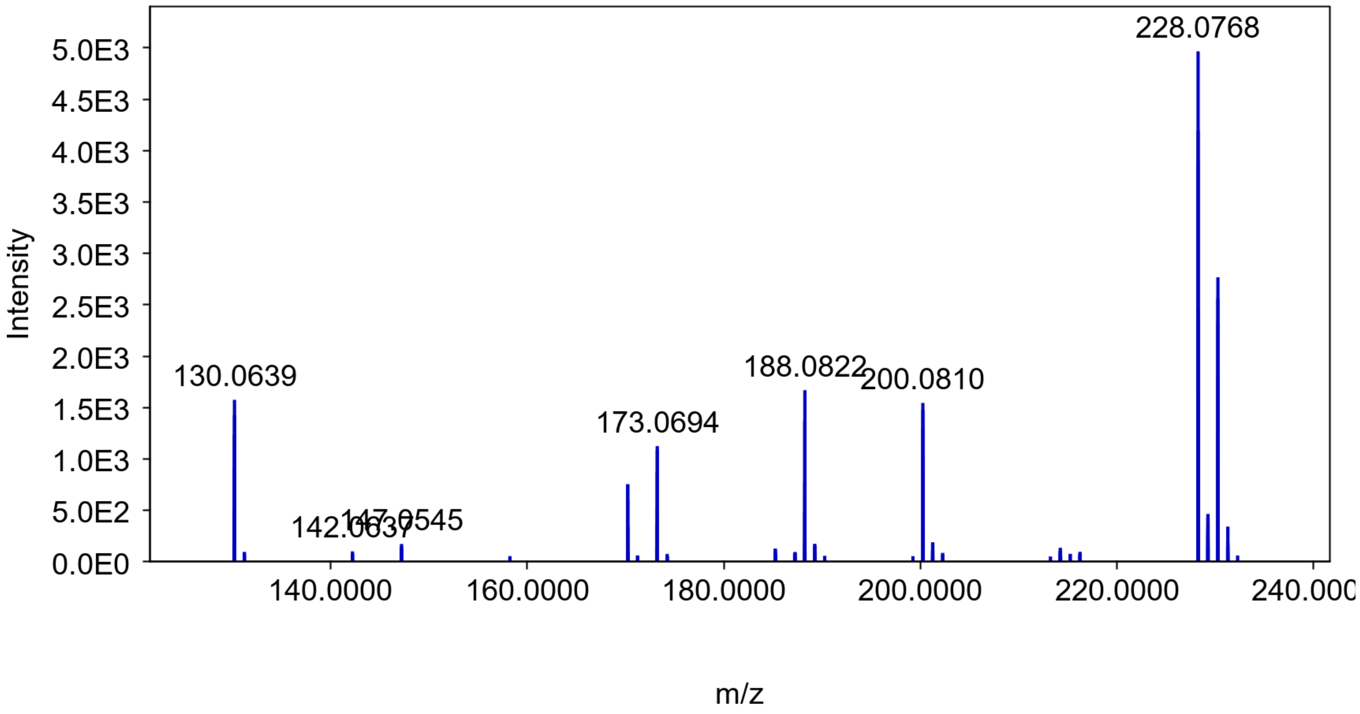

Figure S21. MS/MS fragmentation of fumiquinazoline G (**19**).

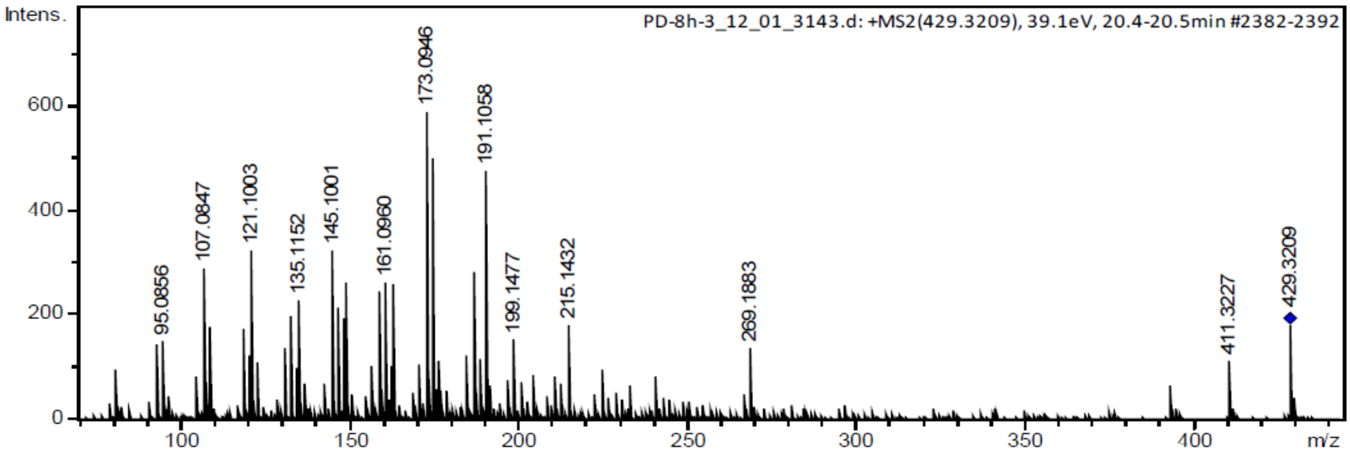

Figure S22. MS/MS fragmentation of ergosterol peroxide (**20**).

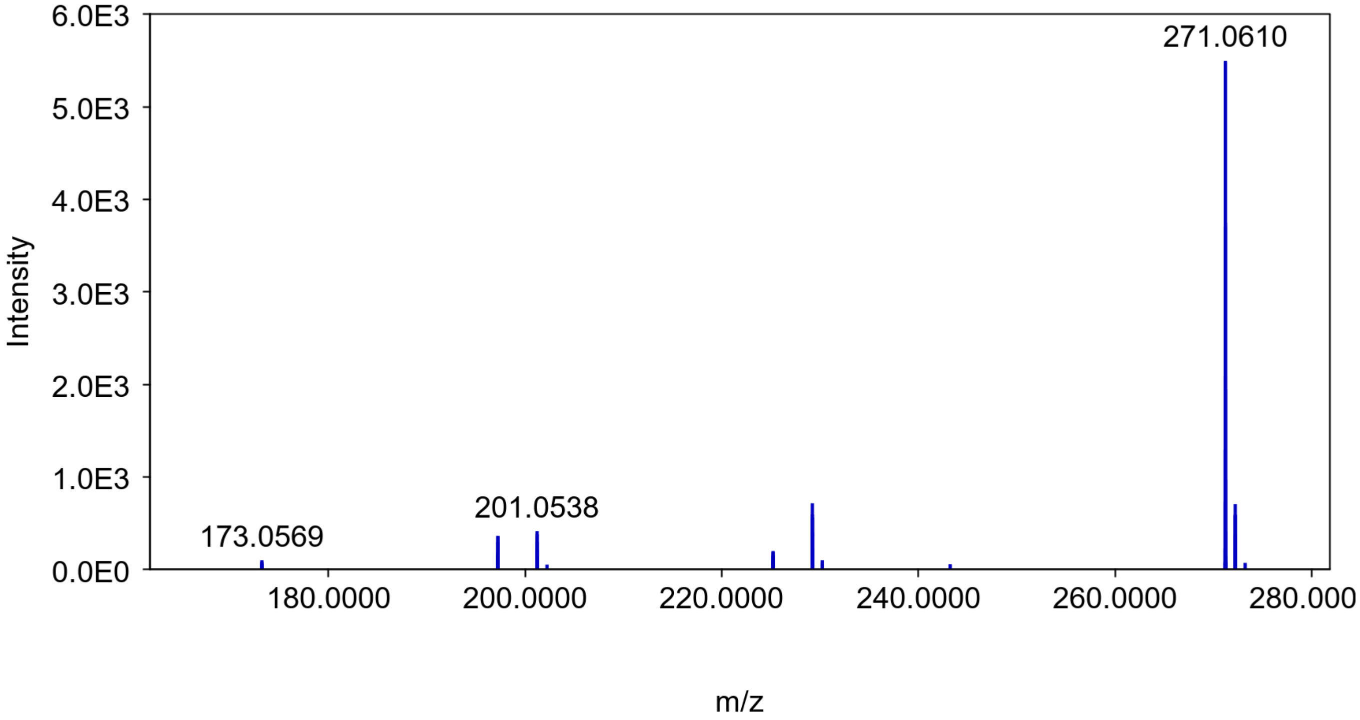

Figure S23. MS/MS fragmentation of emodin (**21**).

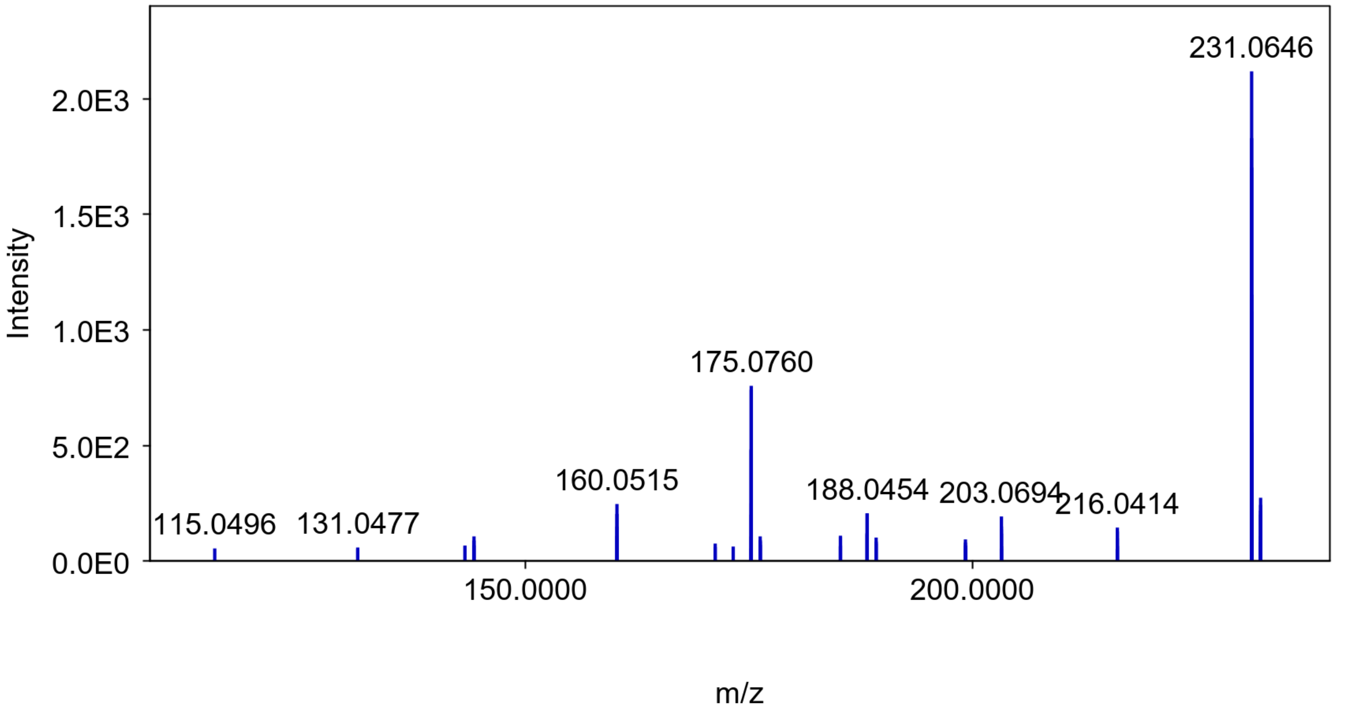

Figure S24. MS/MS fragmentation of diorcin (**54**).

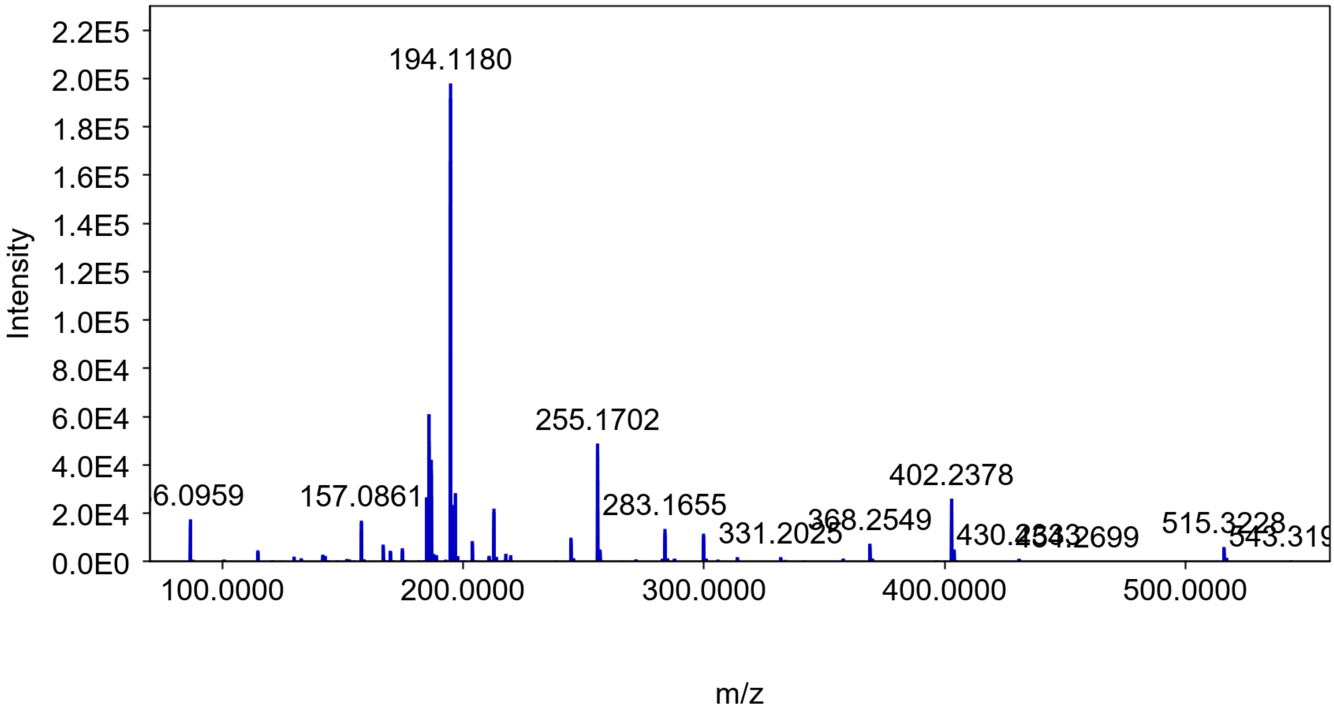

Figure S25. MS/MS fragmentation of isaridin E (**55**).

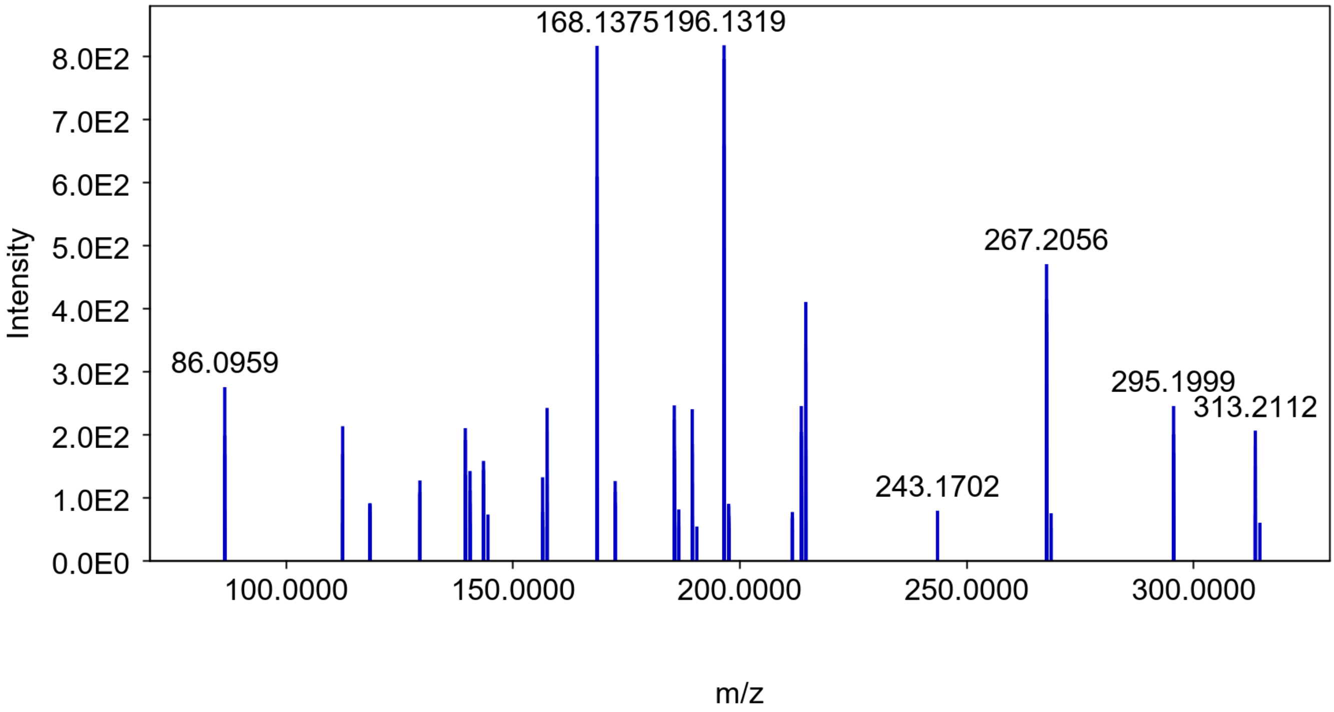

Figure S26. MS/MS fragmentation of isaridin B (**56**).

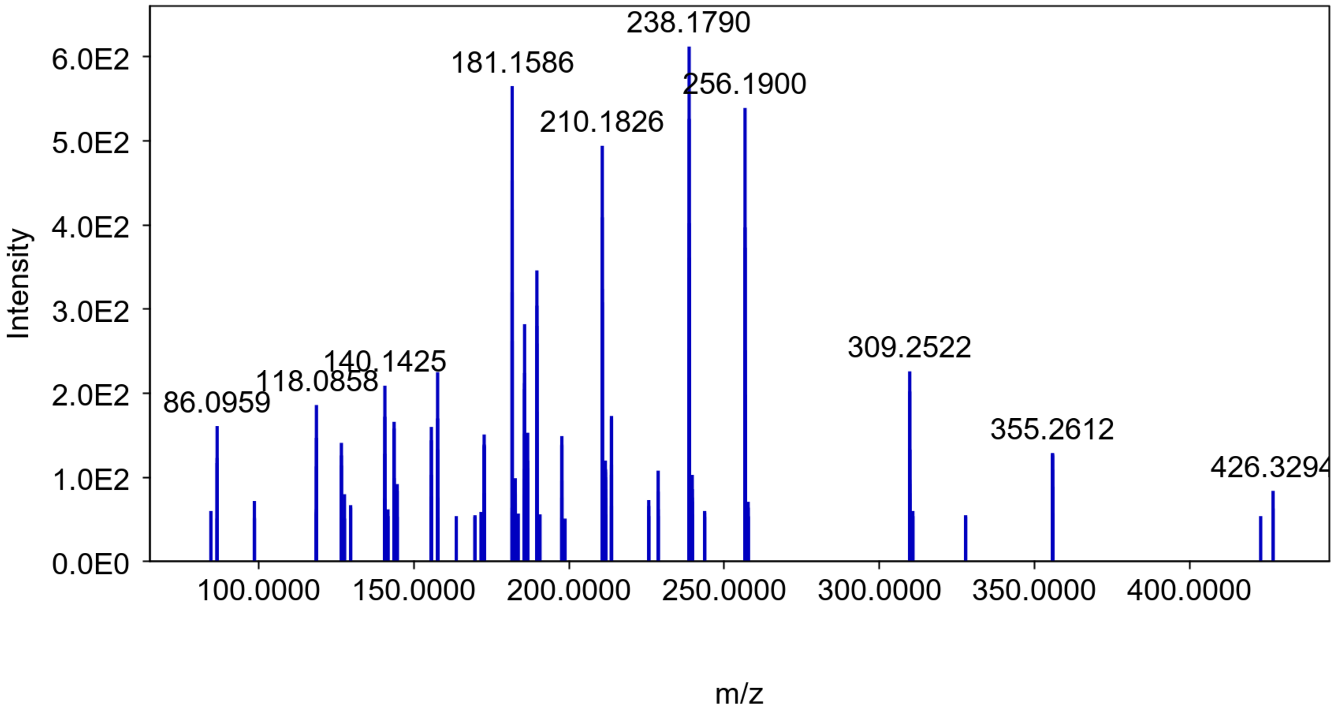

Figure S27. MS/MS fragmentation of isariin (**58**).

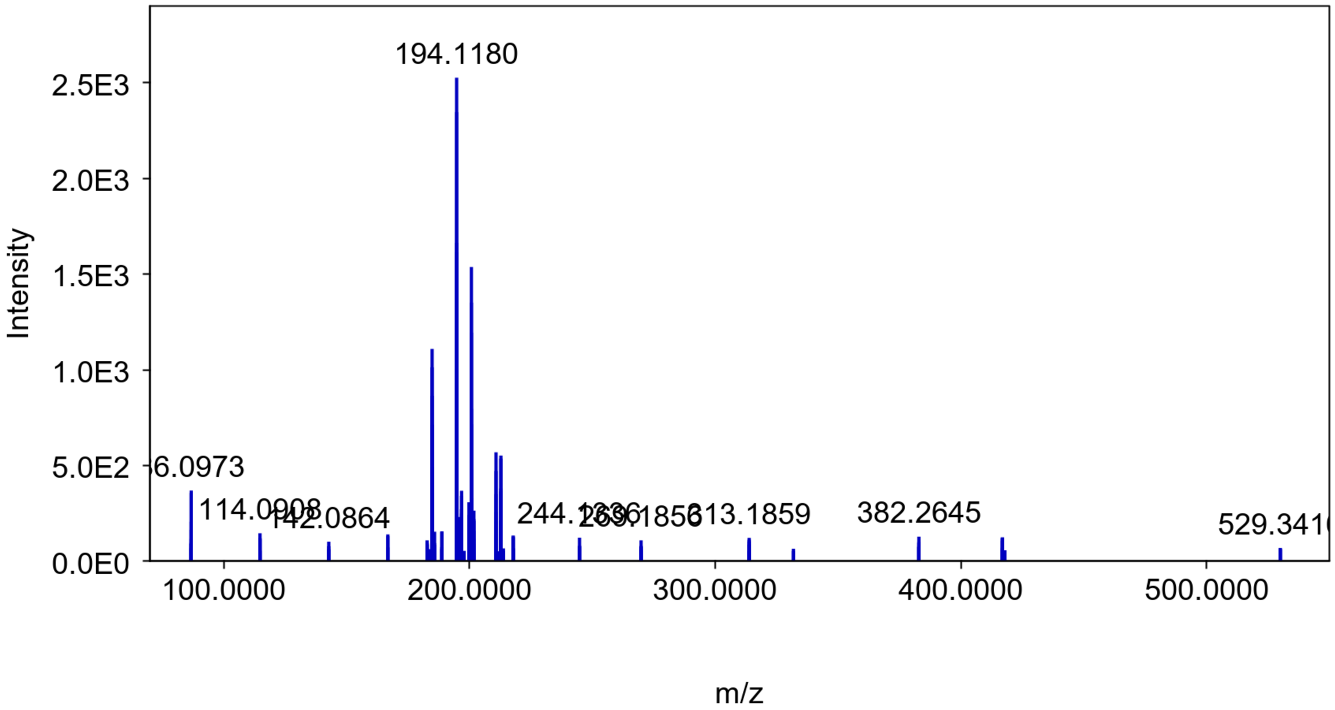

Figure S28. MS/MS fragmentation of psuedodestruxin C (**60**).

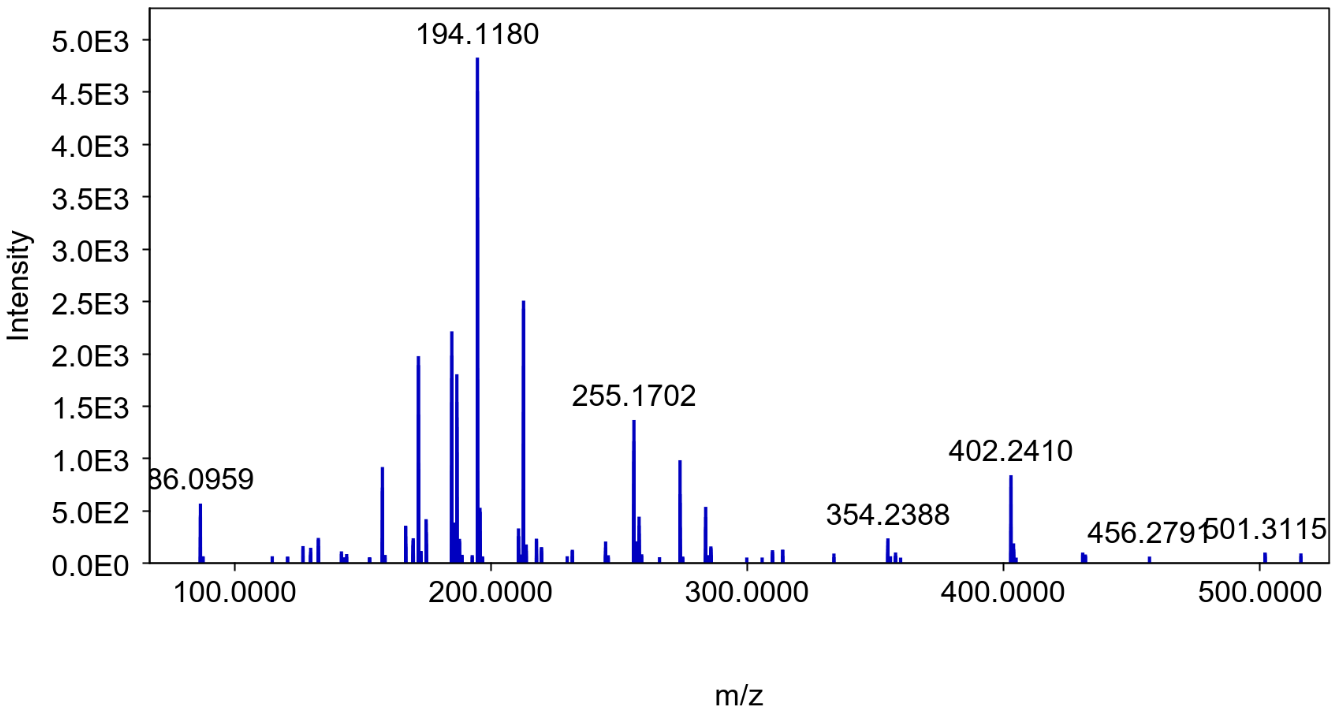

Figure S29. MS/MS fragmentation of desmethylisaridin E (**61**).

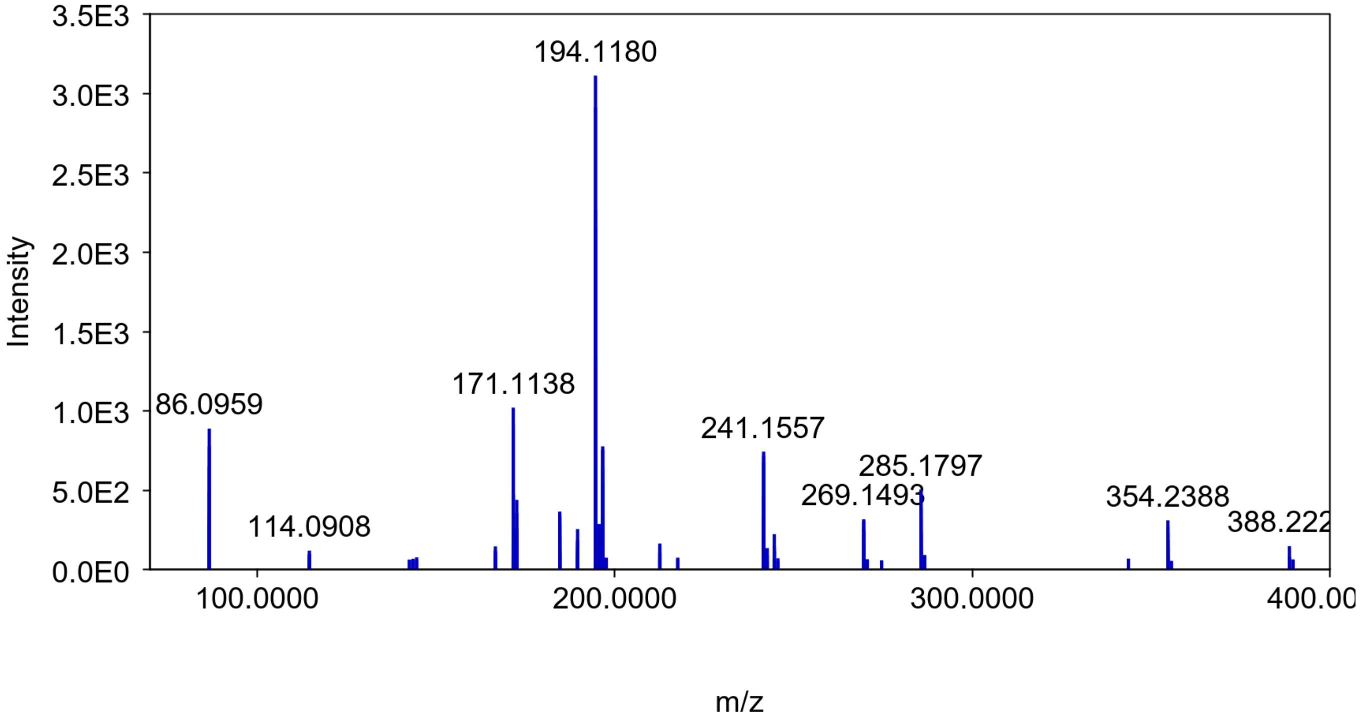

Figure S30. MS/MS fragmentation of isaridin F (**62**).

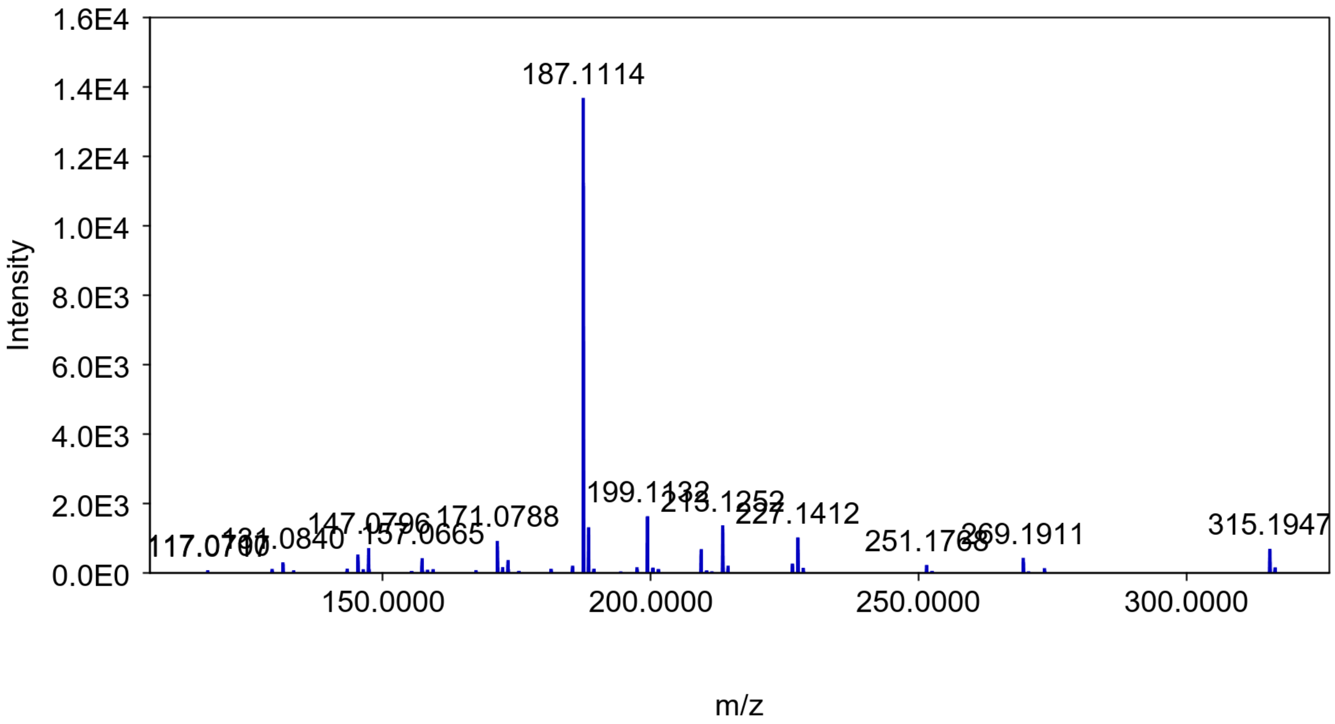

Figure S31. MS/MS fragmentation of 1,4a-dimethyl-9-oxo-7-propan-2-yl-3,4,10,10a-tetrahydro-2H-phenanthrene-1-carboxylic acid (**63**).

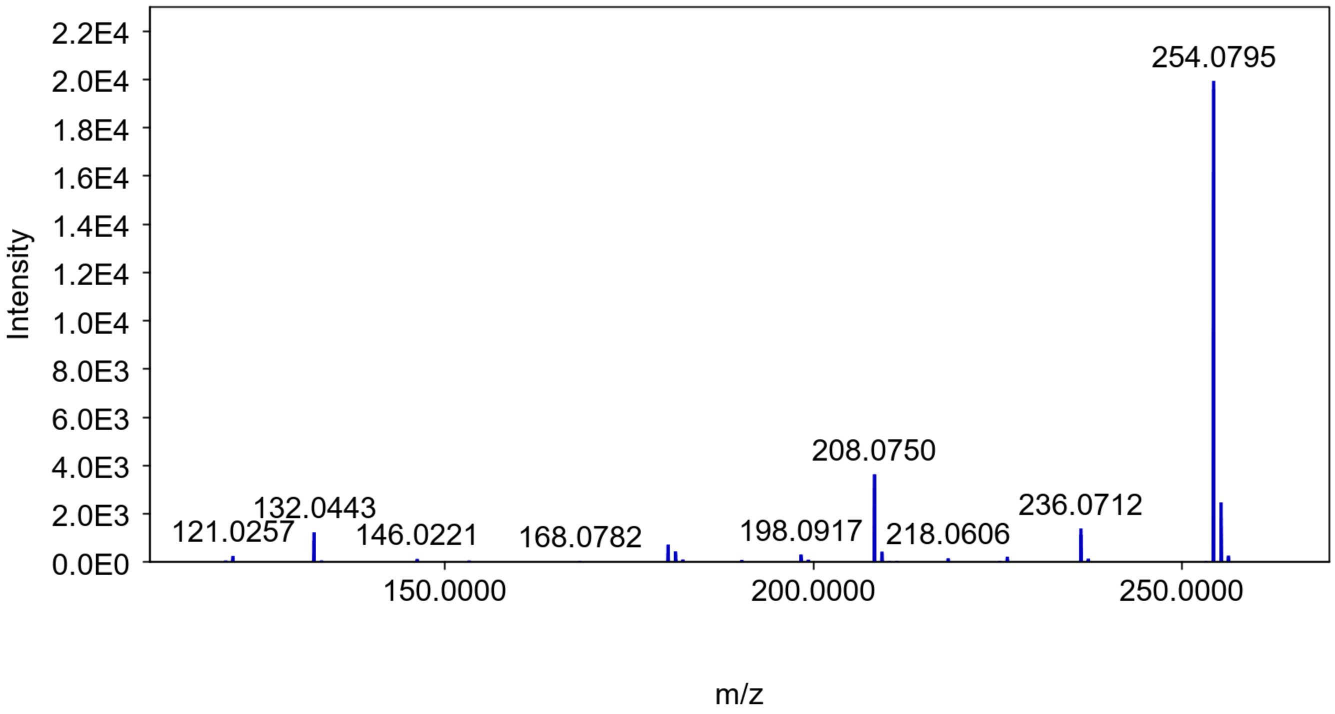

Figure S32. MS/MS fragmentation of viridicatol (**64**).

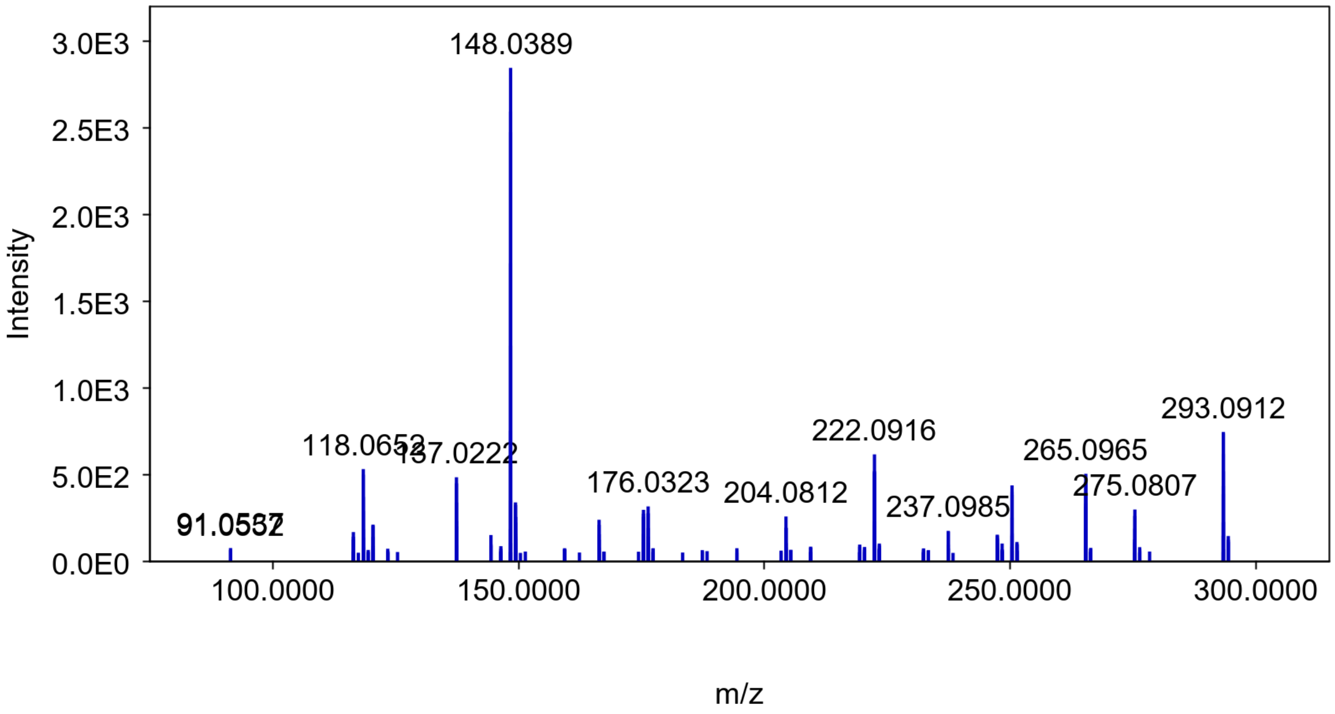

Figure S33. MS/MS fragmentation of citriperazine D (**75**).

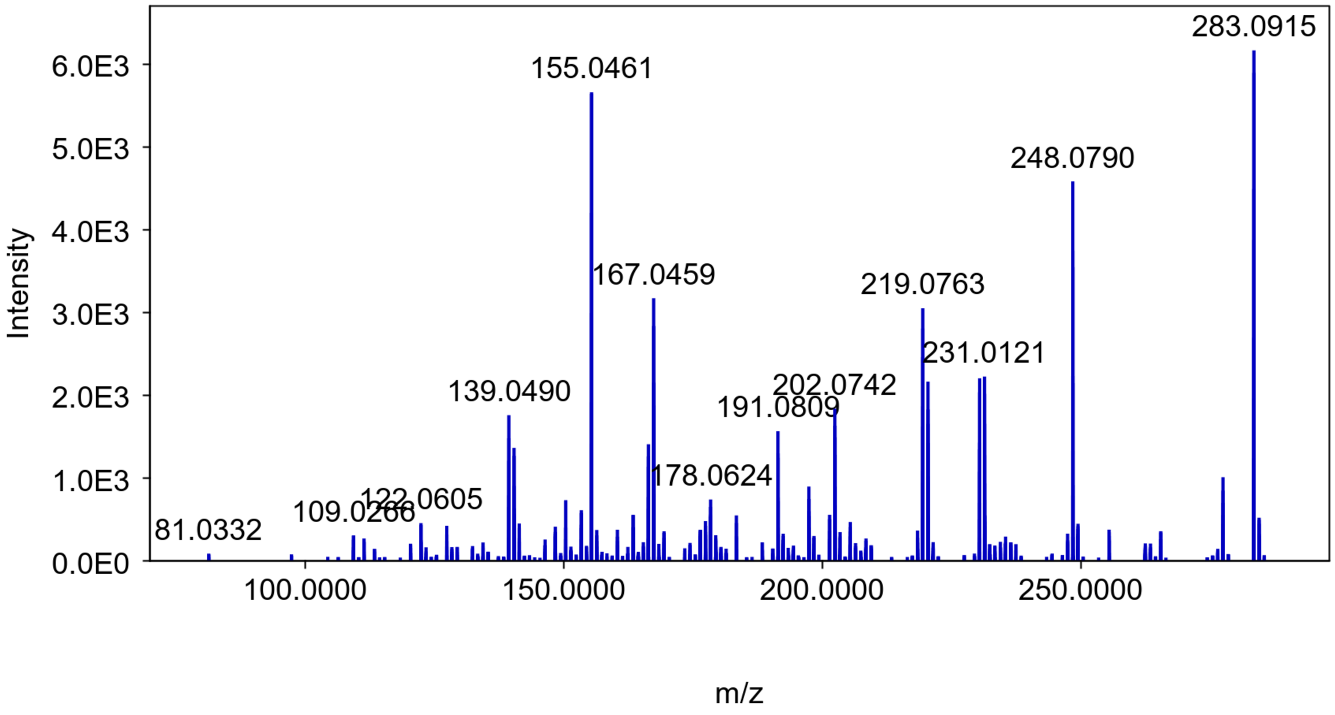

Figure S34. MS/MS fragmentation of pretrichodermamide C (**76**).

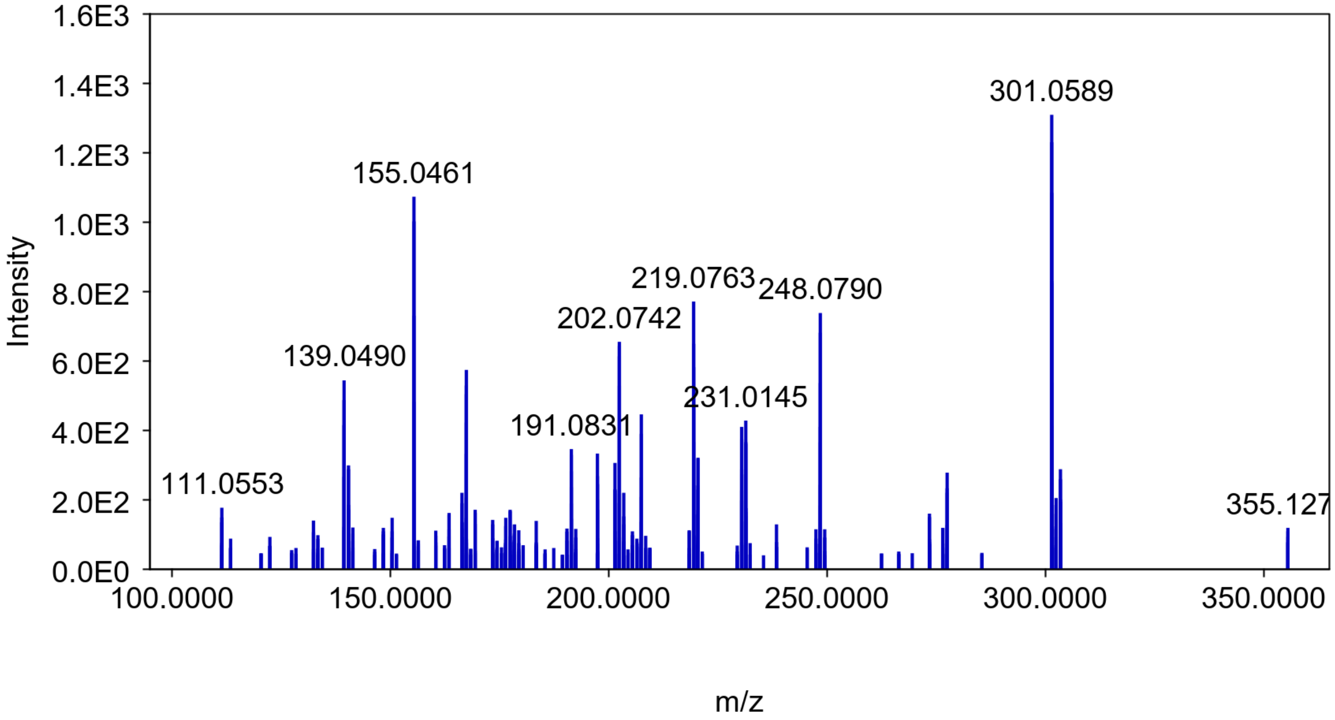

Figure S35. MS/MS fragmentation of N-methylpretrichodermamide B (**77**).

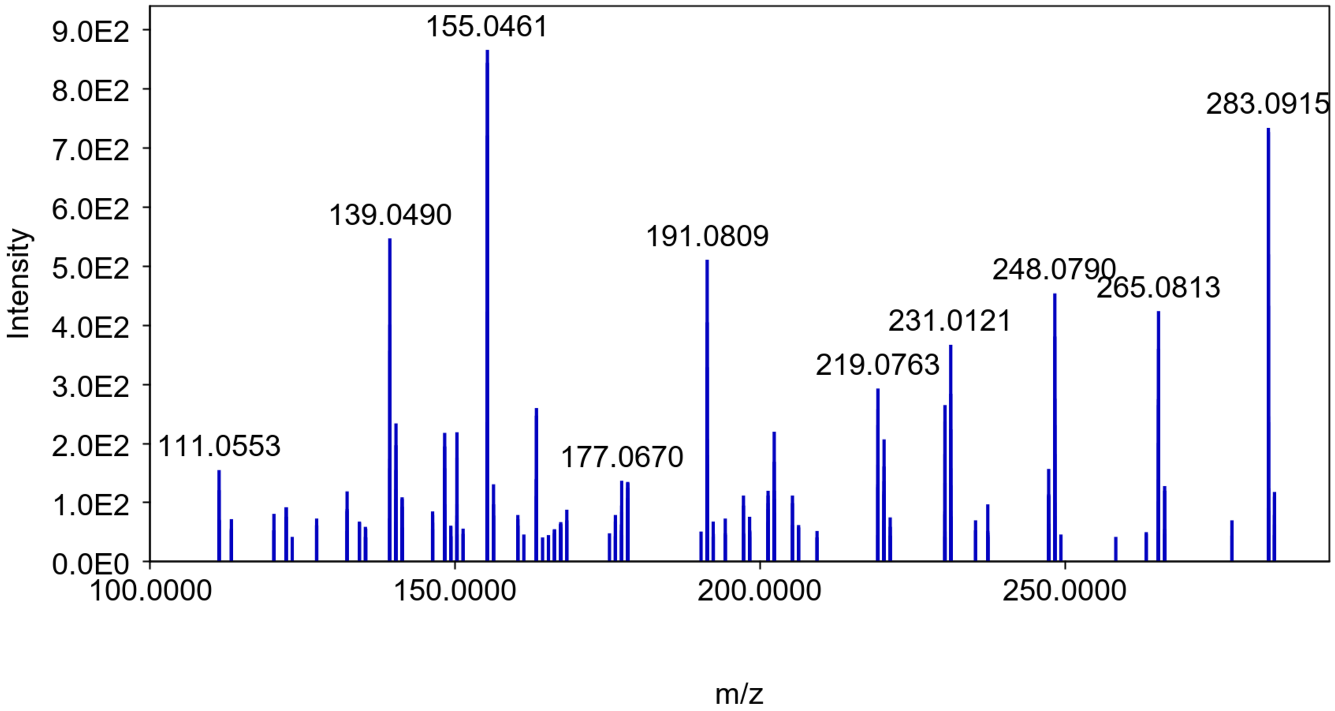

Figure S36. MS/MS fragmentation of pretrichodermamide D (**78**).

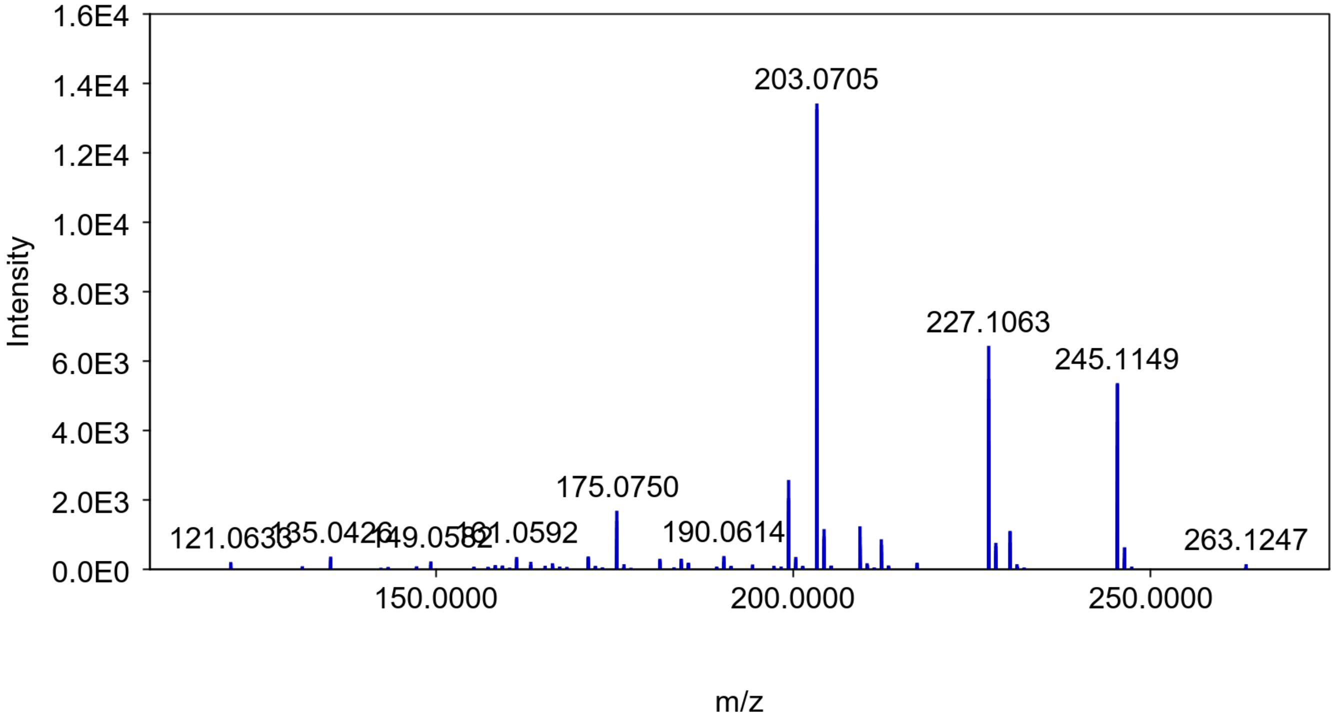

Figure S37. MS/MS fragmentation of coniothyrinone B (**86**).

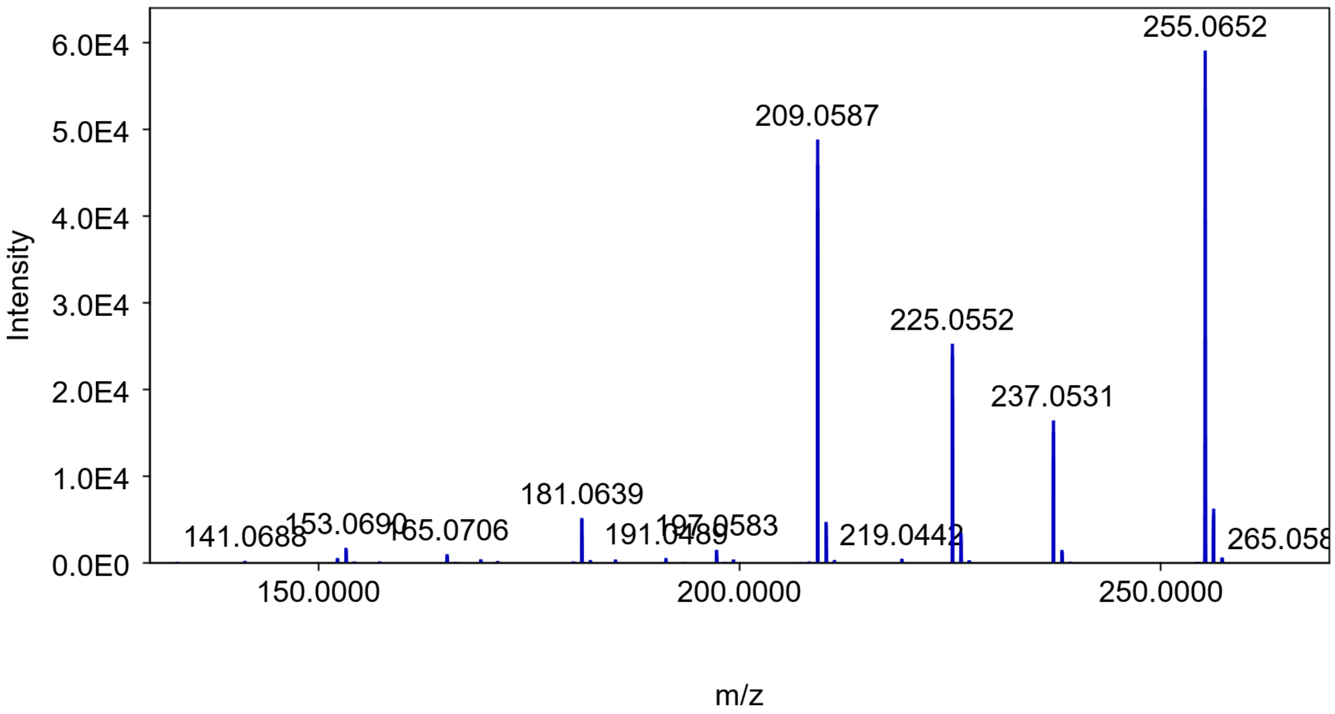

Figure S38. MS/MS fragmentation of 9,10-anthracenedione (**88**).

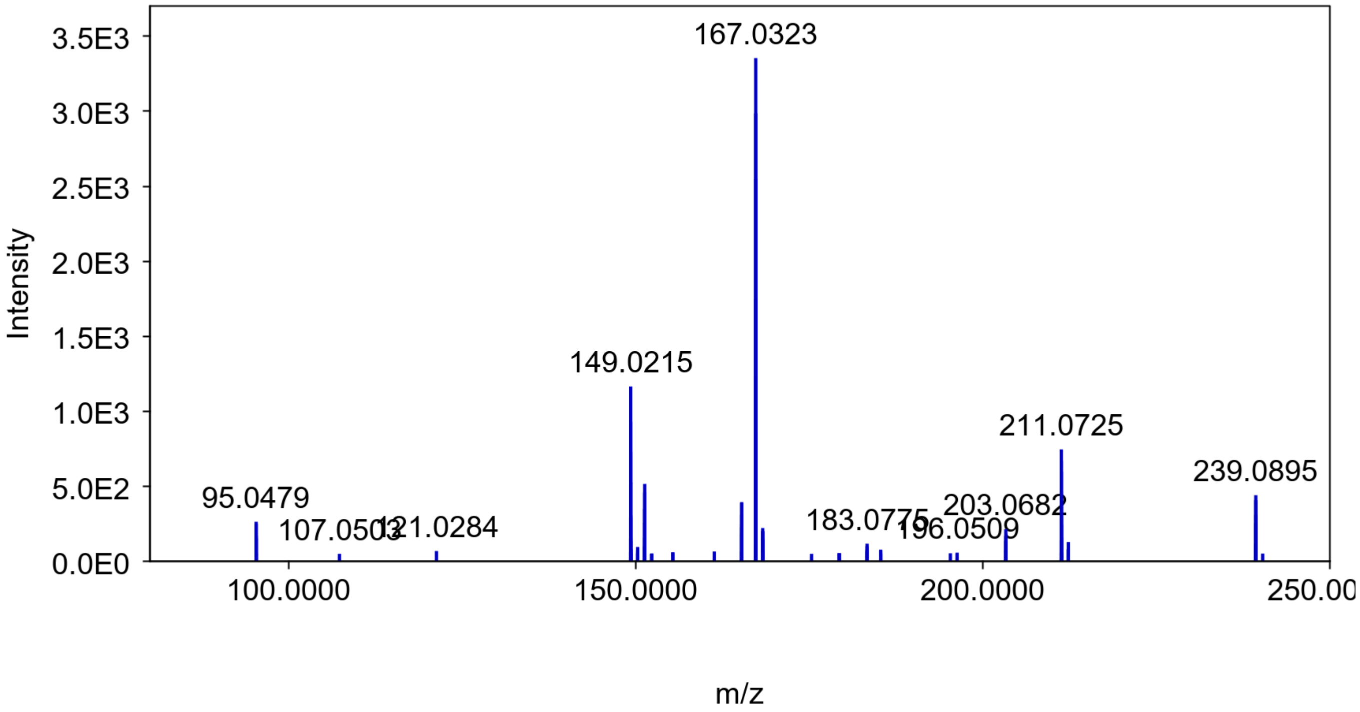

Figure S39. MS/MS fragmentation of trans-3,4-dihydroxy-3,4-dihydroanofinic acid (**89**).

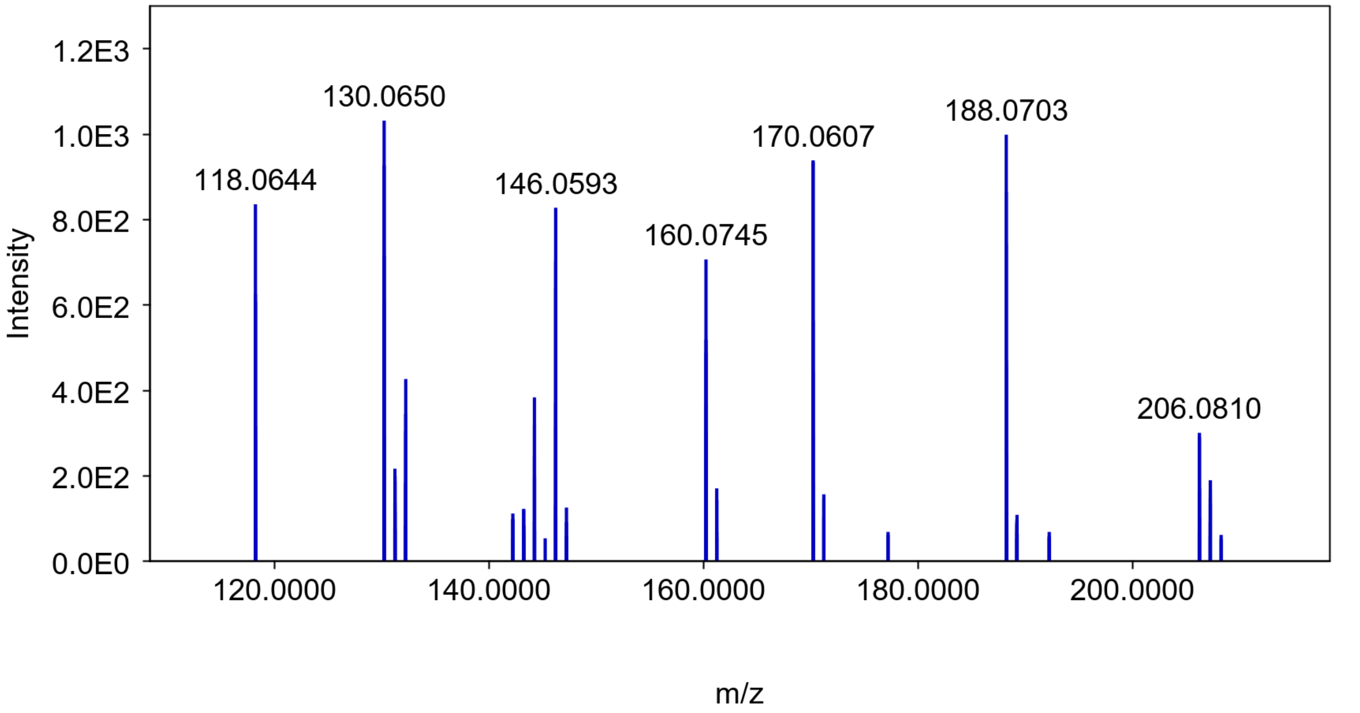

Figure S40. MS/MS fragmentation of indolelactic acid (**94**).

Table S1. Experimental UPLC MS data for reference compounds

N	Name	RT	Measured main peak	Calcd $[M+H]^+$
1	verruculogen	15.1	512.2400 $[M+H]^+$	512.2391
2	fumitremorgin C	10.1	380.1971 $[M+H]^+$	380.1969
5	tryptoquivaline J	8.0	403.1399 $[M+H]^+$	403.1401
7	spirotriprostatin A	10.4	396.1917 $[M+H]^+$	396.1918
8	6-methoxyspirotriprostatin B	7.6	394.1756 [M+H]+	394.1761
9	tryptoquivaline F	7.8	403.1398 [M+H]+	403.1401
20	ergosterol peroxide	20.4	429.3348 [M+H]+	429.3363
24	16α-hydroxy-17β-methoxy-deoxydihydroisoaustamide	7.3	396.1921 $[M+H]^+$	396.1918
25	16β-hydroxy-17α-methoxy-deoxydihydroisoaustamide	7.3	396.1921 $[M+H]^+$	396.1918
26	16α-hydroxy-17α-methoxy-deoxydihydroisoaustamide	7.3	396.1921 $[M+H]^+$	396.1918
27	16,17-dihydroxy-deoxydihydroisoaustamide	6.2	382.1748 $[M+H]^+$	382.1761
28	16β,17α-dihydroxy-deoxydihydroisoaustamide	6.0	382.1748 $[M+H]^+$	382.1761
29	16α,17α-dihydroxy-deoxydihydroisoaustamide	6.6	382.1748 $[M+H]^+$	382.1761
30	3β-hydroxydeoxyisoaustamide	2.9	364.1655 $[M+H]^+$	364.1656
31	deoxy-14,15-dehydroisoaustamide	10.8	346.1544 $[M+H]^+$	346.1550
32	(+)-deoxyisoaustamide	7.7	348.1713 $[M+H]^+$	348.1707
33	desoxybrevianamide E	9.6	352.2019 $[M+H]^+$	352.2020
45	oxirapentyn B	9.7	335.1496 [M+H]+	335.1489
46	oxirapentyn E	7.2	275.1280 $[M-H_2O+H]^+$	275.1278
47	oxirapentyn F	7.1	353.1593 [M+H]+	353.1595
48	oxirapentyn G	2.2	311.1504 [M+H]+	311.1489

49	oxirapentyn H	2.9	371.1702 [M+H]+	371.1700
50	oxirapentyn I	2.9	371.1702 [M+H]+	371.1700
51	oxirapentyn J	6.2	353.1589 [M+H]+	353.1595
52	isariketide A	5.7	313.0911 [M+H]+	313.0918
53	acremine S	3.7	207.1014 [M+H]+	207.1016
54	diorcin	10.4	231.1011 [M+H]+	231.1016
66	quinolactacide	5.4	237.0665 $[M+H]^+$	237.0659
67	3,5-dimethyl-8-meth-oxy-3,4-dihydro-1H-iso-chromene-6-ol	8.7	209.1171 [M+H]+	209.1172
68	anserinone B	4.6	193.0856 [M-H_2O+H]+	193.0859
69	formylanserinone B	7.5	239.0915 [M+H]+	239.0914
70	6-methyl curvulinic acid	5.3	207.0650 [M–H_2O+H]$^+$	207.0652
71	4-methoxyisoquinolin-1(2H)-one	2.6	176.0702 $[M+H]^+$	176.0706
72	N,N-diethyl-3-methylbenzamide	9.7	192.1390 $[M+H]^+$	192.1383
73	4-hydroxy-3,6-dimethyl-2-pyrone	2.7	141.0548 [M+H]+	141.0546
74	hydroxy-N-acetyl-β-ox-otriptamine	2.4	233.0924 [M+H]+	233.0921
75	citriperazine D	4.4	339.0975 [M-H_2O+H]+	339.0976
76	pretrichodermamide C	5.4	513.0990 [M+H]+	513.1996
77	N-methylpretrichoder-mamide B	8.1	531.0647 [M+H]+	531.0657
78	pretrichodermamide D	5.9	513.0990 [M+H]+	513.0996
79	4-hydroxyscytalone	2.2	211.0604 [M+H]+	211.0601
80	4-hydroxy-6-dehy-droxyscytalone	3.0	195.0654 [M+H]+	195.0652
81	3-methylorsellinic acid	2.6	183.0659	183.0652
82	acruciquinone A	6.4	277.1067 [M+H]+	277.1071
83	acruciquinone C	4.6	279.1227 $[M+H]^+$	279.1227

84	pleosporon	3.8	291.0855 [M+H]+	291.0863
85	coniothyrinone D	4.9	279.1226 [M+H]+	279.1227
86	coniothyrinone B	7.0	263.1277 [M+H]+	263.1278
87	rubrumol	5.2	277.1064 [M+H]+	277.1071
88	9,10-anthracenedione	10.1	255.0651 [M+H]+	255.0652
89	trans-3,4-dihydroxy-3,4-dihydroanofinic acid	4.9	239.0909 [M+H]+	239.0914
90	quadricinctapyran A	7.8	221.0815 [M+H]+	221.0808
91	7-hydroxymethyl-1,2-naphthalenediol	6.2	191.0706 [M+H]+	191.0703
92	gliovictin	8.3	377.0962 [M+Na]+	377.0964
93	acrucipentyn A	4.1	231.0781 [M+H]+	231.0782
